# Chemistry of 3-cyanoacetyl indoles: synthesis, reactions and applications: a recent update

**DOI:** 10.1039/d3ra04385a

**Published:** 2023-07-19

**Authors:** Abolfazl Olyaei, Mahdieh Sadeghpour

**Affiliations:** a Department of Chemistry, Payame Noor University (PNU) PO BOX 19395-4697 Tehran Iran; b Department of Chemistry, Qazvin Branch, Islamic Azad University Qazvin Iran mahdieh.sadeghpour@qiau.ac.ir +0098-28-33365275

## Abstract

Indole is a significant nitrogen-based heterocycle with particular importance in the synthesis of heterocyclic scaffolds. Indole based compounds have been recently attracting much attention due to their biological and pharmaceutical activities. 3-Substituted indoles such as cyanoacetyl indoles (CAIs) are nitrogen-heterocyclic compounds which are easily obtained from the reaction of indoles and cyanoacetic acid. They are versatile starting materials utilized for the construction of a wide variety of molecules containing indole moieties in organic synthesis. In this study, we provide an overview on the synthesis of 3-cyanoacetyl indoles (CAIs) and their recent applications in the multi-component reactions for the synthesis of various heterocyclic compounds such as pyranes, pyridines, dihydropyridines, pyrimidines, tetrahydropyrimidines, pyrazoles, pyrazolopyridines, pyrazolopyrimidines, pyridopyrimidines, tetrazolopyrimidines, triazolopyrimidines, furans, dihydrofurans, coumarins, pyrimido naphthyridines, chromenes, thiazoles, pyrimidoindazoles, pyrazoloquinolines, isoxazolopyridines, and carbazoles and their biological activities during the period of 2013 to 2022.

## Abstract 2

Indole is a significant nitrogen-based heterocycle with particular importance in the synthesis of heterocyclic scaffolds.

## Graphical abstract

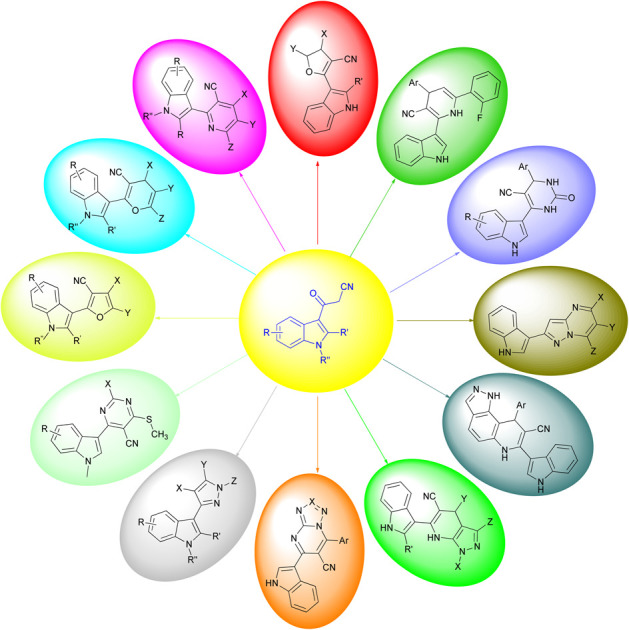

## 1 Introduction

**Fig. 1:**
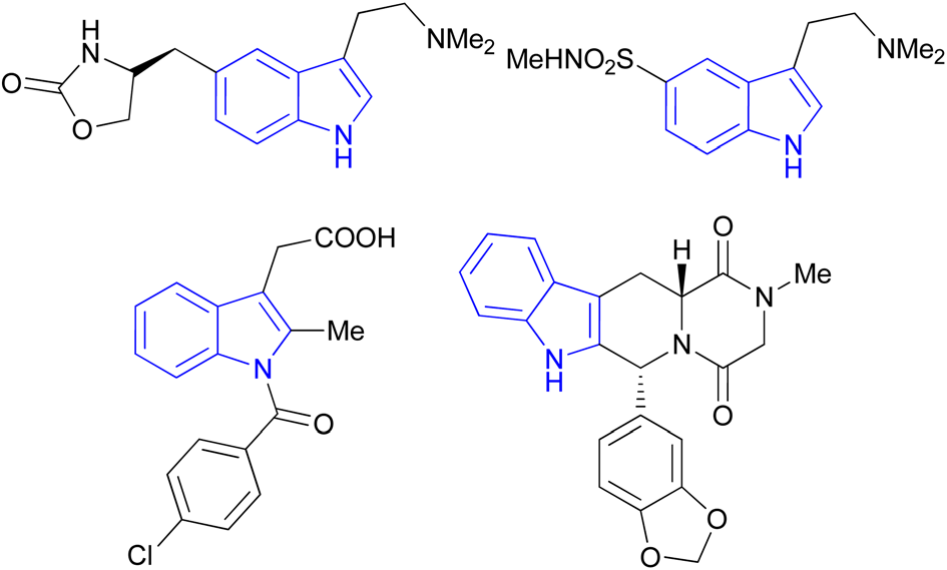
The molecular structure of four drugs with indole moieties.

**Fig. 2:**
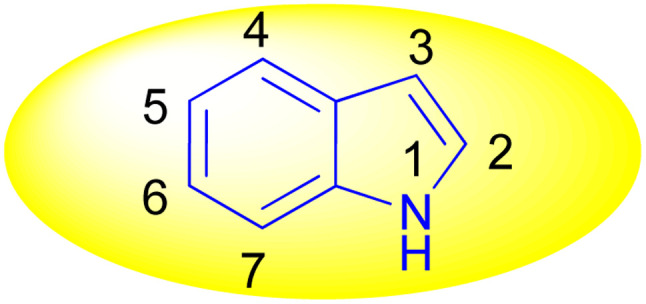
Structure of indole.

Indole is also known as benzopyrrole which contains a bicyclic aromatic heterocyclic organic compound comprising a six membered benzene ring fused to a five-membered nitrogen-containing pyrrole ring and has 10 π-electrons which makes it aromatic in nature. Indole is a well-known privileged structure scaffold occurring in numerous natural products such as alkaloids and peptides, existing in different kinds of plants, animals and marine organisms.^1^ Moreover, the addition of the indole nucleus to medicinal compounds that are biologically active made it an important heterocyclic compound having broad-spectrum biological and pharmaceutical activities (Fig. 1).^2–7^ Due to this, researchers took interest to synthesize various scaffolds of indole for screening different pharmacological activities. Electrophilic substitution occurs readily on indole due to excessive π-electron delocalization.^8^ Indole is reactive at four different positions including carbon atom 3,^9–13^ nitrogen atom 1, the C2–C3 π-bond^14–17^ and the C2–N sigma bond^18,19^ (Fig. 2). Indole can be protonated with strong acids, which protonates the C3 position, more easily than the N atom. The cycloaddition reaction is another reaction of indole compounds. The C2–C3 π-bond of indole has a propensity towards cycloaddition reactions but cycloaddition reactions of the C2–N sigma bond are also observed. However, various methods for the synthesis and functionalization of indoles have been reviewed.^20–24^ 3-Substituted indoles such as cyanoacetyl indoles (CAIs) are nitrogen-heterocyclic compounds which are versatile starting materials utilized for the construction of a wide variety of molecules containing indole moieties in organic synthesis. A large portion of these molecules exhibits promising biological activities such as against nicotinic acetylcholine receptors and prostaglandin-mediated disease,^25^ antimicrobial,^26^ antifungal,^27^ anti-inflammatory and analgesic activities,^28^ against prophylaxis and angiogenesis,^29^ anticancer^30,31^ and acts as inhibitors of JNK.^32^ Up to date, two review articles have been published based on the synthesis and reactions of 3-cyanoacetyl indoles.^33,34^ This review presents the recent applications of cyanoacetyl indoles in the synthesis of diverse organic compounds and their biological activities during the period from 2013 to 2022.

## 2. Synthesis of 3-cyanoacetyl indoles

**Scheme 1:**
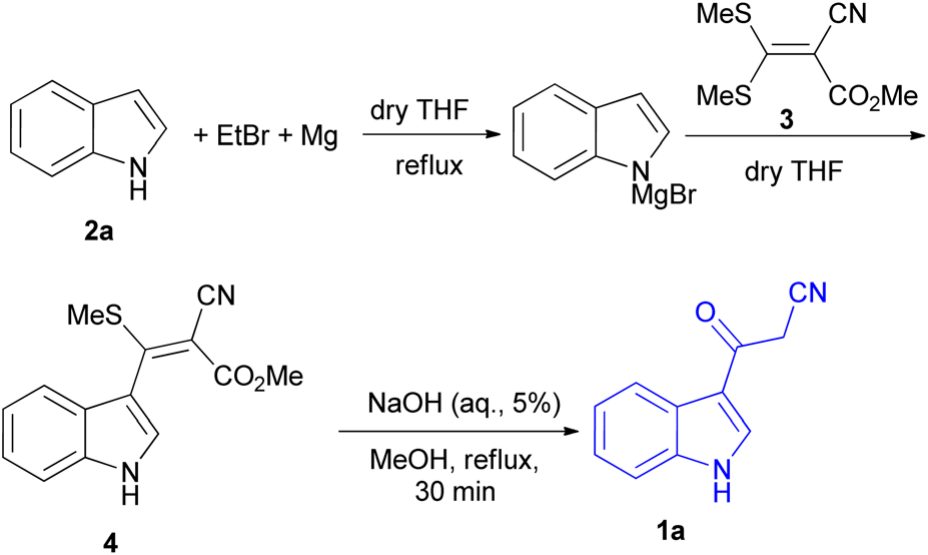
Synthesis of 3-cyanoacetyl indole (1a).

**Scheme 2:**
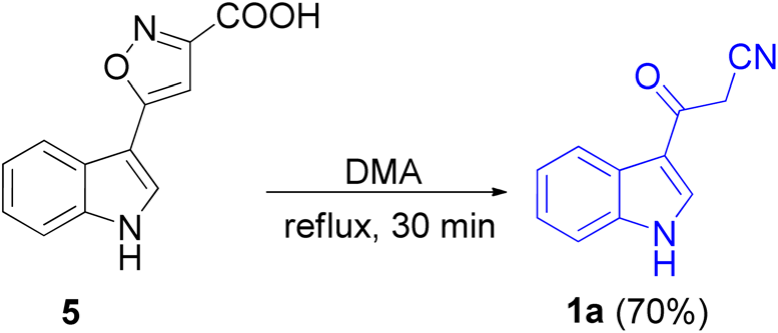
Preparation of 3-cyanoacetyl indole (1a).

**Scheme 3:**
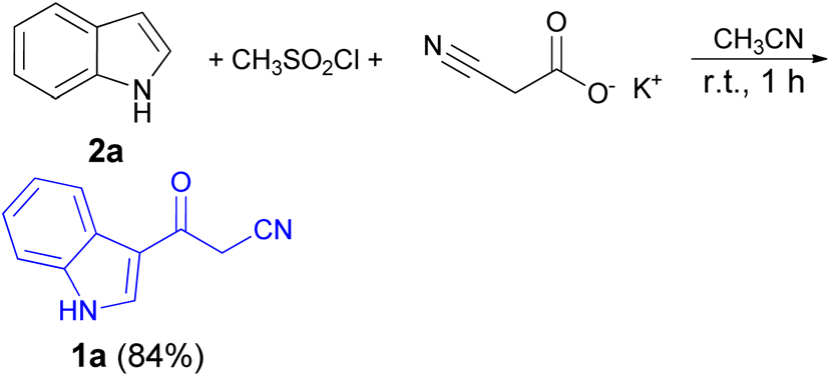
Synthesis of 3-cyanoacetyl indole (1a).

**Scheme 4:**
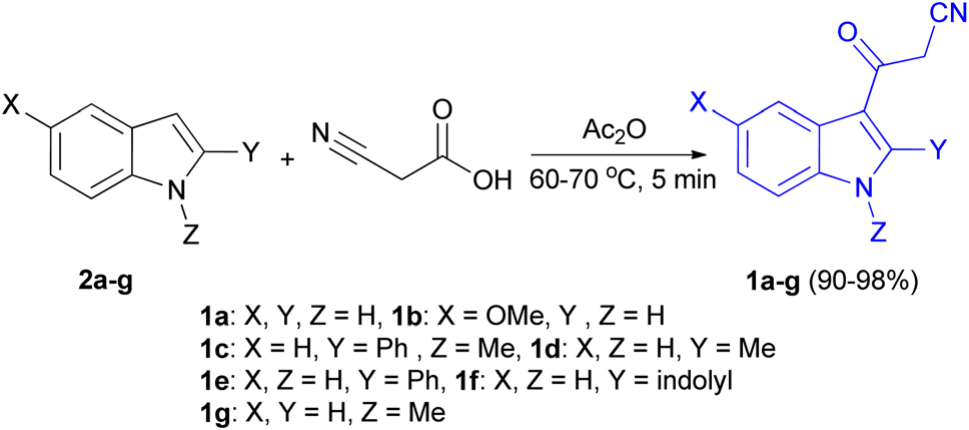
Synthesis of 3-cyanoacetyl indoles 1a–g.

In 1967, the first synthesis of 3-cyanoacetyl indole (1a) was reported by Washida and co-workers where an indole (2a) in the presence of Grignard reagent and the keten thioacetal 3, followed by hydrolysis of the intermediate 4 in 5% NaOH solution afforded 1a (Scheme 1).^35^ In 1978, Gorbunova and Suvorov described synthesis of 1a in 70% yield by heating of 5-(3-indolyl)isoxazole-3-carboxylic acid 5 in dimethylacetamide (DMA) under reflux condition for 30 min (Scheme 2).^36^ In 1980, Kreher and Wagner provided a procedure for the synthesis 1a in 84% yield *via* the reaction of indole, methanesulfonyl chloride and potassium cyanoacetate in acetonitrile at room temperature for 1 h (Scheme 3).^37^ Next, Bergman *et al.* developed the use of acetic anhydride in the synthesis of 3-cyanoacetyl indole derivatives 1a–g in 90–98% yields from the reaction of indoles 2 with cyanoacetic acid at 60–70 °C for 5 min (Scheme 4).^38^

**Scheme 5:**
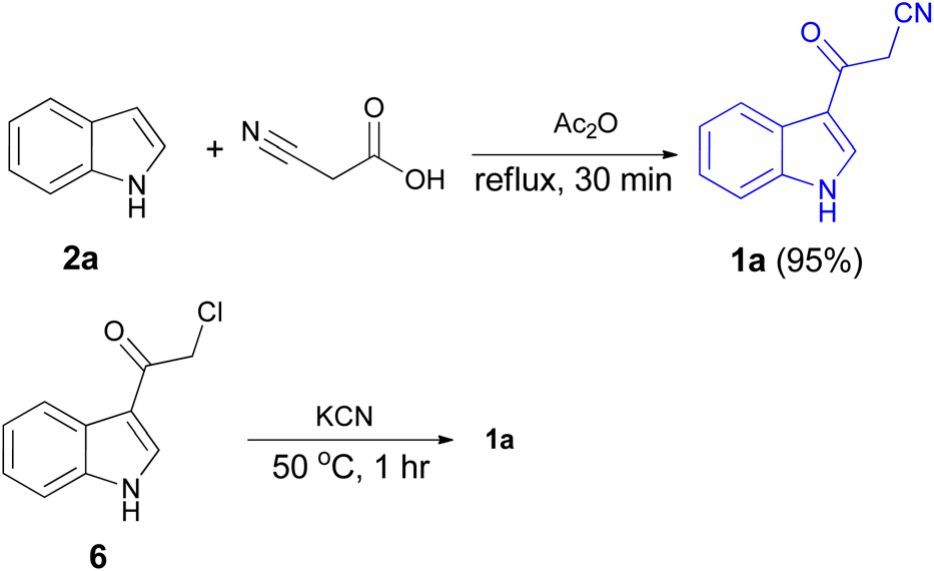
Synthesis of 3-cyanoacetyl indole (1a).

After that, 3-cyanoacetyl indole (1a) obtained *via* the reaction of cyanoacetic acid with indole in refluxing acetic anhydride for 30 min. Moreover, chloroacetylindole 6 readily converted into 1a on treatment with potassium cyanide at 50 °C for 1 h (Scheme 5).^39^

**Scheme 6:**
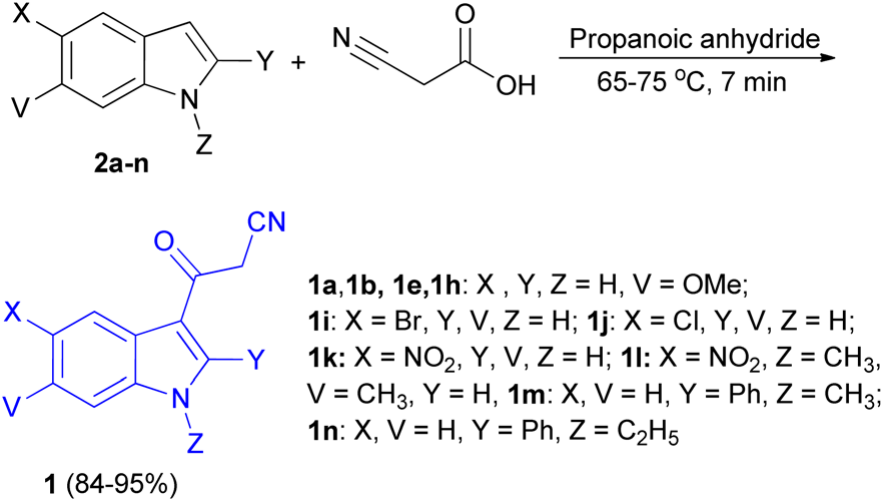
Synthesis of 3-cyanoacetyl indole derivatives 1.

In 2013, a facial method for the synthesis of 3-cyanoacetyl indole derivatives 1 in 84–95% yields reported by the reaction of indoles 2, cyanoacetic acid and propanoic anhydride at 65–75 °C for 7 min (Scheme 6).^40^

## 3. 3-Cyanoacetyl indoles reactions

### 3.1. Pyran derivatives

**Scheme 7:**
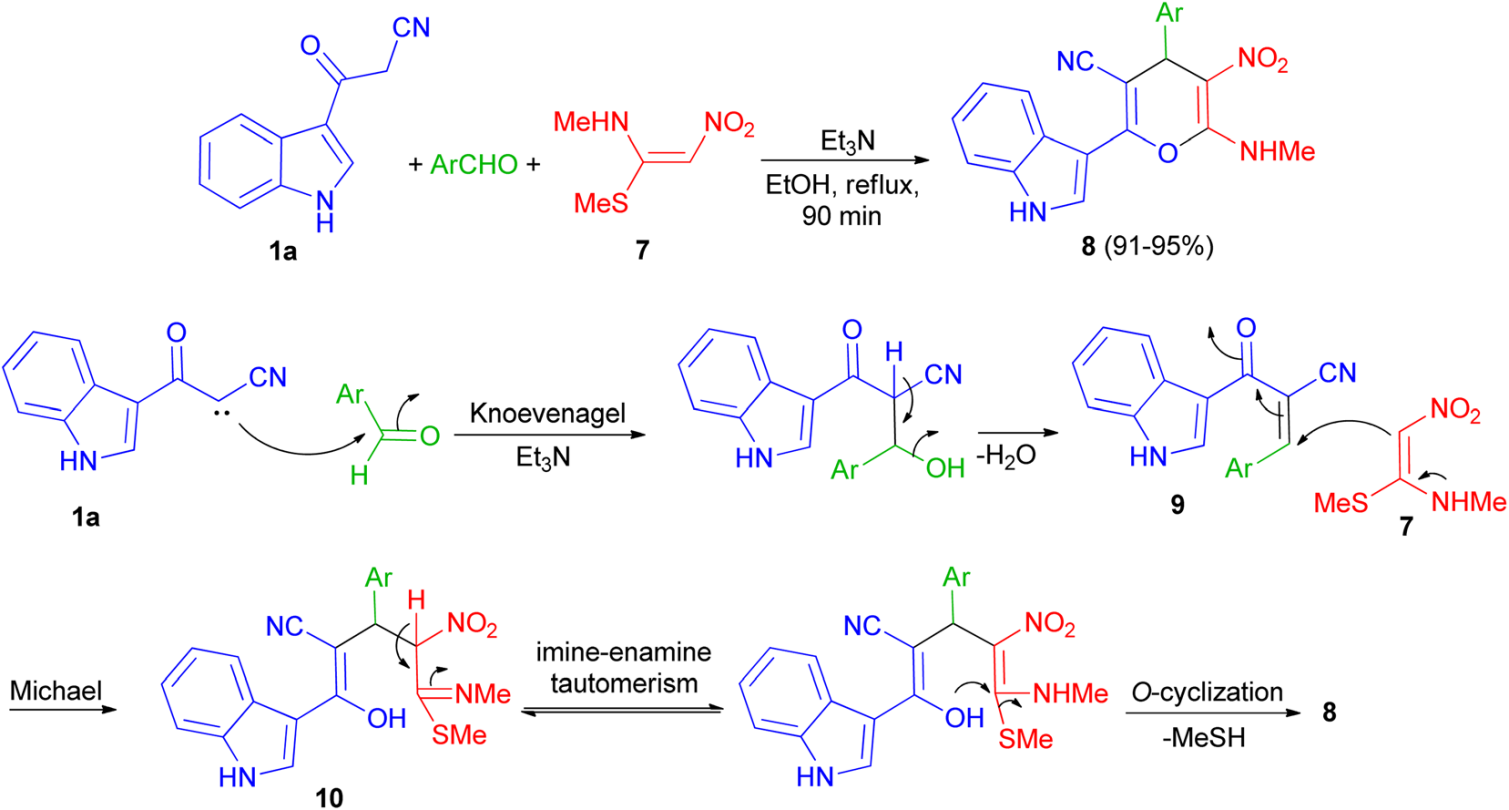
Synthesis of penta-substituted 4*H*-pyrans 8.

In 2013, Kumar and co-workers used 3-cyanoacetyl indole (1a), aromatic aldehydes and (*E*)-*N*-methyl-1-(methylthio)-2-nitroethenamine (7) for the synthesis of 2-(1*H*-indol-3-yl)-6-(methylamino)-5-nitro-4-aryl-4*H*-pyran-3-carbonitriles 8. The corresponding products produced in 91–95% yields in the presence of Et_3_N in EtOH under reflux conditions for 90 min. A plausible mechanistic pathway for the formation of 8 is outlined in Scheme 7. Initially, the Knoevenagel condensation between 1a and aromatic aldehyde affords 9, which undergoes Michael addition with 7 to give 10. The intermediate 10 is susceptible to either an intramolecular o-cyclization which can afford penta-substituted 4*H*-pyrans 8.^41^

**Scheme 8:**
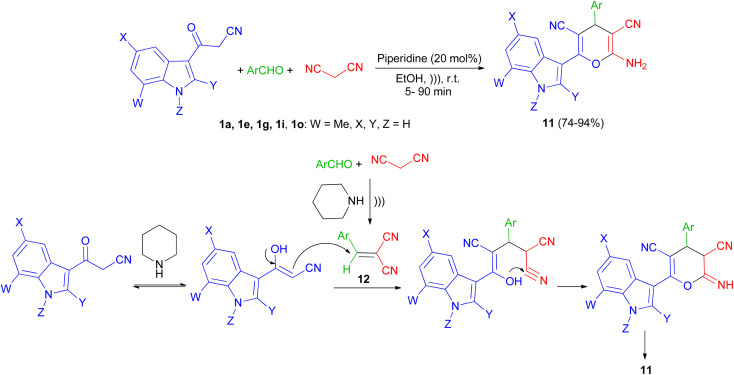
Synthesis of indol-3-yl substituted pyran derivatives 11.

Next, a series of polysubstituted indol-3-yl substituted pyran derivatives 11 synthesized in 74–94% yields *via* one-pot multi-component reactions of aromatic aldehydes, malononitrile with 3-cyanoacetyl indoles 1 in the presence of piperidine (20 mol%) in EtOH at room temperature under ultrasonic irradiation for 5–90 min. A plausible mechanism for the formation of 11 is shown in Scheme 8. Compound 11 could be produced from the intermediate 12*via* Michael addition with the tautomer of 3-cyanoacetyl indole 1 followed by intramolecular cyclization.^42^

**Scheme 9:**
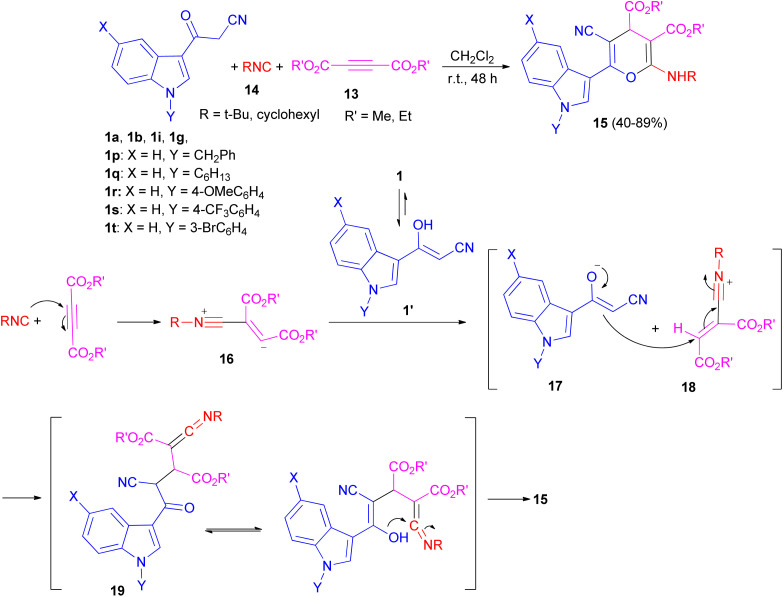
Synthesis of 6-(indol-3-yl)-4*H*-pyrans 15.

After that, Ji and co-workers reported an efficient multi-component reaction of 3-indolyl-3-oxopropanenitriles 1 with dialkyl acetylenedicarboxylates 13 and isocyanides 14 in CH_2_Cl_2_ under mild conditions leading to highly functionalized 6-(indol-3-yl)-4*H*-pyrans 15 in moderate to good yields (40–89%) for 48 h. A plausible mechanism was proposed as shown in Scheme 9. The reaction of isocyanide 14 and dialkyl acetylenedicarboxylate 13*in situ* leads to the formation of zwitterionic intermediate 16, which could be protonated by OH of 1′ to give intermediates 17 and 18. Subsequently, ketenimine 19 could be formed by the reaction of enolate 17 with the nitrilium ion 18. After tautomerization under the employed reaction conditions and cyclization, pyran derivative 15 is formed.^43^

**Scheme 10:**
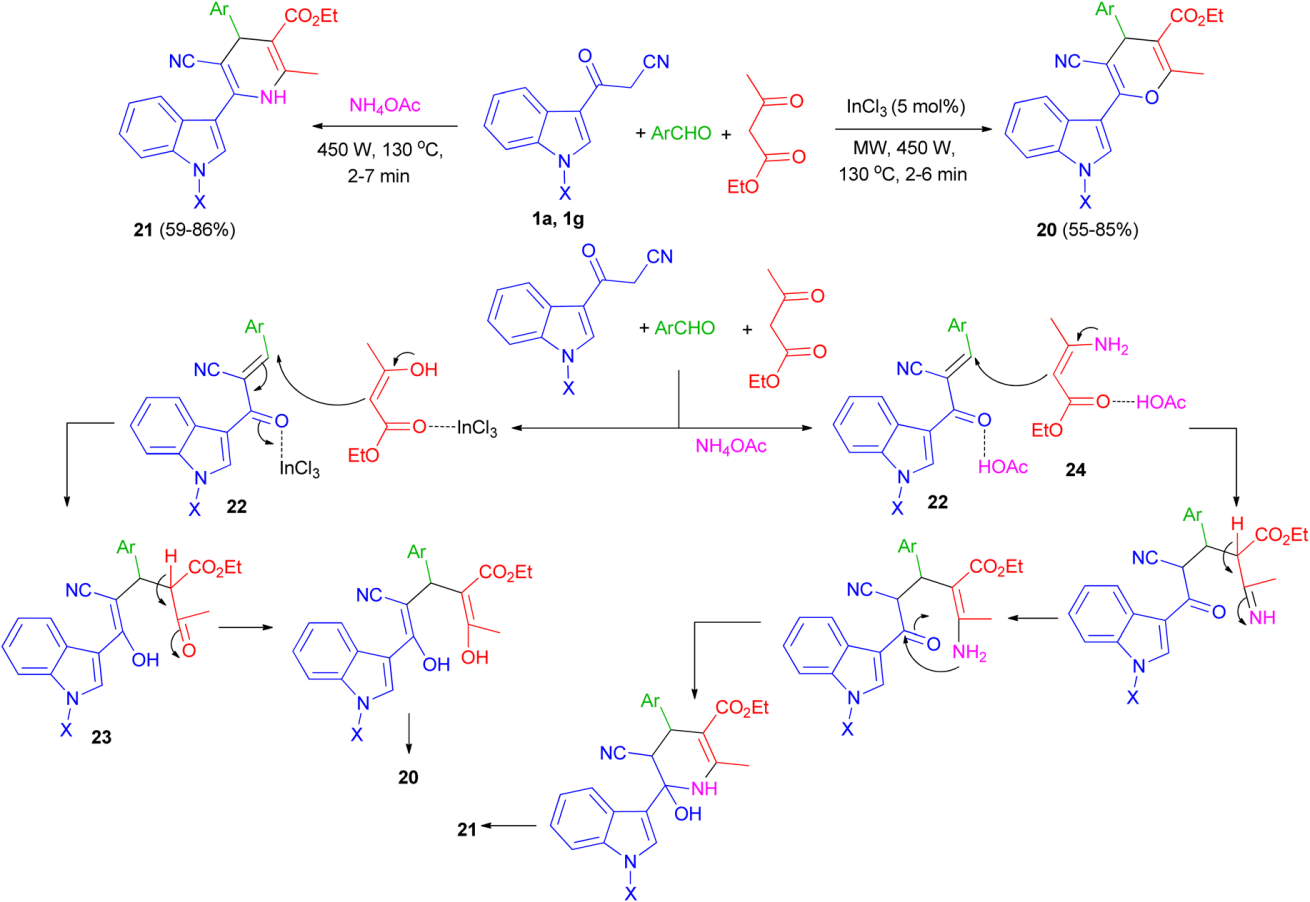
Synthesis of 3-(pyranyl)- and 3-(dihydropyridinyl)indole derivatives 20 and 21.

One-pot multi-component reaction of 3-cyanoacetyl indoles 1, aromatic aldehydes and ethyl acetoacetate in the presence of InCl_3_/NH_4_OAc under microwave irradiation (540 W, 130 °C) for 2–7 min afforded highly functionalized 3-(pyranyl)- and 3-(dihydropyridinyl)indole derivatives and 20, respectively, in good yields (55–86%). A plausible mechanism for the formation of compound 20 and 21 is shown in Scheme 10. 3-Cyanoacetyl indoles 1 reacts with aryl aldehyde to give a α,β-unsaturated ketone 22 which reacts with ethyl acetoacetate to give the Michael adduct 23 under microwave irradiation in the presence of InCl_3_ and subsequently cyclizes by eliminating water molecule to give the 3-(pyranyl)indole derivative 20. In the formation of compound 21, β-ketoester first reacts with ammonia, generated from the dissociation of ammonium acetate under MW, to give amine 24. The amine 24 then reacts with intermediate 22 to give the corresponding Michael adduct and finally undergoing cyclization/water elimination to afford product 21.^44^

**Scheme 11:**
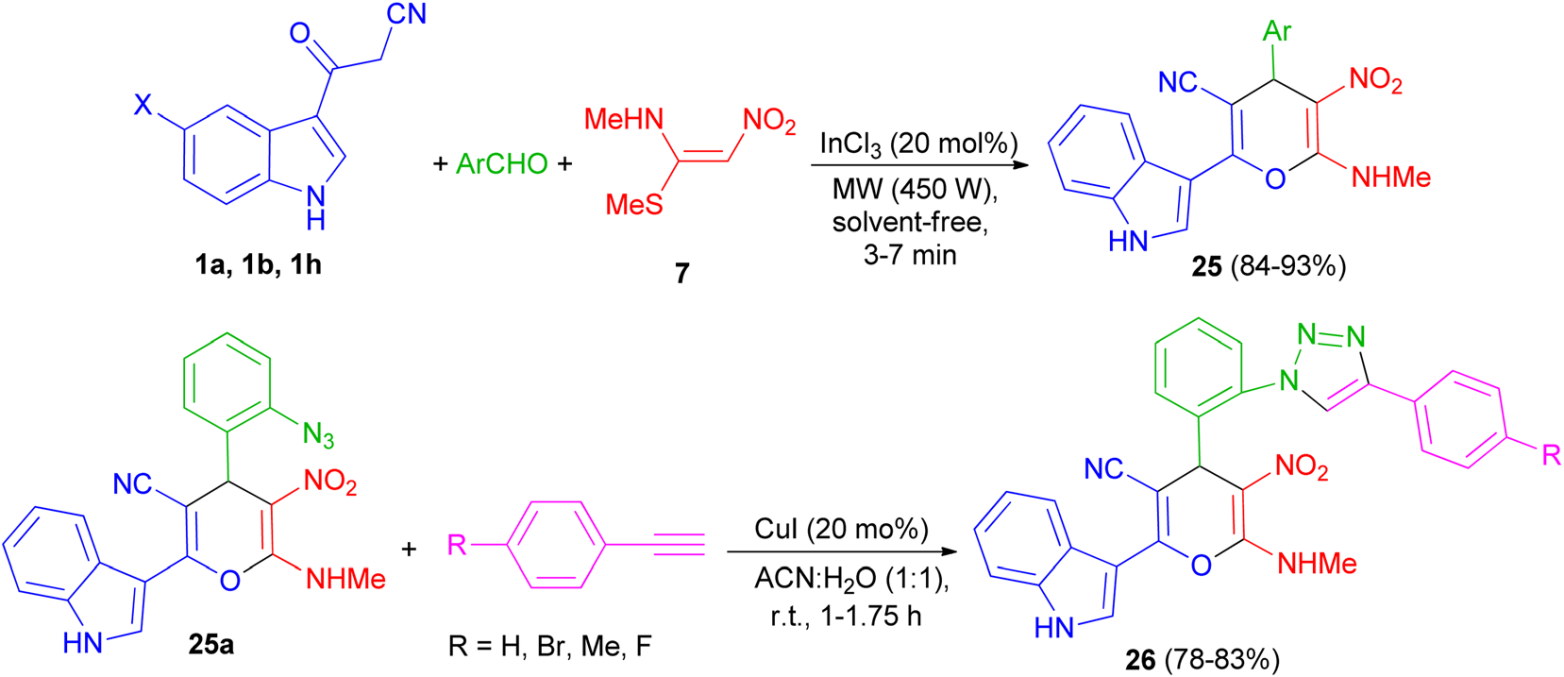
Microwave assisted InCl_3_ mediated synthesis of indolylpyrans 25 and indolyltriazolylpyran hybrids 26.

A series of highly functionalized indolylpyrans 25 has been synthesized in 84–93% yields *via* InCl_3_ (20 mol%) catalyzed microwave irradiation (450 W) of 3-cyanoacetylindoles 1, aromatic aldehydes with (*E*)-*N*-methyl-1-(methylthio)-2-nitroethenamine (NMSM) (7) under solvent-free condition for 3–7 min. Further, the synthesized azidoindolylpyrans 25a undergo [3 + 2] cycloaddition reaction with different phenyl acetylenes in the presence of CuI (20 mol%) as catalyst in ACN : H_2_O (1 : 1) at room temperature to give indolyltriazolylpyran hybrids 26 in 78–83% yields after 1–1.75 h (Scheme 11).^45^

**Scheme 12:**
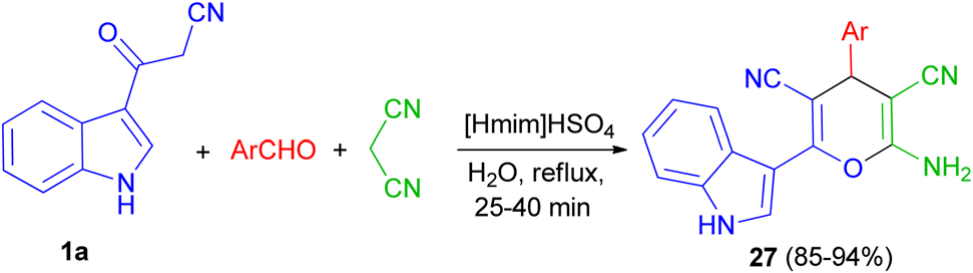
Synthesis of 3-pyranyl indole derivatives 27 by using ionic liquid.

Next, a green method was developed for one pot synthesis of 3-pyranyl indole derivatives 27 in 85–94% yields by reaction of 3-cyano acetyl indole (1a), aromatic aldehydes and malanonitrile in an aqueous media under reflux for 25–40 min by using [Hmim]HSO_4_ as a green and reusable catalyst (Scheme 12).^46^

**Scheme 13:**
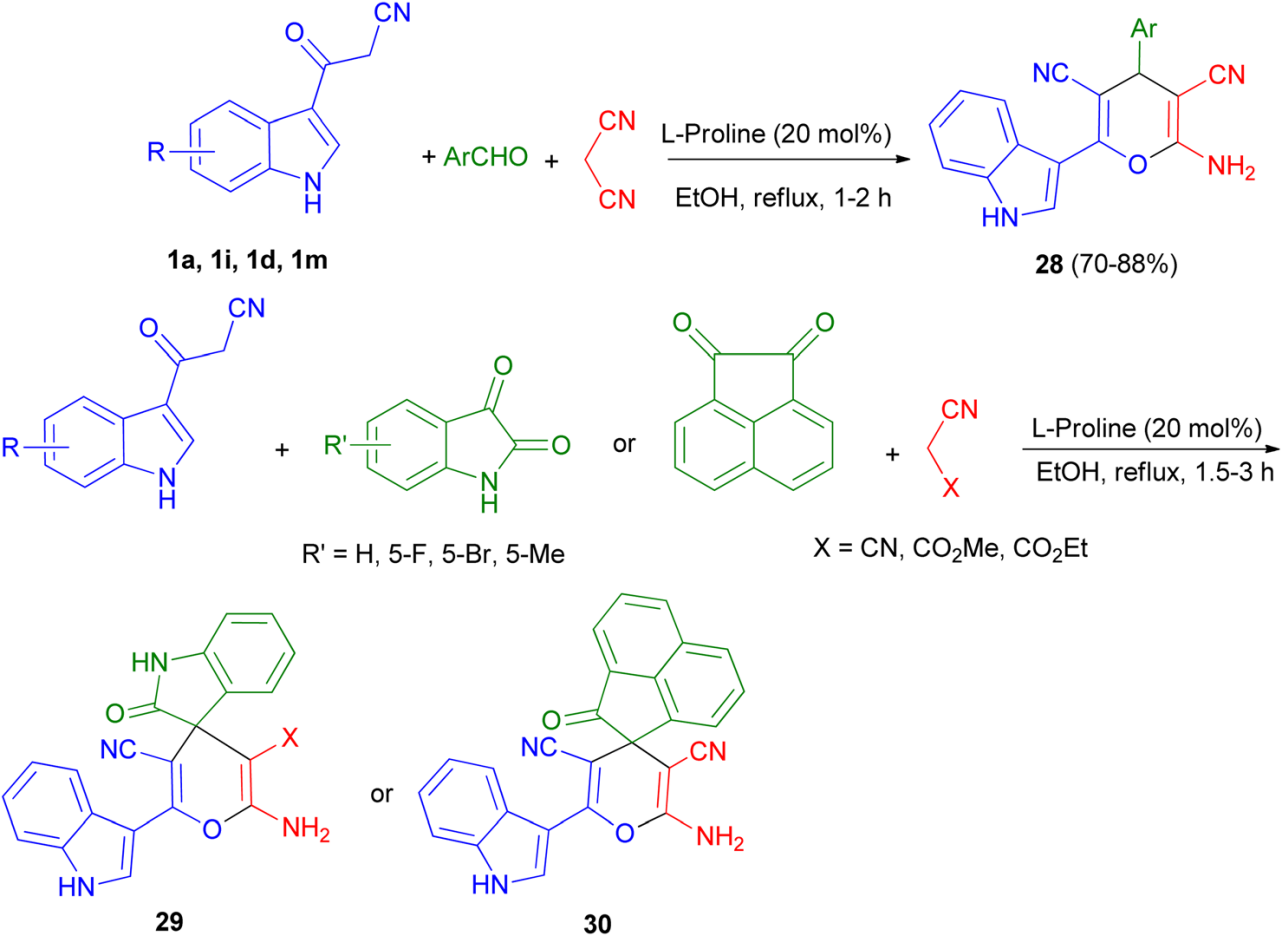
Preparation of functionalized indol-3-yl pyran derivatives 28–30.

Green synthetic method for preparation of functionalized indol-3-yl pyran derivatives 28–30 in 70–88% yields was developed by one-pot three-component synthesis of 3-cyanoacetyl indoles 1, malononitrile/cyanoacetate and various aldehydes/isatins/acenaphthenequinone in the presence of l-proline (20 mol%) in refluxing EtOH for 1–3 h (Scheme 13).^47^

**Scheme 14:**
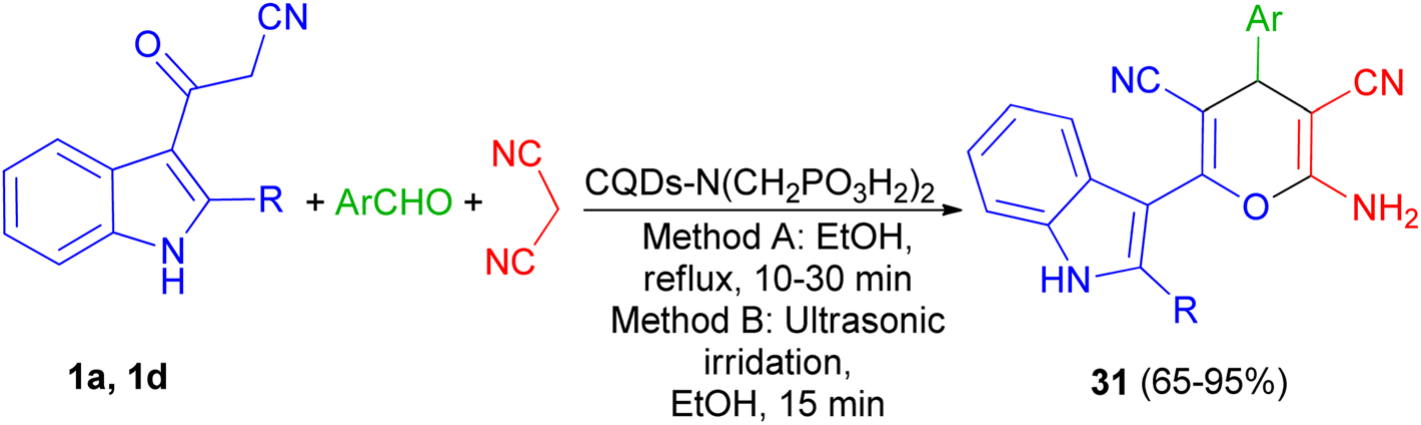
Synthesis of multisubstituted 4*H*-pyran with indole moieties 31.

In 2021, Zarei and his group described synthesis of 2-amino-6-(2-methyl-1*H*-indol-3-yl)-4-phenyl-4*H*-pyran-3,5-dicarbonitriles 31 in 65–95% yields by the one-pot reaction of various aromatic aldehydes, 3-(1*H*-indol-3-yl)-3-oxopropanenitrile derivatives 1 and malononitrile using CQDs-N(CH_2_PO_3_H_2_)_2_ as catalyst in refluxing EtOH and/or ultrasonic irradiation conditions (Scheme 14).^48^

**Scheme 15:**
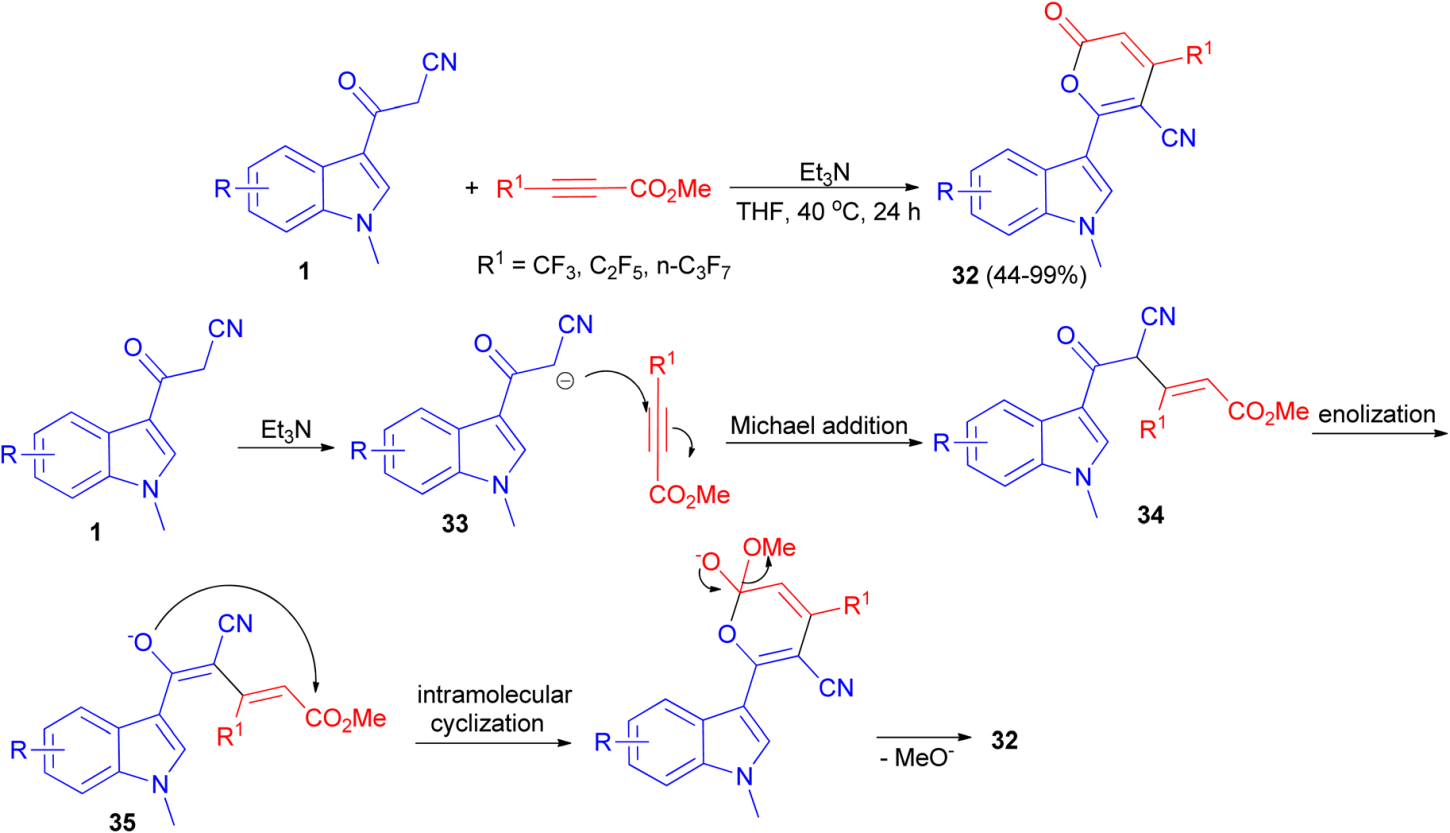
Synthesis of 4-perfluoroalkylated 2*H*-pyran-2-ones 32.

A protocol for the synthesis of 4-perfluoroalkylated 2*H*-pyran-2-ones 32 in 44–99% yields bearing indole skeleton reported *via* the reaction of 3-(1-methyl-1*H*-indol-3-yl)-3-oxopropanenitriles 1 and methyl perfluoroalk-2-ynoates in the presence of Et_3_N in THF at 40 °C in air for 24 h. A plausible reaction mechanism is proposed in Scheme 15. In the presence of the base, deprotonation of 1 generates the anion 33, which undergoes Michael addition to afford the intermediate 34 through an attack to the carbon atom at the β-position of the alkyne, which has a less electron density compared to the carbon atom at α-position. Subsequent enolization of 34 leads to the formation of oxygen anion 35, followed by intramolecular oxygen nucleophilic attack to carbonyl carbon to accomplish the cyclization, and final elimination of methoxide anion furnishes the target product 32.^49^

### 3.2. Pyridine and dihydropyridine derivatives

**Scheme 16:**
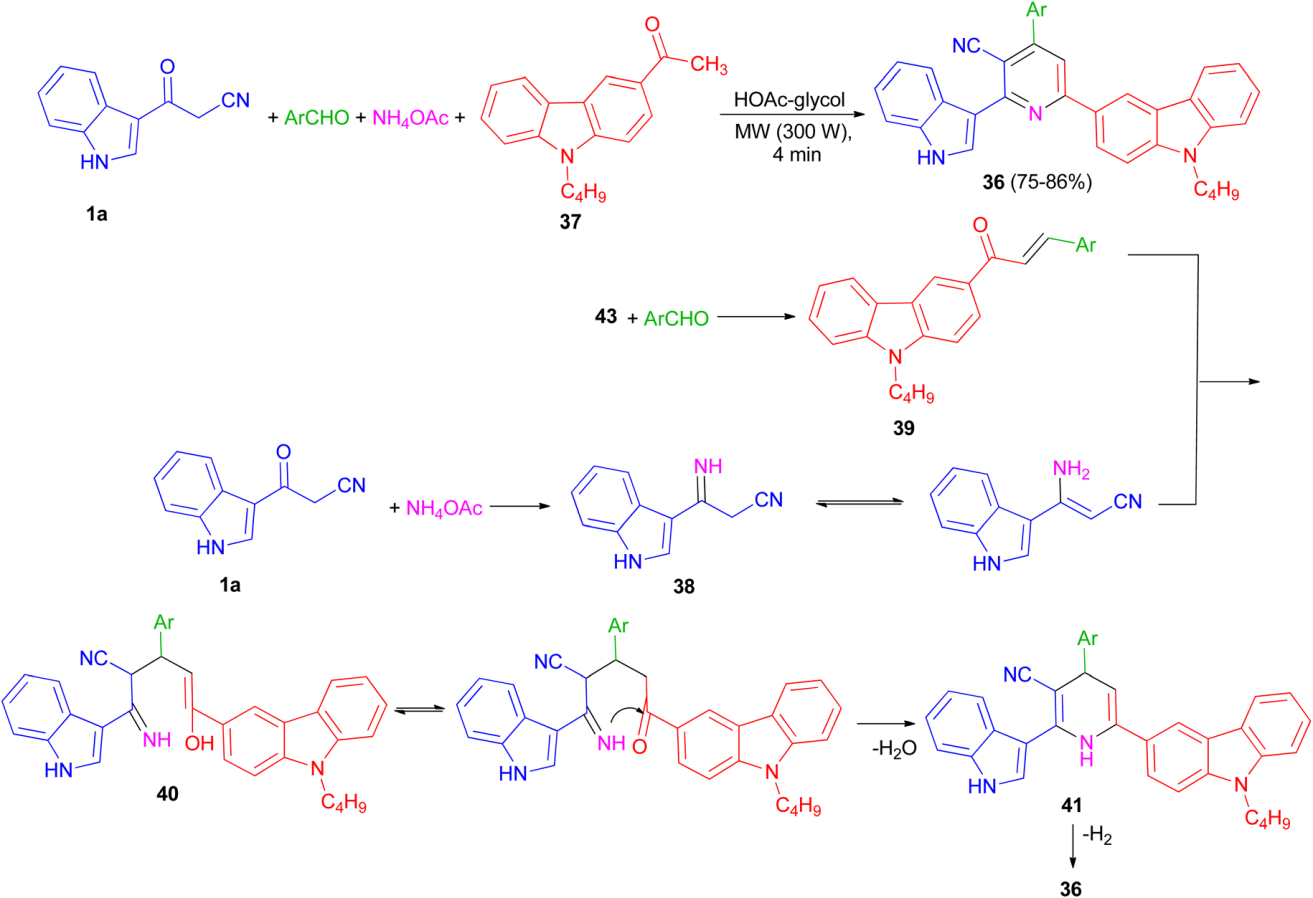
Synthesis of 3-cyano-2-(1*H*-indol-3-yl)-6-(9-butylcarbazol-3-yl)pyridines 36.

In 2013, preparation of a series of 3-cyano-2-(1*H*-indol-3-yl)-6-(9-butylcarbazol-3-yl)pyridine derivatives 36 in 75–86% yields reported through the one-pot four-component coupling of aromatic aldehydes, 1-(9-butylcarbazol-3-yl)ethanone (37), 3-(cyanoacetyl)indole (1a) and ammonium acetate in HOAc-glycol under irradiation at 300 W for 4 min. A possible mechanism is depicted in Scheme 16. 3-(Cyanoacetyl)indole reacts with ammonia from ammonium acetate to give intermediate 38, which further reacts with the corresponding chalcones 39, to yield 40. Michael addition product 40 was then cyclised to afford the Hantzsch dihydropyridine derivative 41 with elimination of water.

Subsequent dehydrogenation of 41 leads to formation of the highly substituted pyridine derivative 36.^50^

**Scheme 17:**
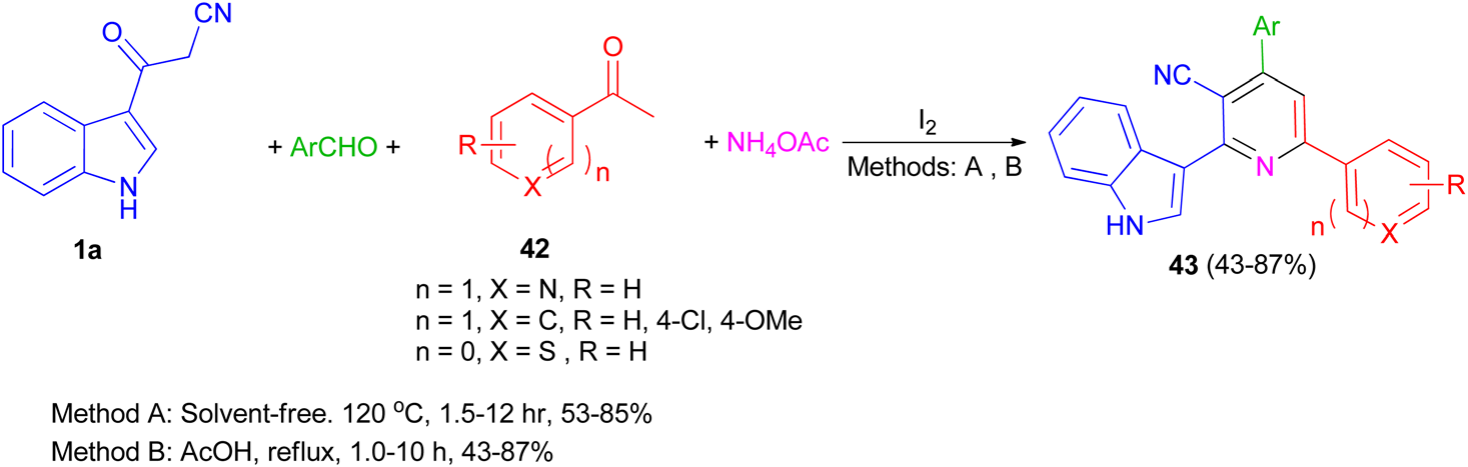
Preparation of 2-(indol-3-yl)pyridine derivatives 43.

A one-pot four-component reaction of 1a, aromatic aldehydes, aromatic ketones 42, and NH_4_OAc in the presence of iodine as a catalyst was explored for the preparation of 2-(indol-3-yl)pyridine derivatives 43 in 43–87% yields under two different conditions (solvent-free and using AcOH as solvent) (Scheme 17).^51^

**Scheme 18:**
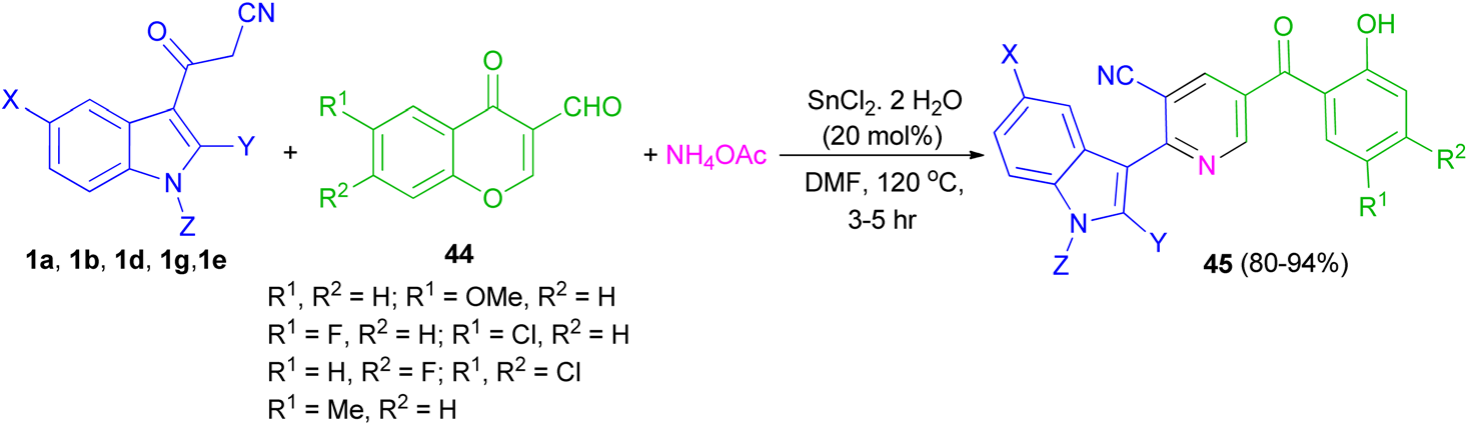
Synthesis of functionalized indole-3-yl pyridines 45.

Perumal *et al.* explored the one-pot three-component reaction of 3-formylchromones 44, cyanoacetylindoles 1 and ammonium acetate for the synthesis of functionalized indole-3-yl pyridines 45 in 80–94% yields in the presence of stannous chloride in DMF at 120 °C for 3–5 h (Scheme 18).^52^

**Scheme 19:**
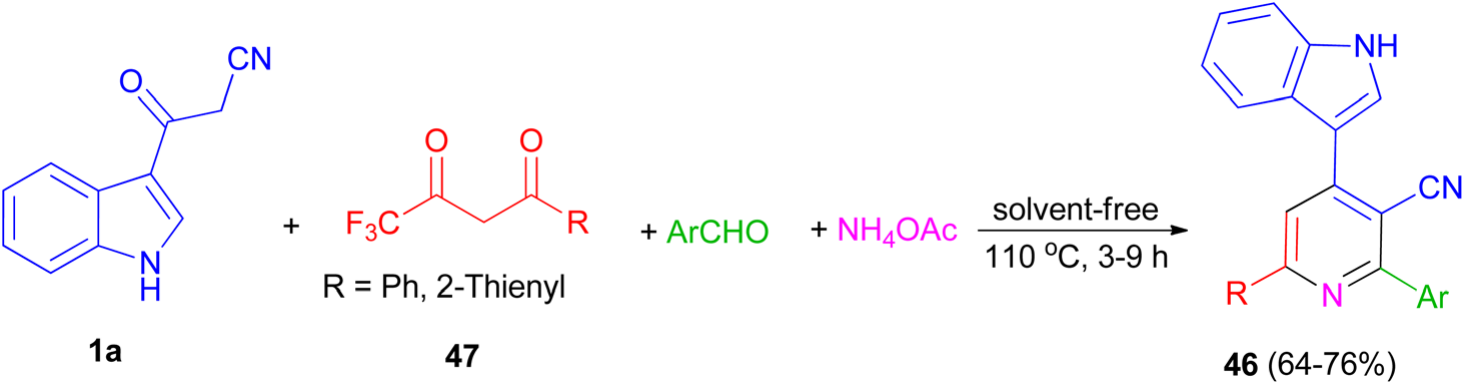
Synthesis of 2-aryl-4-(1*H*-indol-3-yl)-6-phenyl-3-cyanopyridines 46.

In 2014, a series of 2,6-diaryl-4-(1*H*-indol-3-yl)-3-cyanopyridines 46 was obtained in good yields (64–76%) from the domino reactions of 1a, 4,4,4-trifluoro-1-arylbutane-1,3-diones 47, and aromatic aldehydes in the presence of ammonium acetate under solvent-free condition at 110 °C for 3–9 h (Scheme 19).^53^

**Scheme 20:**
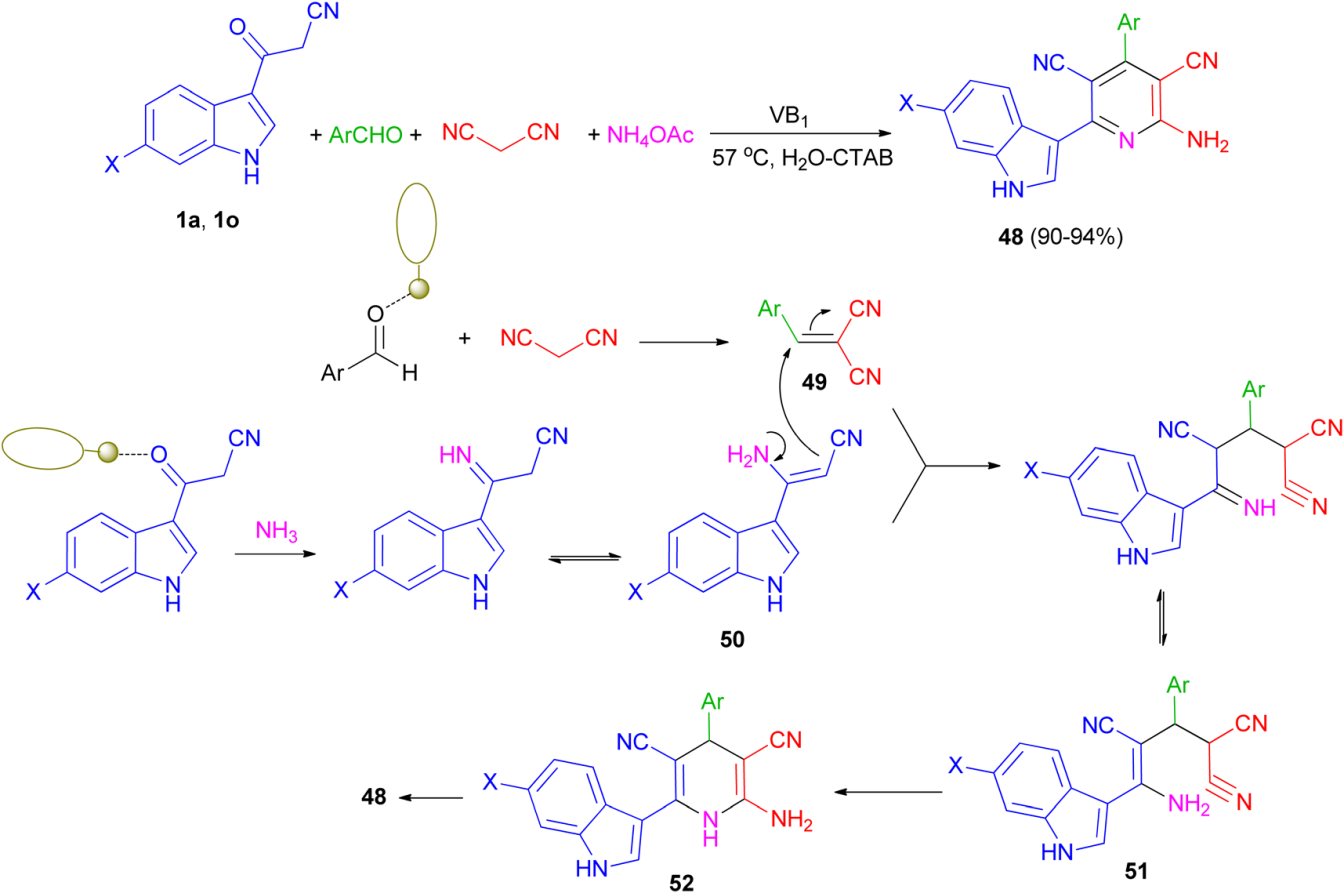
Thiamine-hydrochloride catalyzed synthesis of 2-amino-6-(1*H*-indol-3-yl)-4-arylpyridine-3,5-dicarbonitriles 48.

Singh *et al.* have developed one-pot synthesis of 2-amino-6-(1*H*-indol-3-yl)-4-arylpyridine-3,5-dicarbonitriles 48 in 90–94% yields *via* four-component reaction of 3-cyanoacetyl indoles, aromatic aldehydes, ammonium acetate, and malononitrile in aqueous micellar conditions in the presence of VB_1_ (5 mol%), and cetyltrimethylammonium bromide (CTAB) (10 mol%) at 57 °C. From the mechanistic point of view, it is proposed (Scheme 20) that first step is the formation of the intermediate 49 from the Knoevenagel condensation between aldehyde and malononitrile. Simultaneously 1 reacts with NH_3_ generated *in situ* by decomposition of ammonium acetate to give intermediate 50. Both 49 and 50 further react to give intermediate 51, which on cyclization and dehydration give the product 48*via* intermediate 52. The role of VB_1_ as a catalyst may be postulated in terms of the NH-proton of the VB_1_, leading to its interaction with the carbonyl oxygen atom of aldehyde as well as (3-cyanoacetyl)-indole, thereby facilitating the polarization and promoting the cyclocondensation reaction.^54^

**Scheme 21:**
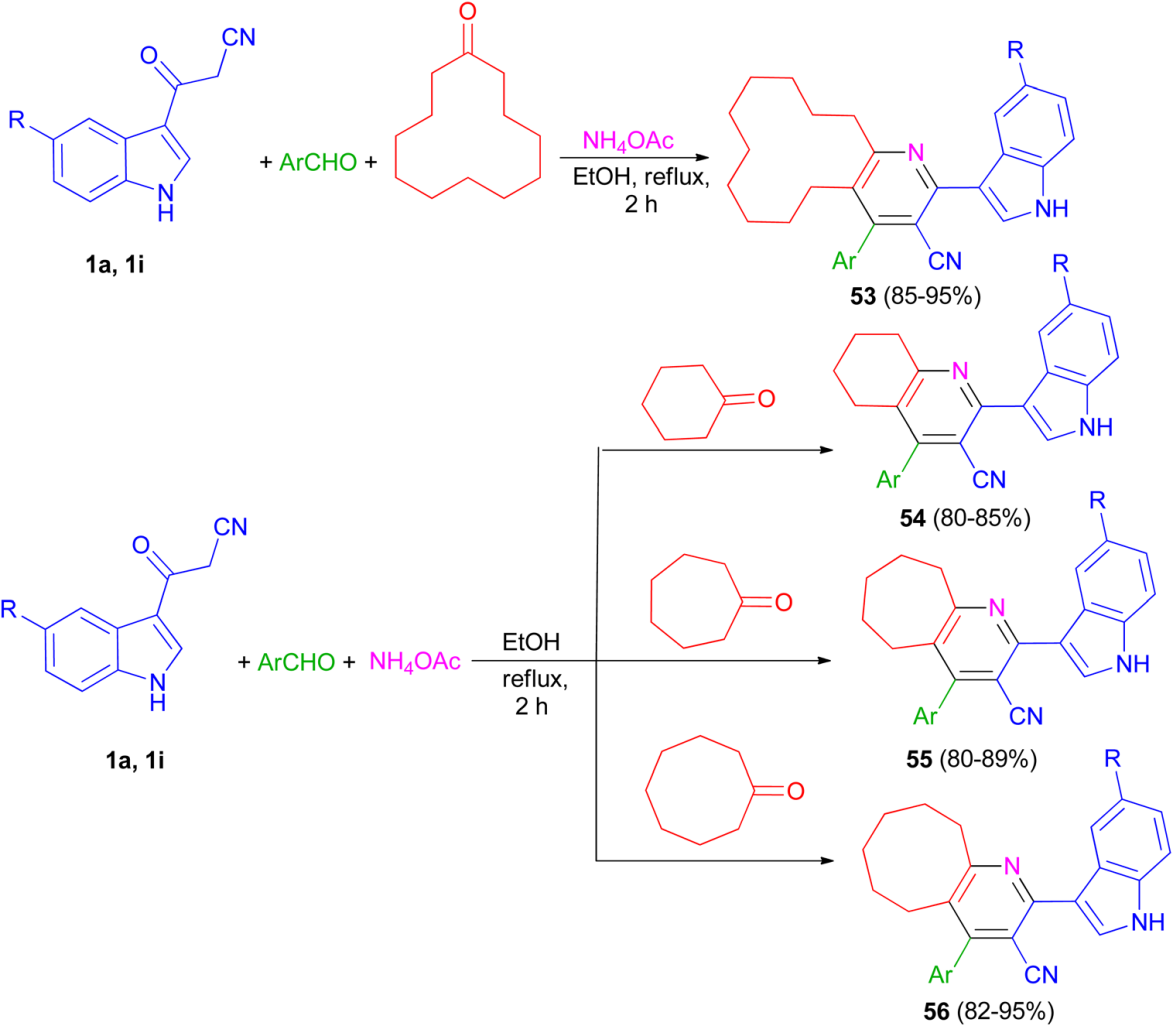
Synthesis of indole-cycloalkyl[*b*]pyridine hybrids 53–56.

The one-pot four-component reaction of 3-(1*H*-indol-3-yl)-3-oxopropanenitriles 1, aromatic aldehydes, cycloalkanones and ammonium acetate reported *via* a six-step tandem Knoevenagel condensation-nucleophilic addition to carbonyl-Michael addition-*N*-cyclization-elimination-air oxidation sequence to afford structurally intriguing indole–cycloalkyl[*b*]pyridine-3-carbonitrile hybrid heterocycles 53–56 in 80–95% yields in refluxing EtOH for 2 h (Scheme 21).^55^

**Scheme 22:**
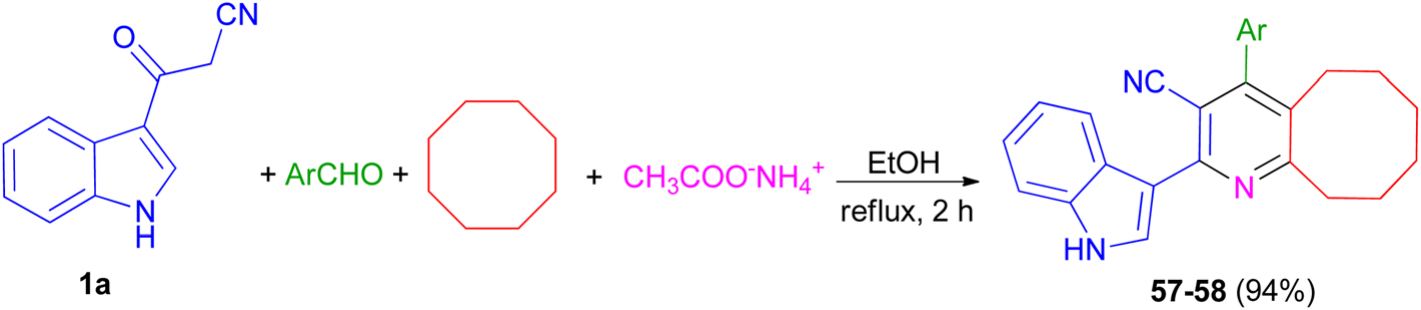
Synthesis of pyridine derivatives 57 and 58.

Priya and co-workers reported synthesis of 4-(4-fluorophenyl)-2-(1*H*-indol-3-yl)-5,6,7,8,9,10-hexahydrocycloocta[*b*]pyridine-3-carbonitrile (57) in 94% yield and 2-(1*H*-indol-3-yl)-4-(thiophen-2-yl)-5,6,7,8,9,10-hexahydrocycloocta[*b*]pyridine-3-carbonitrile (58) in 94% yield by one-pot four-component reaction of 1a, aromatic aldehydes (4-fluorobenzaldehydealdehyde, thiophene-2-carboxaldehyde), cyclooctane and ammonium acetate in EtOH at reflux for 2 h (Scheme 22). Crystal structure, Hirshfeld surface analysis, DFT calculations and molecular docking studies on pyridine derivatives as potential inhibitors of nicotinamide phosphoribosyltransferase (NAMPT) of the synthesized compounds were investigated.^56^

**Scheme 23:**
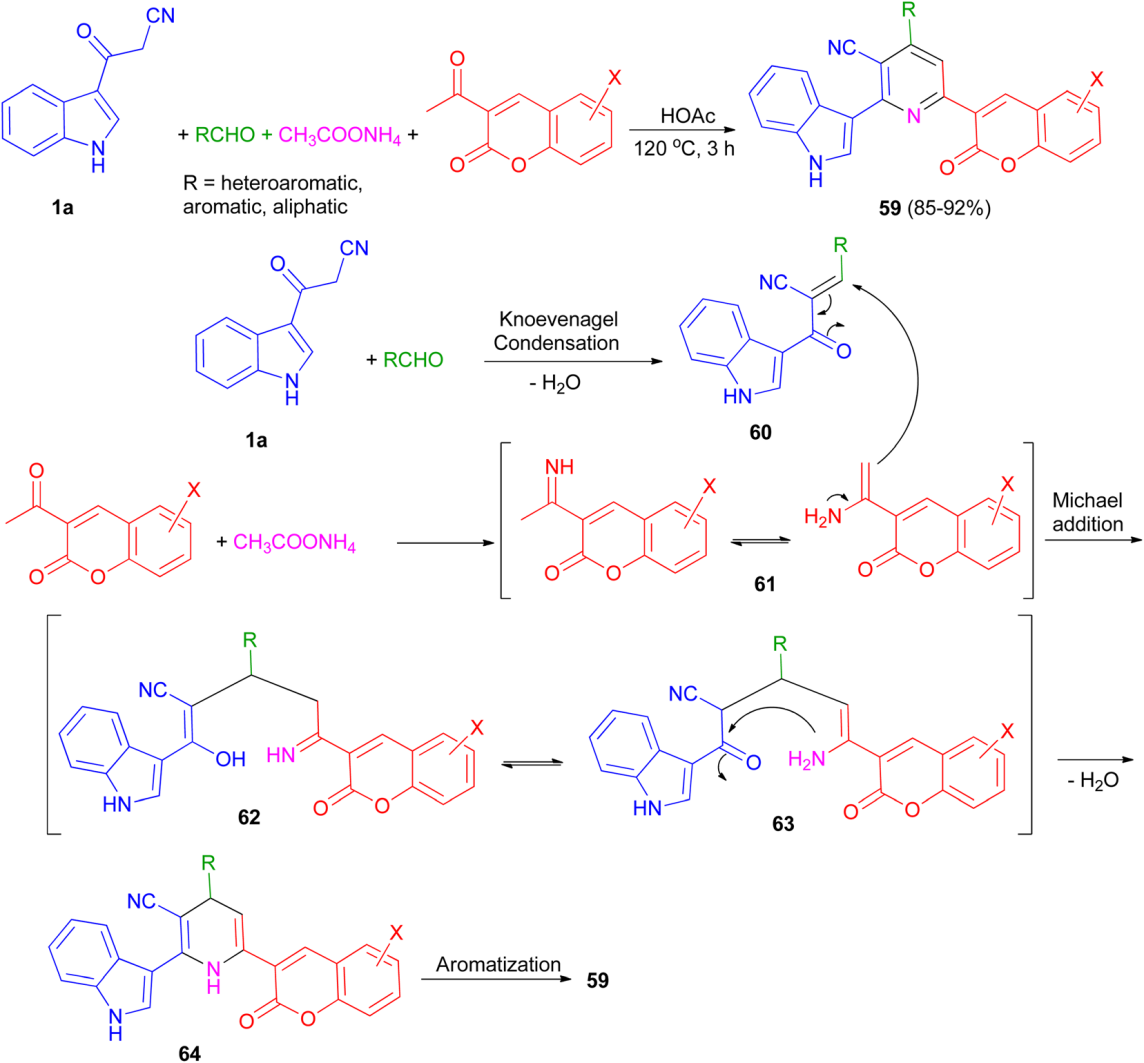
Synthesis of indole and coumarin containing pyridine-3-carbonitriles 59.

An efficient and simple procedure for the synthesis of a class of diversely functionalized indole and coumarin containing pyridine-3-carbonitrile derivatives 59 in 85–92% yields has been described through one-pot four-component condensation reaction of 3-(1*H*-indol-3-yl)-3-oxopropanenitrile (1a), various aldehydes, 3-acetyl-2*H*-chromenones, and ammonium acetate in acetic acid at 120 °C for 3 h. The plausible mechanism pathway depicted in Scheme 23. Initially, Knoevenagel condensation reaction between 1a and aldehyde gave intermediate 60 (acts as Michael acceptor) and 2-acetylchromenes and ammonium acetate gave intermediate 61. Then, intermediate 60 undergoes Michael addition with 61 to give the intermediate 62, which could apparently isomerizes to intermediate 63. Intramolecular N-cyclization of 63 gave the dihydro intermediate 64, which could further undergo dehydrogenation to afford the fully aromatized product 59.^57^

**Scheme 24:**
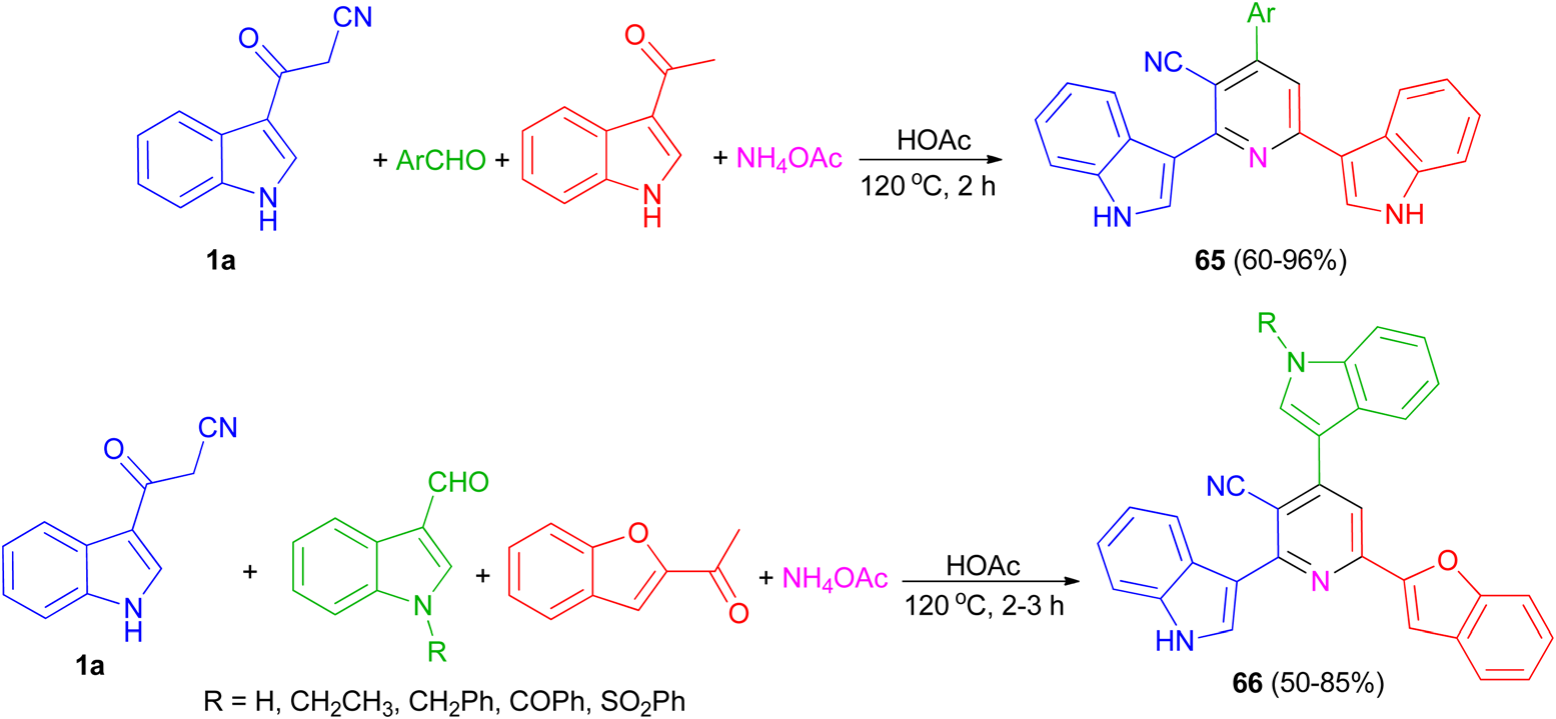
Preparation of bis(indolyl)pyridines 65 and 66.

El-Sawy and co-workers utilized the one-pot four-component condensation of 3-cyanocarbomethylindole (1a), various aldehyde, 3-acetylindole, and ammonium acetate in glacial acetic acid at 120 °C for the synthesis of 2,6-bis(1*H*-indol-3-yl)-4-(substituted-phenyl)pyridine-5-carbonitriles 65. Additionally, 2,6-bis(1*H*-indol-3-yl)-4-(benzofuran) pyridine-5-carbonitriles 66 prepared *via* a one-pot four-component condensation of 3-cyanocarbomethylindole, various *N*-substituted-indole-3-aldehydes, 2-acetylbenzofuran, and ammonium acetate in glacial acetic acid at 120 °C for 2 h (Scheme 24). The synthesized compounds exhibited *in vitro* antimicrobial (MIC, MBC), anti-biofilm properties.^58^

**Scheme 25:**
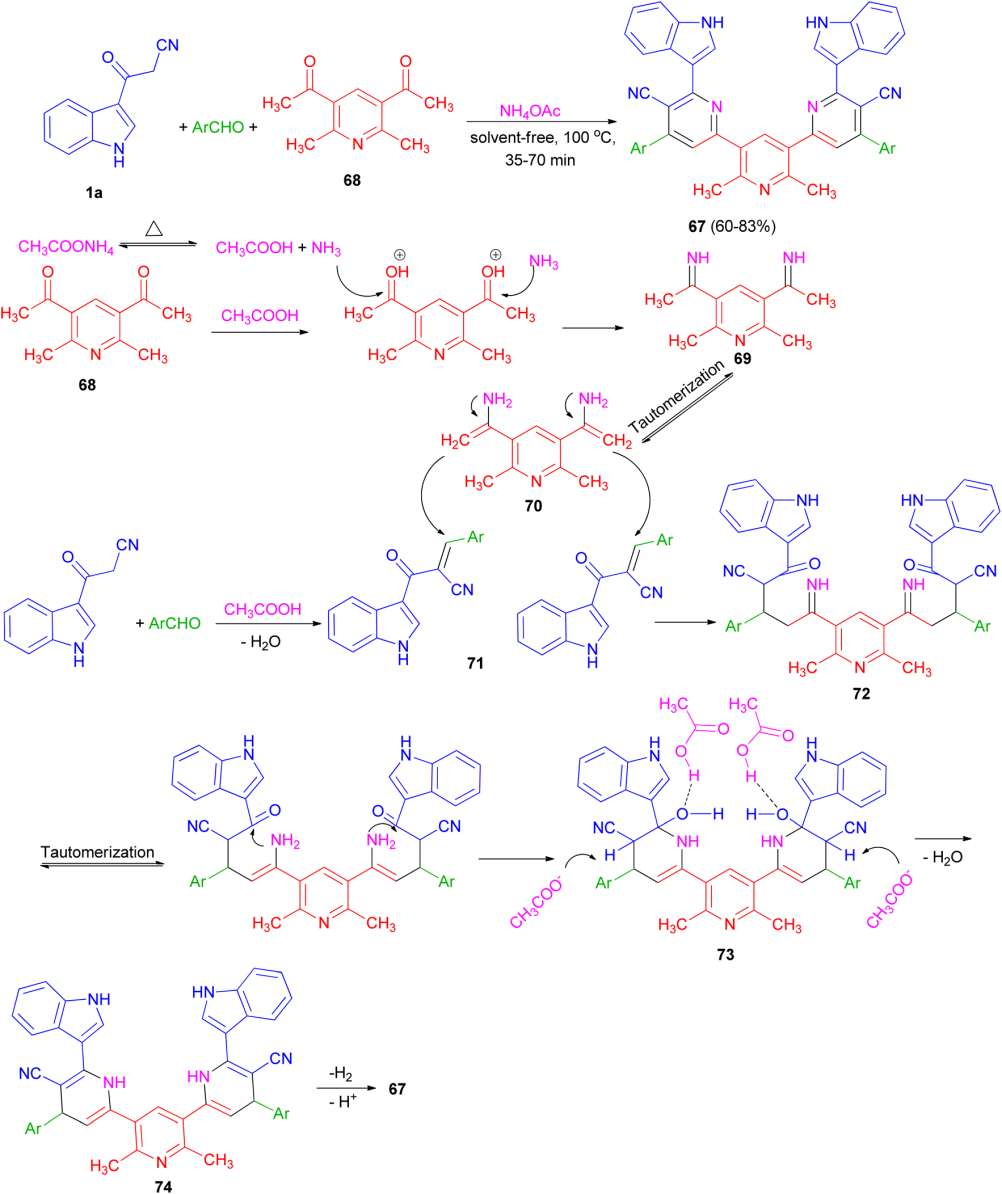
Synthesis of symmetrical terpyridines 67.

Ammonium acetate as a dual rule reagent-catalyst used for the synthesis of symmetrical terpyridines 67 in 60–83% yields by the reaction of 1,1′-(2,6-dimethylpyridine-3,5-diyl)bis(ethan-1-one) (68), 3-(1*H*-indol-3-yl)-3-oxopropanenitrile (1a) and aromatic aldehydes under solvent-free conditions at 100 °C for 35–70 min. The proposed mechanism is displayed in Scheme 25. Initially, carbonyl groups of 68 are activated by AcOH (derived from thermal dissociation of ammonium acetate) and reacted with ammonia which leads to related imine intermediate 69. Then, *via* a tautomerization process, intermediate 69 converted to intermediate 70. Meanwhile, intermediate 71 is obtained from a Knoevenagel condensation reaction between aldehyde and 1a. After that, intermediate 70 reacts with intermediate 71 which results to formation of intermediate 72. In the next step, through successive tautomerization process, and intramolecular nucleophilic attack intermediate 73 is formed. In the next step, intermediate 73 through dehydration converted to the related intermediate 74. Finally, the corresponding product 67 is produced *via* a cooperative vinylogous anomeric based oxidation mechanism both in the presence and absence of oxygen.^59^

**Scheme 26:**
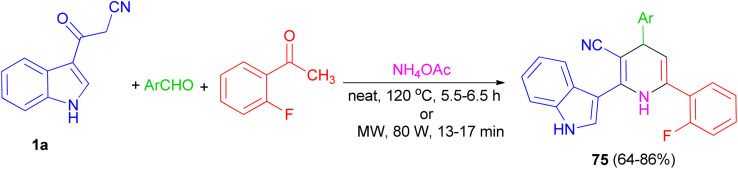
Synthesis of indole containing 1,4-dihydropyridines 75.

Further, an eco-friendly, efficient and cost effective procedure described for the synthesis of 1,4-dihydropyridine 75 in 64–86% yields by a solvent and catalyst free Hantzsch reaction's condensation of 1a, 2-fluoroacetophenone and substituted aldehyde in the presence of ammonium acetate under conventional heating at 120 °C for 5.5–6.5 h and microwave irradiation at 80 W for 13–17 min (Scheme 26). Also, anti-bacterial and anti-fungal of the synthesized compounds were investigated.^60^

### 3.3. Pyrimidine and tetrahydropyrimidine derivatives

**Scheme 27:**
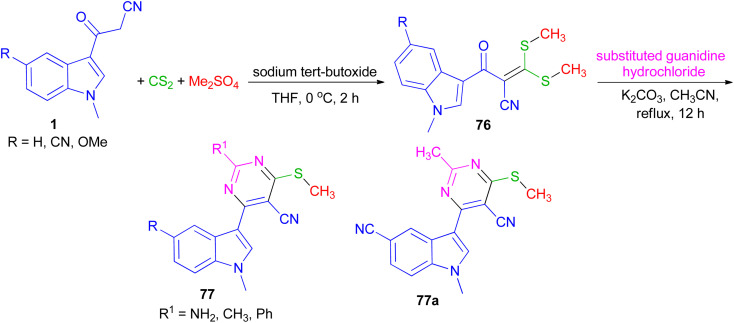
Synthesis of 4-(1-methyl-1*H*-indol-3-yl)-6-(methylthio) pyrimidine-5-carbonitriles 77.

In 2022, Patil *et al.* converted 3-(1-methyl-1*H*-indol-3-yl)-3-oxopropanenitriles 1 to 2-(1-methyl-1*H*-indole-3-carbonyl)-3,3-bis(methylthio)acrylonitriles 76 by the reaction of carbon disulfide in the presence of sodium tert-butoxide followed by alkylation with dimethyl sulfate in dry THF at 0 °C for 2 h. Further, 76 on cycloaddition with substituted guanidine hydrochloride under an alkaline condition in refluxing acetonitrile for 12 h furnished desired 4-(1-methyl-1*H*-indol-3-yl)-6-(methylthio) pyrimidine-5-carbonitriles 77 in 90–96% yields. All the synthesized compounds exhibited potent anticancer activity against breast cancer cell line, which was significantly altered with the substitution of indole and pyrimidine. In addition, compound 77a was found to be an effective anti-inflammatory agent (Scheme 27).^61^

**Scheme 28:**
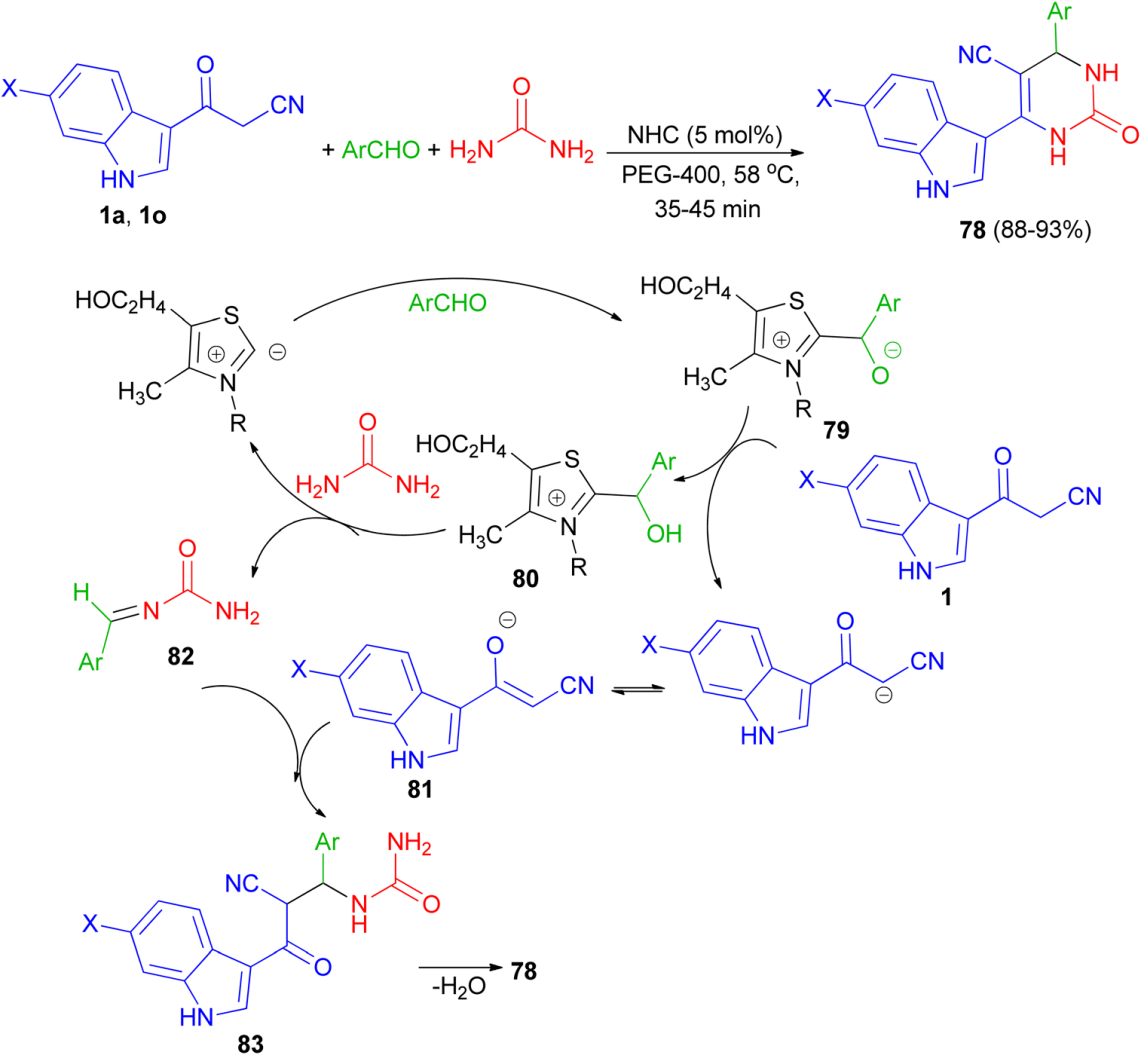
NHC catalyzed synthesis of 6-(1*H*-indol-3-yl)-2-oxo-4-aryl-1,2,3,4-tetrahydropyrimidine-5-carbonitriles 78.

In 2013, Singh *et al.* developed a three-component, one-pot, direct and highly efficient cyclocondensation method for the synthesis of 6-(1*H*-indol-3-yl)-2-oxo-4-aryl-1,2,3,4-tetrahydropyrimidine-5-carbonitriles 78 in 88–93% yields by combining an aryl aldehyde with 3-(cyanoacetyl)-indoles 1 and urea in the presence of the PEG-400 and a catalytic amount of thiazolium anion (NHC) at 58 °C for 35–45 min. The plausible mechanistic pathway for the synthesis of product 78 is illustrated in Scheme 28. The aldehyde initially reacts with NHC (as thiazolium ion) to give an intermediate 79, which further reacts with cyanoacetylindole 1 to give species 80 and corresponding anion of 1, *i.e.*, 81 by removal of acidic hydrogen. Species 80 further reacts with urea to give intermediate 82. Intermediate 81 and 82 further reacts to give intermediate 83, which upon intramolecular cyclocondensation reaction yields the desired product of series 78.^62^

**Scheme 29:**
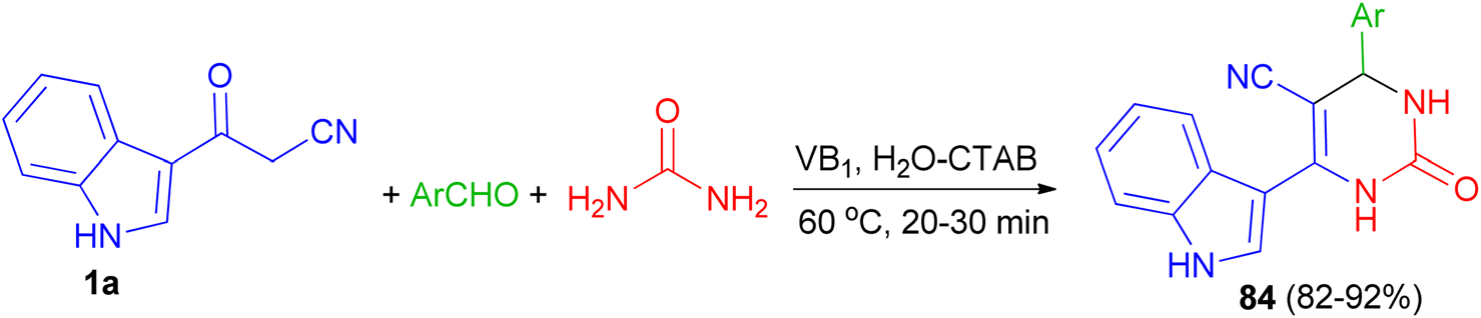
Preparation of 6-(1*H*-indol-3-yl)-2-oxo-4-aryl-1,2,3,4-tetrahydropyrimidine-5-carbonitriles 84.

Singh *et al.* employed thiamine hydrochloride (5 mol%) as catalyst for the preparation of 6-(1*H*-indol-3-yl)-2-oxo-4-aryl-1,2,3,4-tetrahydropyrimidine-5-carbonitriles 84 in 82–92% yields *via* the one-pot three-component reaction of 1a, aryl aldehydes and urea in the presence of the cationic surfactant (CTAB, 20 mol%) in water at 60 °C for 20–30 min (Scheme 29).^63^

### 3.4. Pyrazole derivatives

**Scheme 30:**
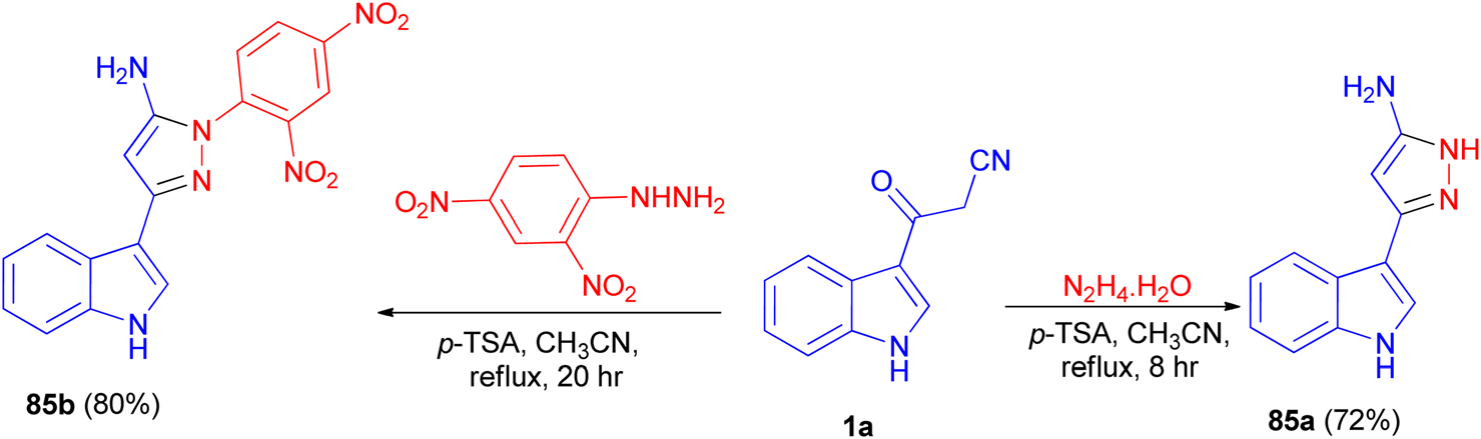
Synthesis of 5-(1*H*-indol-3-yl)-pyrazolyl derivatives 85a–b.

In 2013, Ghosh *et al.* described synthesis of 5-(1*H*-indol-3-yl)-pyrazolyl derivatives 85a–b as colorimetric sensor for anions in 72–80% yields by the reaction of 1a with hydrazine hydrate in the presence of *p*-TSA as catalyst in CH_3_CN at 82 °C for 8–20 h under nitrogen atmosphere (Scheme 30). Compound 85a as colorimetric sensor shows a drastic change in absorption spectrum and colour upon addition of F^−^ in DMSO solution due to the deprotonation of indole-NH proton. Moreover, 85b binds with F^−^, CN^−^, H_2_PO_4_^−^, AcO^−^and PhCOO^−^ ions exploiting hydrogen-bonding interaction with the shifting of absorption band to longer wavelength and subsequent colour change of the solution.^64^

**Scheme 31:**
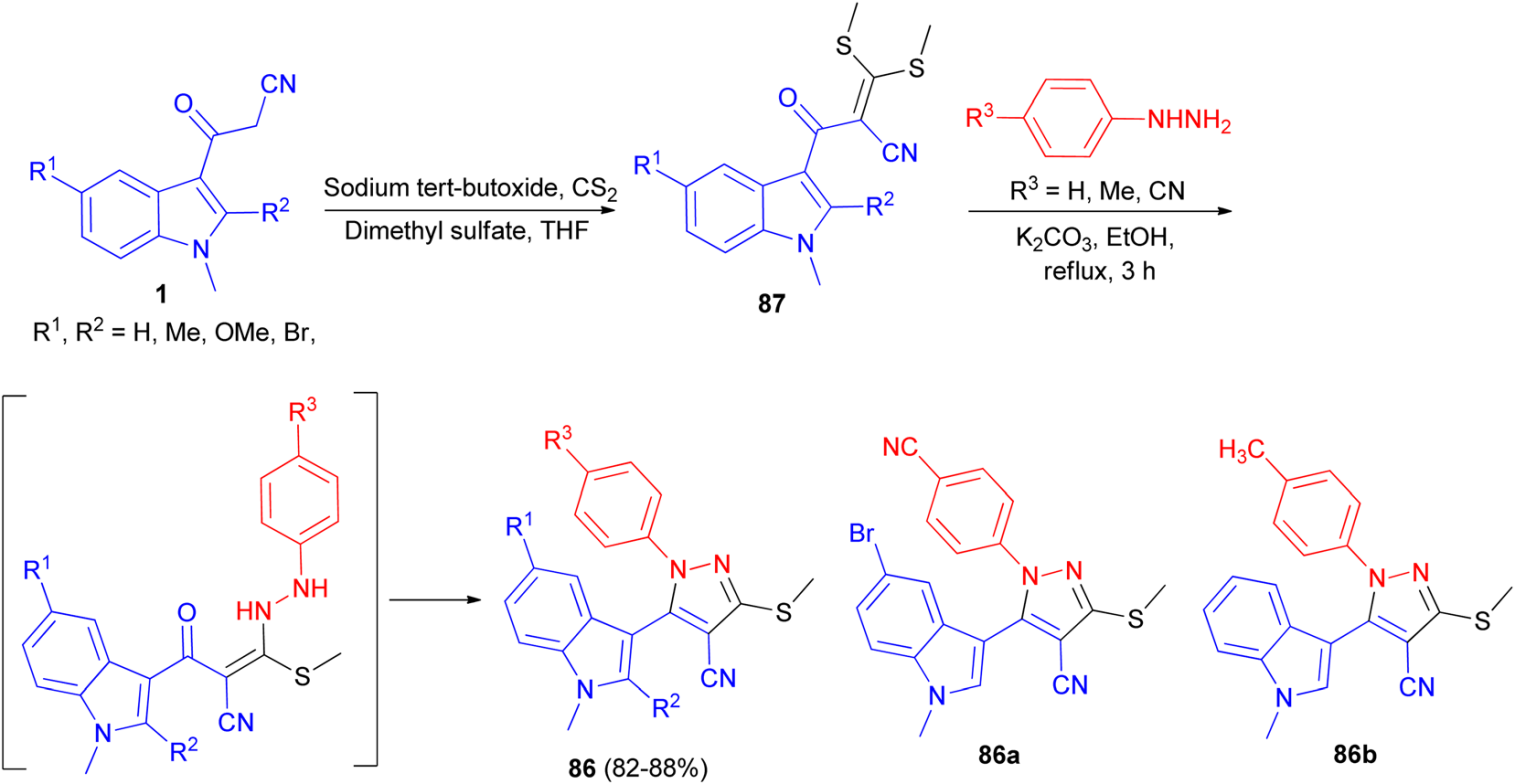
Synthesis of a series of 1,3,4,5-tetrasubstituted pyrazole derivatives 86.

Ketene dithioacetal mediated chemo- and regioselective synthesis of a series of 1,3,4,5-tetrasubstituted pyrazole derivatives 86 in 82–88% yields integrated with a bioactive indole nucleus was achieved by reacting substituted 2-(1-methyl-1*H*-indole-3-carbonyl)-3,3-bis-(methylthio)-acrylonitrile 87 and substituted phenyl hydrazine hydrochloride in the presence of a catalytic amount of anhydrous K_2_CO_3_ in EtOH under reflux conditions for 3 h. All the synthesized compounds were *in vitro* evaluated for their anti-inflammatory, antioxidant and cytotoxic potential against breast carcinoma (MCF-7). Among the compounds under investigation, 5-(5-bromo-1-methyl-1*H*-indol-3-yl)-1-(4-cyano-phenyl)-3-methylsulfanyl-1*H*-pyrazole-4-carbonitrile (86a) and 5-(1,2-dimethyl-1*H*-indol-3-yl)-3-methylsulfanyl-1-phenyl-1*H*-pyrazole-4-carbonitrile (86b) exhibited significant antitumor and anti-inflammatory activities, respectively (Scheme 31).^65^

### 3.5. Pyrazolopyridine derivatives

**Scheme 32:**
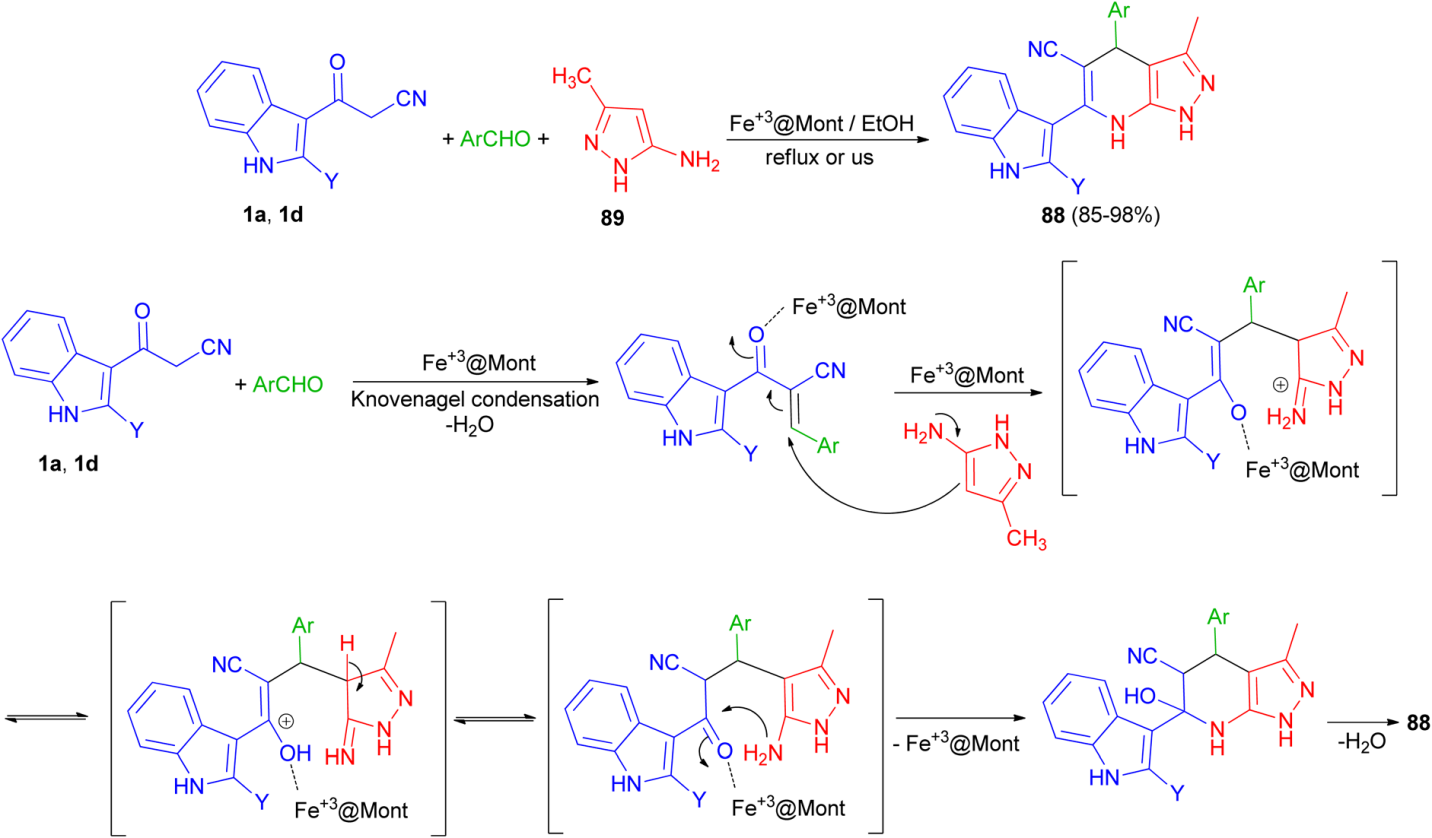
Polyfunctional pyrazolo[3,4-*b*]pyridines 88.

In 2013, Mamaghani and his group synthesized polyfunctional pyrazolo[3,4-*b*]pyridines 88*via* a regioselective one-pot three-component reaction of 3-(cyanoacetyl)indoles 1, 5-amino-3-methylpyrazole (89) and aryl aldehydes in 85–98% yields using Fe^3+^-montmorillonite as a reusable catalyst under conventional conditions in refluxing EtOH for 20–40 min and ultrasonic irradiation (40 kHz, 60 °C and 3–7 min). The plausible mechanism for the formation of 88 is outlined in Scheme 32. The formation of these products can be visualised by initial Knoevenagel condensation of aldehyde and 3-cyanoacetylindoles (1). The Fe^3+^@Mont. activated arylidene intermediate, seems to be a good acceptor for the Michael addition of 1*via* attack of the nucleophilic C-4 of the pyrazole, followed by cyclisation and loss of H_2_O to furnish the desired pyrazolopyridines 88. These products were also evaluated for their antibacterial activities. Most of the compounds exhibited excellent antibacterial activity against both Gram-negative and Gram-positive bacteria.^66^

**Scheme 33:**
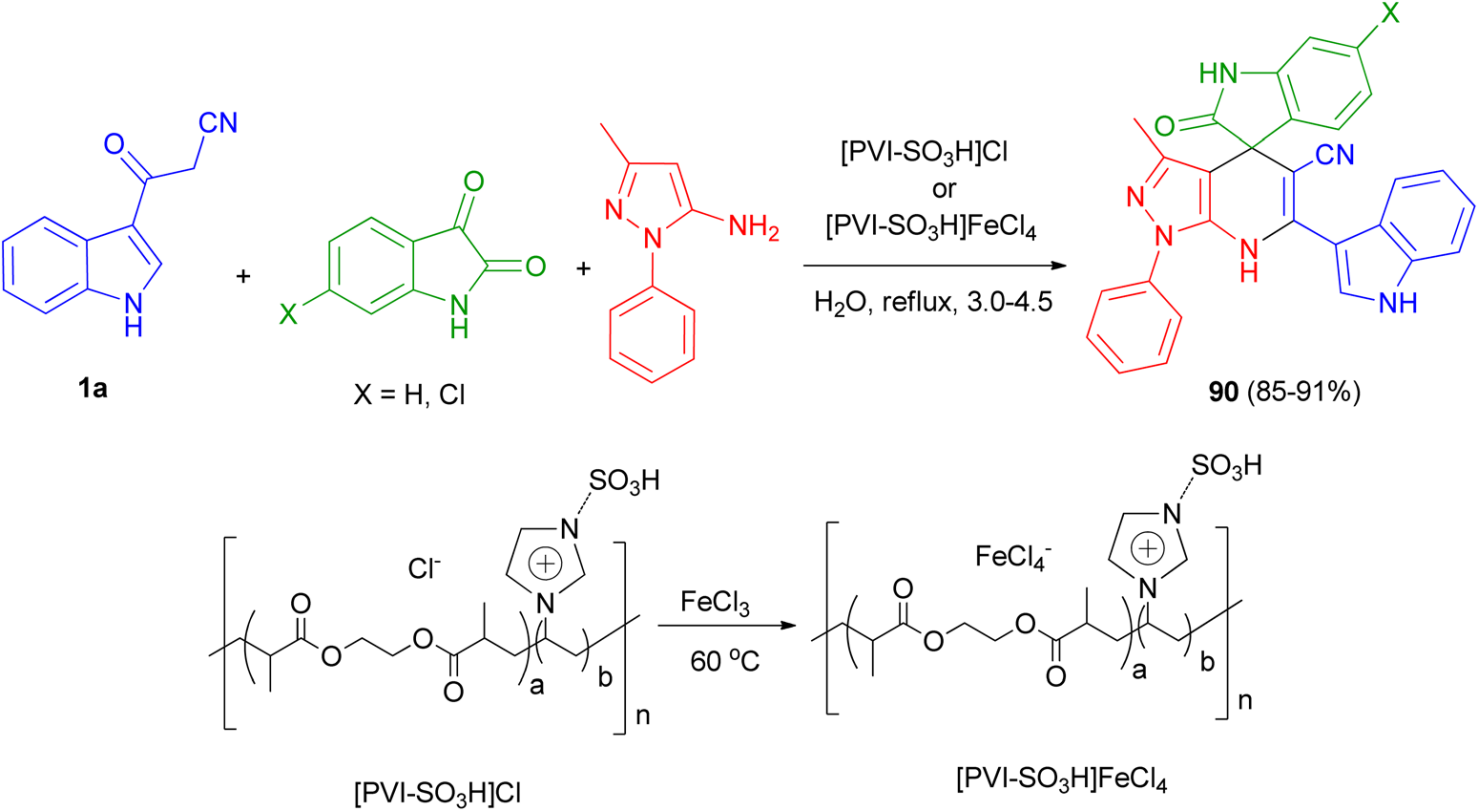
Preparation of *N*-heterocycle spiropyrans 90.

Mesoporous cross-linked poly(vinyl imidazole)s with sulfonic acid tags, [PVI-SO_3_H]Cl and [PVI-SO_3_H]FeCl_4_, were successfully applied as reusable and efficient catalysts for the preparation of N-heterocycle spiropyrans 90*via* the one-pot reaction of isatin derivatives, 3-methyl-1-phenyl-1*H*-pyrazol-5-amine and 3-(1*H*-indol-3-yl)-3-oxopropanenitrile (1a) in water under reflux conditions 3.0–4.5 h (Scheme 33). In the proposed mechanism, initially, the acidic groups (SO_3_H) of [PVI-SO_3_H]FeCl_4_ or [PVI-SO_3_H]Cl activate the carbonyl group of isatin, and 1a are enolized. Then, the reaction of 1a with isatin leads to a removal of one molecule of H_2_O to give first intermediate. In the next step, 3-methyl-1-phenyl-1*H*-pyrazol-5-amine as a nucleophile attacks to the intermediate, which acts as a Michael acceptor, to give second intermediate. Finally, the cyclocondensation reaction of second intermediate affords third intermediate, which is converted into the corresponding N-heterocycle spiropyran derivatives 90.^67^

**Scheme 34:**
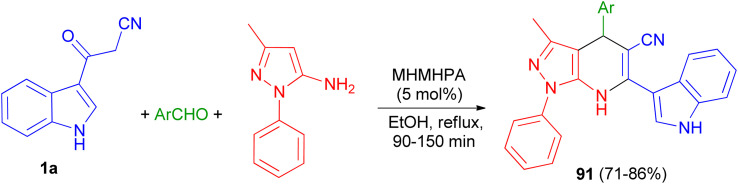
Synthesis of (3′-indolyl)pyrazolo[3,4-*b*]pyridines 91.

In 2020, Zolfigol and his group described preparation of (3′-indolyl)pyrazolo[3,4-*b*]pyridines 91 in 71–86% yields *via* an one-pot reaction between cyanoacetylindole, 3-methyl-1-phenyl-1*H*-pyrazol-5-amine, and an aromatic aldehyde in the presence of melamine hexakis(methylene)hexakis(phosphonic acid) (MHMHPA) as a heterogeneous nanocatalyst under refluxing ethanol for 90–150 min (Scheme 34).^68^

**Scheme 35:**
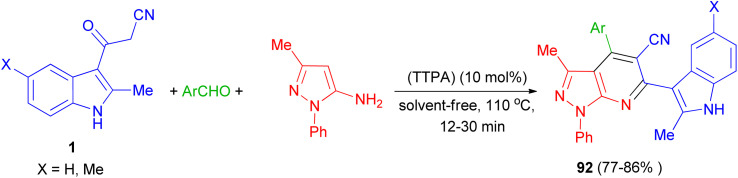
Synthesis of (3′-indolyl)pyrazolo[3,4-*b*]pyridine derivatives 92.

Zolfigol and his group synthesized (3′-indolyl)pyrazolo[3,4-*b*]pyridine derivatives 92 in 77–86% yields by the reaction of 3-methyl-1-phenyl-1*H*-pyrazol-5-amine, aryl aldehyde and 3-cyanoacetyl indoles 1 using the acrine tetrakis (phosphonic acid) (TTPA) (10 mol%) as nanocatalyst under solvent-free condition at 110 °C for 12–30 min. Also, bis aromatic aldehydes (terephthaldehyde and iso-terephthaldehyde) produced their corresponding bis(2-methyl-1*H*-indol-3-yl)-pyrazolo[3,4-*b*]pyridines (Scheme 35).^69^

**Scheme 36:**
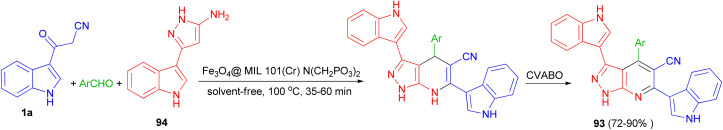
Synthesis of pyrazolo[3,4-*b*] pyridines 93.

A nano-magnetic metal–organic frameworks based on Fe_3_O_4_ namely Fe_3_O_4_@ MIL-101(Cr)–N(CH_2_PO_3_)_2_ was used as catalyst in the synthesis of pyrazolo [3,4-*b*] pyridines 93 in 72–90% yields by condensation reaction of aldehydes, 5-(1*H*-indol-3-yl)-2*H*-pyrazol-3-ylamine (94) and 3-(cyanoacetyl)indole (1a) *via* a cooperative vinylogous anomeric-based oxidation (CVABO) at 100 °C and under solvent-free conditions for 35–60 min (Scheme 36).^70^

### 3.6. Pyrazolopyrimidine derivatives

**Scheme 37:**
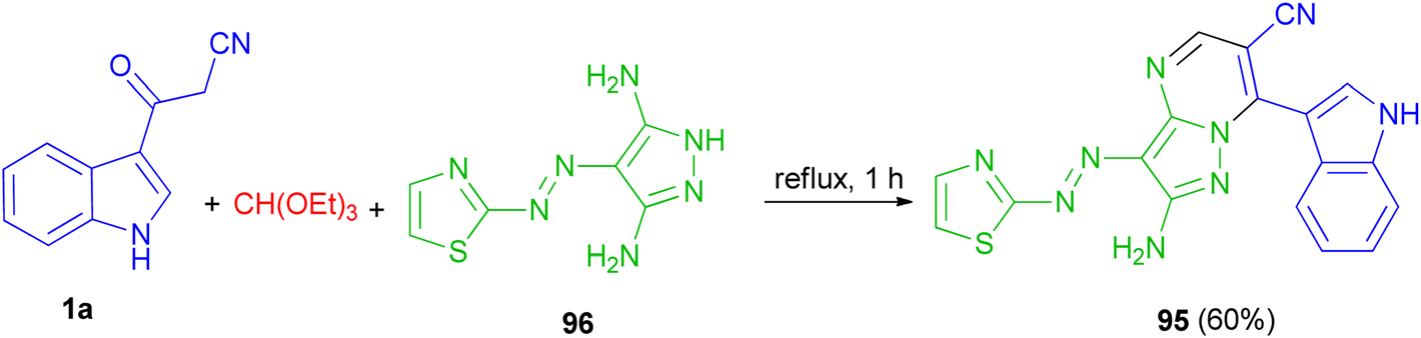
Synthesis of pyrazolo[1,5-*a*]pyrimidine derivative 95.

In 2015, El-Mekabaty and co-worker presented the synthesis of pyrazolo[1,5-*a*]pyrimidine derivative 95 in 60% yield by the reaction of 3-(1*H*-indol-3-yl)-3-oxopropanenitrile 1a with 4-(thiazol-2-yldiazenyl)-1*H*-pyrazole-3,5-diamine 96 and triethyl orthoformate under reflux conditions for 1 h (Scheme 37).^71^

**Scheme 38:**
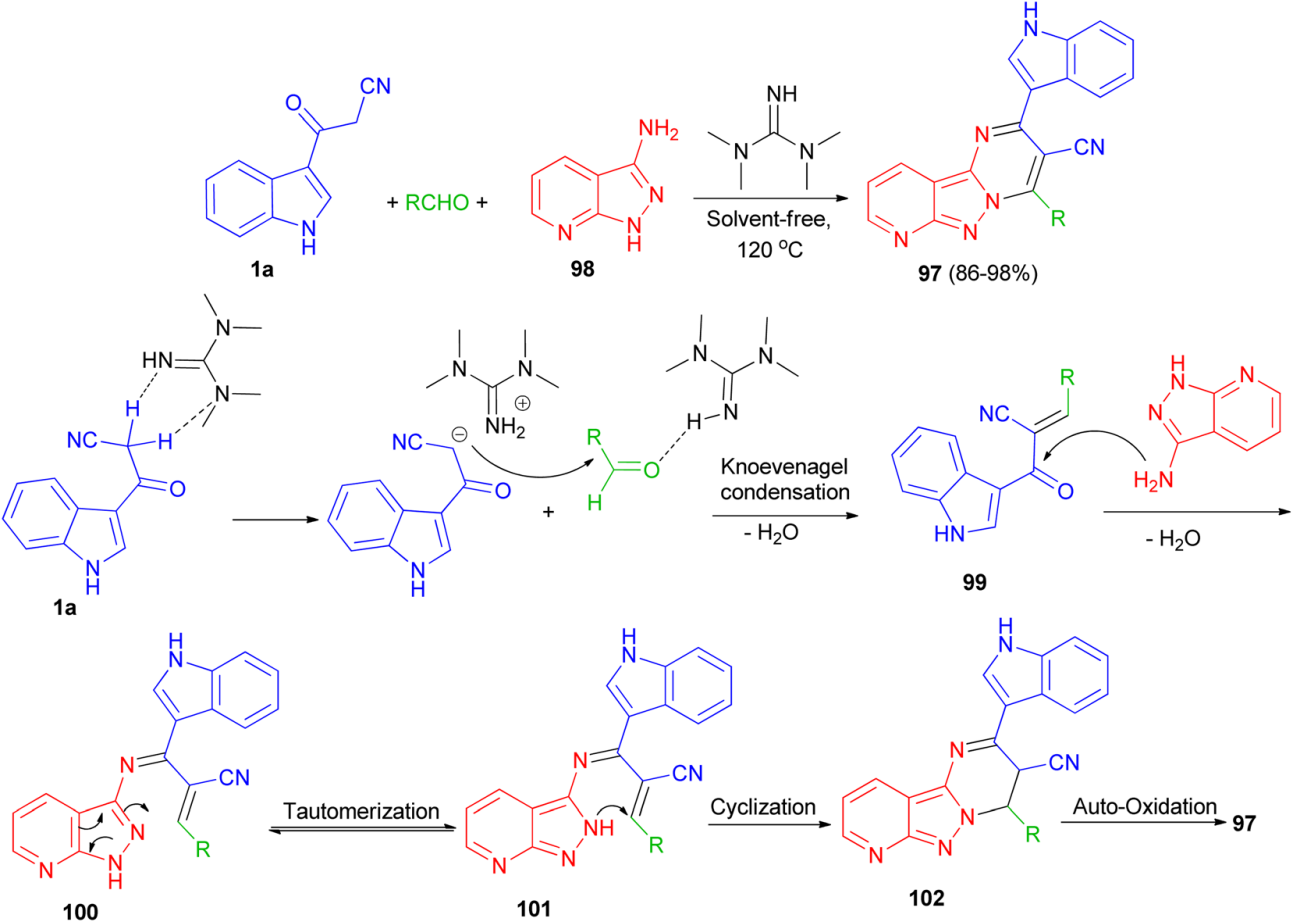
Synthesis of highly substituted pyrido[2′,3′ : 3,4]pyrazolo[1,5-*a*]pyrimidines 97 using TMG.

Jeong and co-workers described an efficient solvent-free access towards highly substituted pyrido[2′,3′ : 3,4]pyrazolo[1,5-*a*]pyrimidine-3-carbonitrile derivatives 97 in 86–98% yields through multi-component reaction of 1*H*-pyrazolo[3,4-*b*]pyridin-3-amine 98, aldehyde and 1a catalyzed by 1,1,3,3-tetramethylguanidine (TMG) at 120 °C. A plausible mechanism is proposed in Scheme 38. Initially, TMG activate aldehydes through hydrogen binding to start the nucleophilic addition of 1a, to provide a nucleophilic TMG *via* capturing a proton of 1a to form corresponding carbanions. Activation of the starting aldehydes by hydrogen bonding increases the electrophilicity of the aldehyde and assists the formation of the corresponding adduct 99 (Knoevenagel product) with 1a. This adduct 99 undergoes Michael type addition reaction with 98 to form an adduct 100 intermediate. The intermediate 100 further tautomerization *via* proton transfer N–N to give 101. After that intermediate 101 underwent intramolecular cyclization leading to the C–N bond formation and gave intermediate 102, which was followed by the auto-oxidation leading to the formation product 97.^72^

**Scheme 39:**
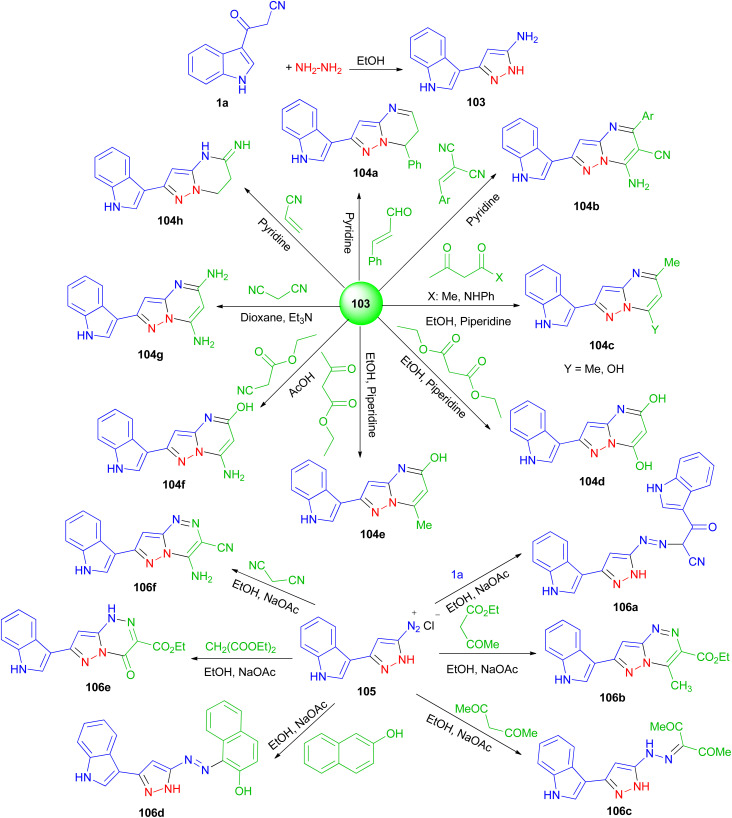
Synthesis of pyrazolo[1,5-*a*]pyrimidines 131 and pyrazolo[5,1-*c*]triazines 133.

Next, El-Mekabaty and co-workers prepared 3-(1*H*-indol-3-yl)-1*H*-pyrazol-5-amine 103 in a quantitative yield by heating 1a in dry ethanol with hydrazine hydrate, and utilized as key intermediate for the synthesis of some new pyrazolo[1,5-*a*]pyrimidines and pyrazolo[5,1-*c*]triazines. The reaction of 103 with acetylacetone, acetoacetanilide, diethylmalonate, ethylacetoacetate, ethyl cyanoacetate, malononitrile, 2-(4-methoxybenzylidene)malononitrile, cinnamaldehyde or acrylonitrile, under various conditions afforded pyrazolo[1,5-*a*]pyrimidines 104a–h in 37–81% yields after 4–22 h. Also, the reaction of diazotized 105 (prepared from 103 and the appropriate quantities of conc. HCl and sodium nitrite) with malononitrile, ethyl acetoacetate, diethylmalonate, 3-(1*H*-indol-3-yl)-3-oxopropanenitrile, β-naphthol or acetylacetone gave pyrazolo[5,1-*c*]triazines 106a–f in 44–81% yields after 6–9 h (Scheme 39). Most of the tested compounds belonging to the pyrazolo[1,5-*a*]pyrimidine series exhibited better antioxidant activities than members of the pyrazolotriazine one.^73^

### 3.7. Pyridopyrimidine derivatives

**Scheme 40:**
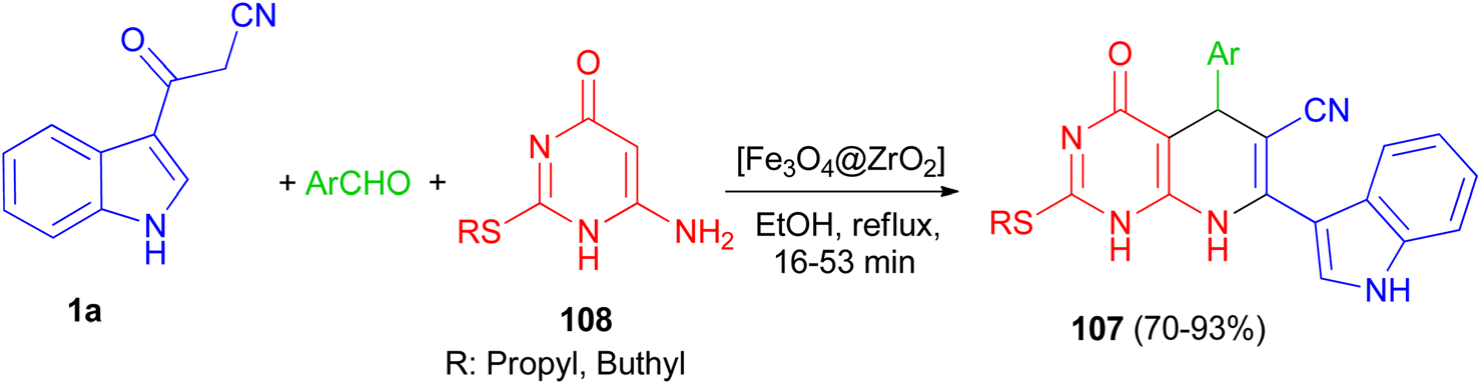
[Fe_3_O_4_@ZrO_2_] catalyzed synthesis of pyrido[2,3-*d*]pyrimidine derivatives 107.

In 2016, Mamaghani and his group established a new synthetic protocol for the preparation of pyrido[2,3-*d*]pyrimidine derivatives 107 in 70–93% yields *via* one-pot multicomponent reactions of 6-amino-2-(alkylthio)pyrimidin-4(3*H*)-one 108, 1a and arylaldehydes using [Fe_3_O_4_@ZrO_2_] as magnetically recyclable nanocatalyst in EtOH under reflux conditions for 16–53 min. The reaction proceeds *via* Knoevenagel condensation, Michael addition, cyclization and dehydration (Scheme 40).^74^

**Scheme 41:**
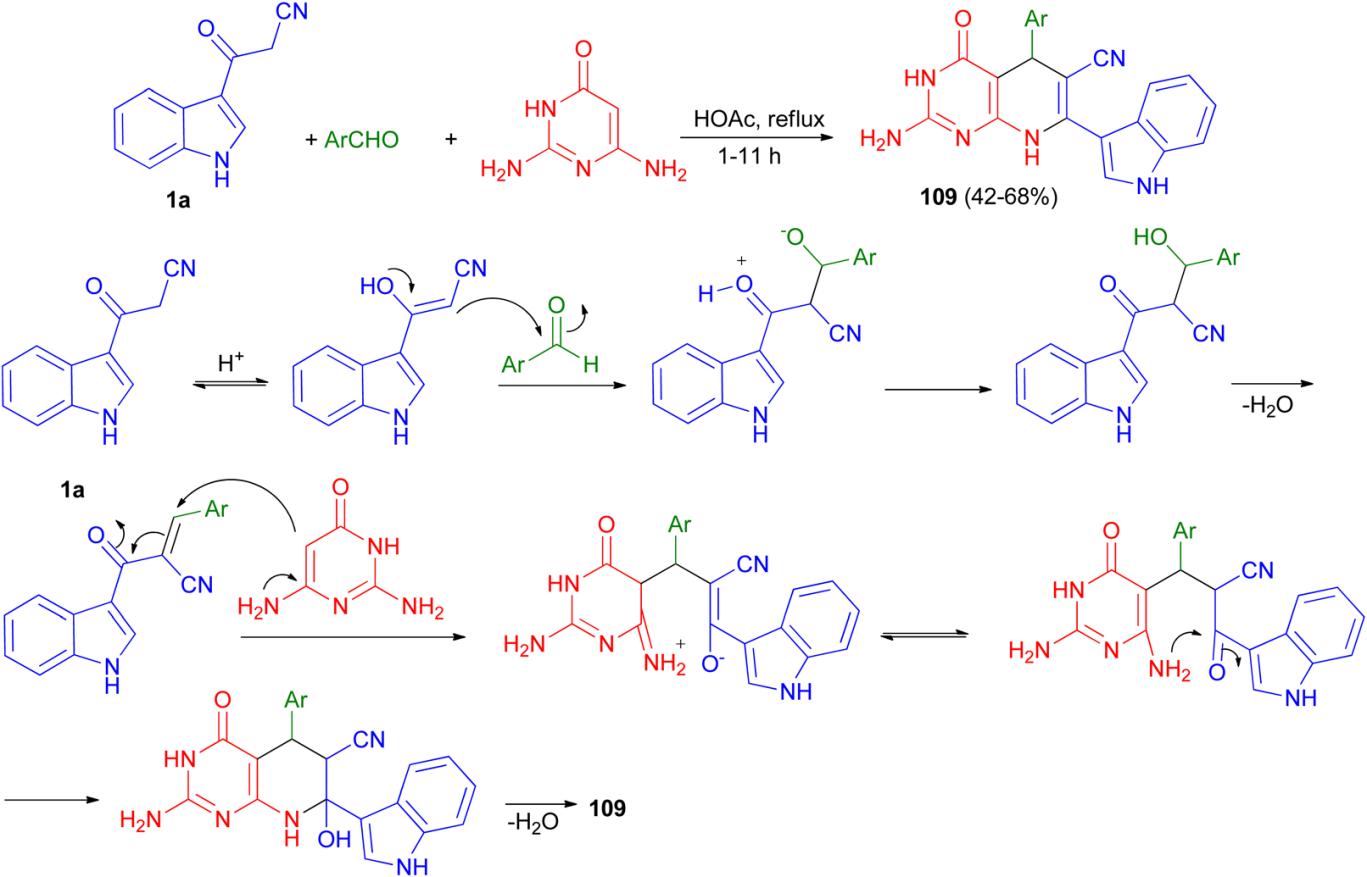
Synthesis of pyrido[2,3-*d*]pyrimidine indole substituted derivatives 109.

A series of pyrido[2,3-*d*]pyrimidine indole derivatives 109 were synthesized in 42–68% yields by one-pot three-component cyclocondensation Michael reaction between 2,6-diaminopyrimidine-4(3*H*)-one, 1a and aromatic aldehydes in boiling acetic acid as solvent for 1–11 h. A plausible mechanism is presented in Scheme 41. Initially, a conventional Knoevenagel condensation occurs which is initiated by a nucleophilic attack of the 3-(2-cyanoacetyl)indole methylene carbon atom towards the aldehyde carbonyl (C) atom. The second stage starts again *via* a nucleophilic attack, this time of the C-5 carbon atom of the aminopyrimidine towards the aryl-substituted center (former carbonyl carbon of the reactant aromatic aldehyde). An intermediate is generated which then undergoes intramolecular cyclization mediated by nucleophilic attack of the amino group of the aminopyrimidine towards the carbonyl (C) atom. Dehydration at the latter then finally leads to the desired product 109.^75^

**Scheme 42:**
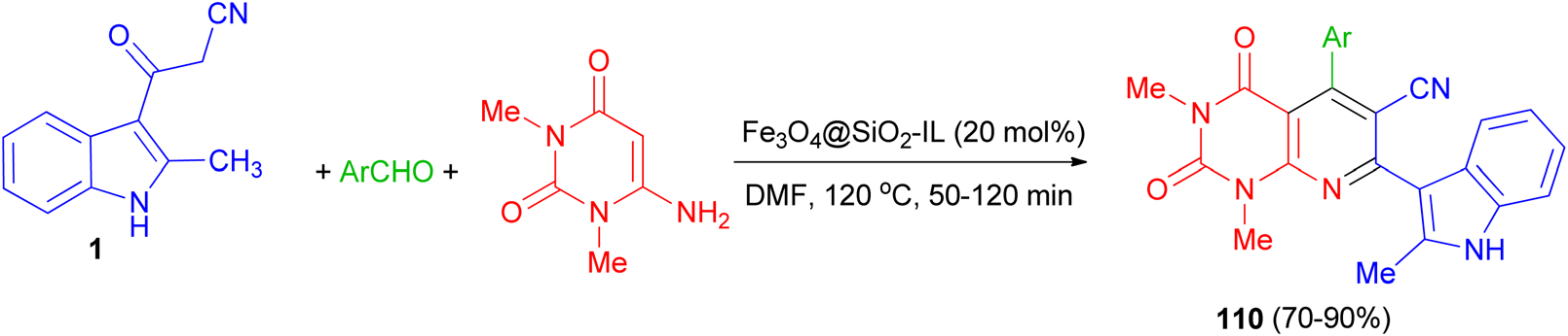
Synthesis of indole-substituted pyrido[2,3-*d*]pyrimidines 110.

After that, bioactive 2-methylindole-substituted pyrido[2,3-*d*]pyrimidine derivatives 110 in 70–90% yields were synthesized through one-pot three-component reaction of aromatic aldehydes, 6-amino-*N*,*N*-dimethyluracil, 3-(2-methyl-1*H*-indol-3-yl)-3-oxopropanenitrile (1) in the presence of Fe_3_O_4_@SiO_2_-IL nanocatalyst as an efficient and magnetically retrievable catalyst in DMF at 120 °C for 50–120 min. The antibacterial activity of synthesized compounds was also examined and most of them showed good antibacterial activity against the tested strains (Scheme 42).^76^

**Scheme 43:**
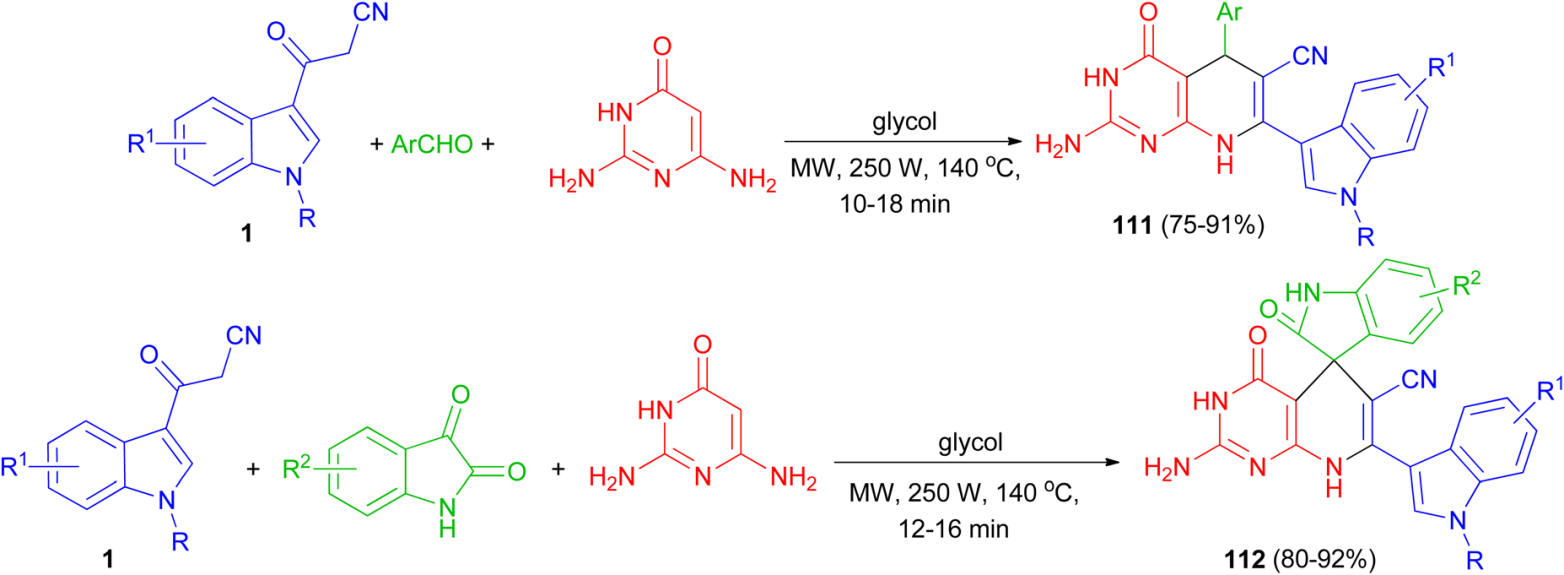
Synthesis of indolyl substituted and spirooxindole pyrido[2,3-*d*]pyrimidine derivatives 111 and 112.

Zhu and co-workers established an efficient and catalyst-free protocol for the synthesis of a series of indole substituted or spirooxindole-consisted dihydropyrido[2,3-*d*]pyrimidine derivatives 111 and 112 in 75–92% yields by one-pot three-component reaction of 2,6-diaminopyrimidine-4-one, various aryl aldehydes or isatins, and 3-cyanoacetyl indoles 1 in glycol under microwave irradiation at 250 W and 140 °C for 10–18 min (Scheme 43).^77^

### 3.8. Tetrazolopyrimidine and triazolopyrimidine derivatives

**Scheme 44:**
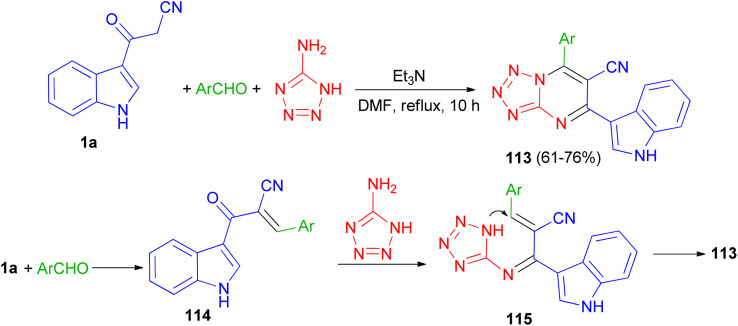
Preparation of 7-substituted-5-(1*H*-indol-3-yl)tetrazolo[1,5-*a*]pyrimidine-6-carbonitriles 113.

In 2020, a series of 7-substituted-5-(1*H*-indol-3-yl)tetrazolo[1,5-*a*]pyrimidine-6-carbonitrile 113 was synthesized in 61–76% yields *via* a one-pot, three-multicomponent reaction of appropriate aldehydes, 1*H*-tetrazole-5-amine and 3-cyanoacetyl indole (1a) using triethylamine as catalyst in DMF under reflux condition for 10 h. A probable mechanism is suggested in Scheme 44. Firstly, TEA initiates aldehydes through hydrogen binding to start the nucleophilic addition of 1a. Activation of the starting aldehydes by hydrogen bonding increases the electrophilicity of the aldehyde and supports the production of the corresponding intermediate 114 (Knoevenagel product) with compound 1a. This adduct undergoes a Michael type addition reaction with 1*H*-tetrazol-5-amine to yield an adduct 115 intermediate. After that, intermediate 115 underwent intramolecular cyclization leading to the C–N bond formation followed by the auto-oxidation leading to the formation product 113. The synthesized compounds showed potent anticancer activities against human colon and human lung cancer.^78^

**Scheme 45:**
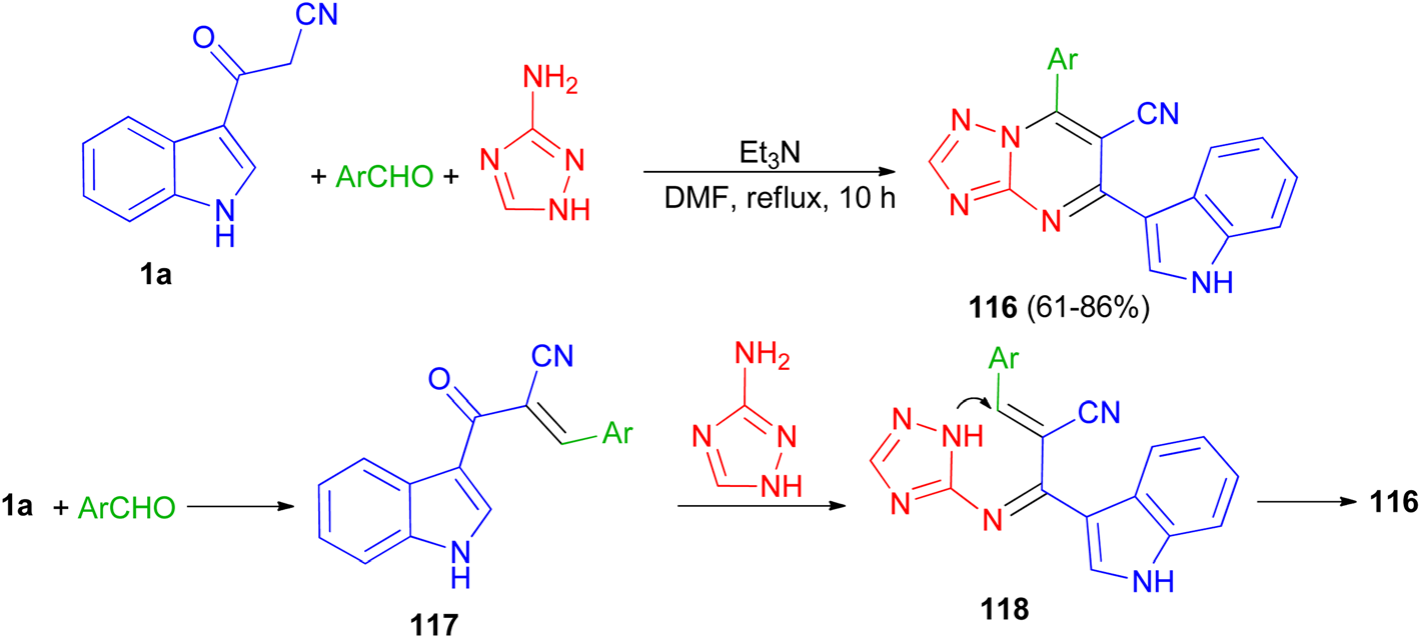
Preparation of triazolopyrimidine derivatives 116.

A series of triazolopyrimidine derivatives 116 was produced *via* three-component reactions of suitable aromatic or heteroaromatic carboxaldehyde, 3-amino-1,2,4-triazole, and 3-indolyl-3-oxopropanenitrile (1a) using triethylamine as a catalyst in DMF at 120 °C for 10 h. Antiproliferative activity of the compounds has been examined toward four different human cancer cells and one human healthy cell line. Some of synthesized compounds are active against the human colon cancer; all triazolopyrimidines are active toward MCF-7; and are effective anticancer applicants on hormone-dependent instead of hormone-independent MCF-7. A possible mechanism proposed in Scheme 45. First, triethylamine activated the nucleophilic reaction of 1a*via* hydrogen bonding to excite carboxaldehyde. The initiation of carboxaldehyde by H-bond enhances the electrophilicity of the carboxaldehyde and improves the building of a transitional 117. Intermediate 117 and 3-amino-1,2,4-triazole then undergo Michael's reaction to form intermediate 118*via* intramolecular cyclization reaction to form a C–N bond. Subsequently, compound 116 obtained in 61–86% yields by autoxidation.^79^

**Scheme 46:**
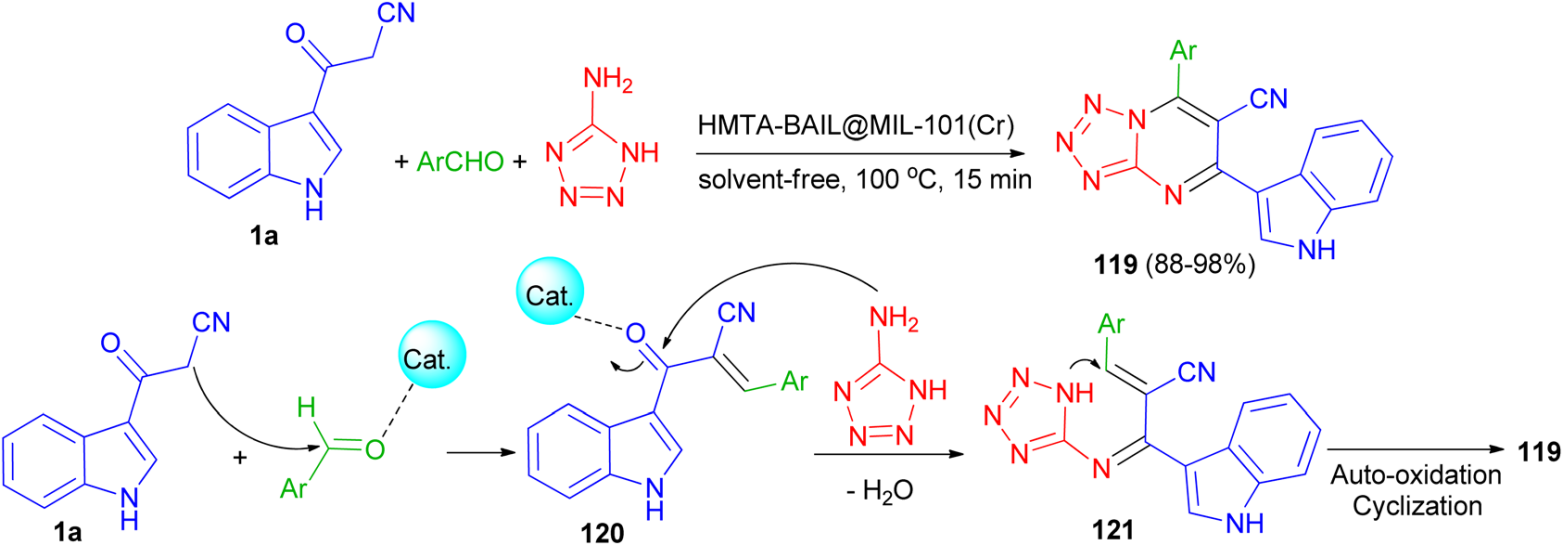
Synthesis of tetrazolo[1,5-*a*]pyrimidine-6-carbonitriles 119.

Next, Ghasemzadeh *et al.* utilized the one-pot three-component reaction of benzaldehydes, 1*H*-tetrazole-5-amine, and 3-cyanoacetyl indole (1a) in the presence of a new hexamethylenetetramine-based ionic liquid/MIL-101(Cr) metal–organic framework as a recyclable catalyst for the synthesis of tetrazolo[1,5-*a*]pyrimidine-6-carbonitriles 119 in 88–98% yields under solvent-free conditions at 100 °C for 15 min. A plausible mechanism is shown in Scheme 46. It is suggested that HMTA-BAIL@MIL-101(Cr) serves as a dual Brønsted/Lewis acid catalyst (IL/Cr^3+^ active sites), increasing the electrophilicity of the carbonyl groups of the aldehyde and the intermediates. Firstly, the activated carbonyl of the benzaldehyde undergoes a Knoevenagel condensation reaction with 3-cyanoacetyl indole to afford the intermediate 120, followed by the condensation reaction with 1*H*-tetrazole-5-amine to produce the intermediate 121. The intramolecular cyclization of the intermediate 121 with a subsequent auto-oxidation reaction finally gives the desired product 119.^80^

**Scheme 47:**
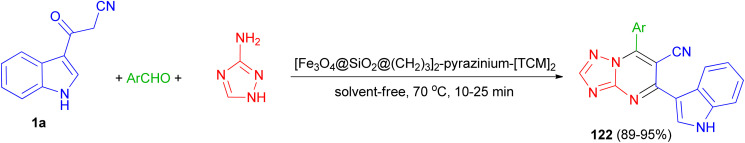
Synthesis of 7-aryl-5-(1*H*-indol-3-yl)-[1,2,4]triazolo[1,5-*a*]pyrimidine-6-carbonitriles 122.

In 2022, Baghery and his group developed three-component reaction of aromatic aldehydes with 3-(1*H*-indol-3-yl)-3-oxopropanenitrile (1a) and 1*H*-1,2,4-triazol-5-amine under the solvent-free condition at 70 °C in the presence of polyionic magnetic nanoparticles with pyrazine bridge [Fe_3_O_4_@SiO_2_@(CH_2_)_3_]_2_-pyrazinium-[TCM]_2_ as a catalyst for the synthesis of 7-aryl-5-(1*H*-indol-3-yl)-[1,2,4]triazolo[1,5-*a*]pyrimidine-6-carbonitriles 122 in 89–95% yields *via* a cooperative anomeric-based oxidation (Scheme 47).^81^

### 3.9. Furan and dihydrofuran derivatives

**Scheme 48:**
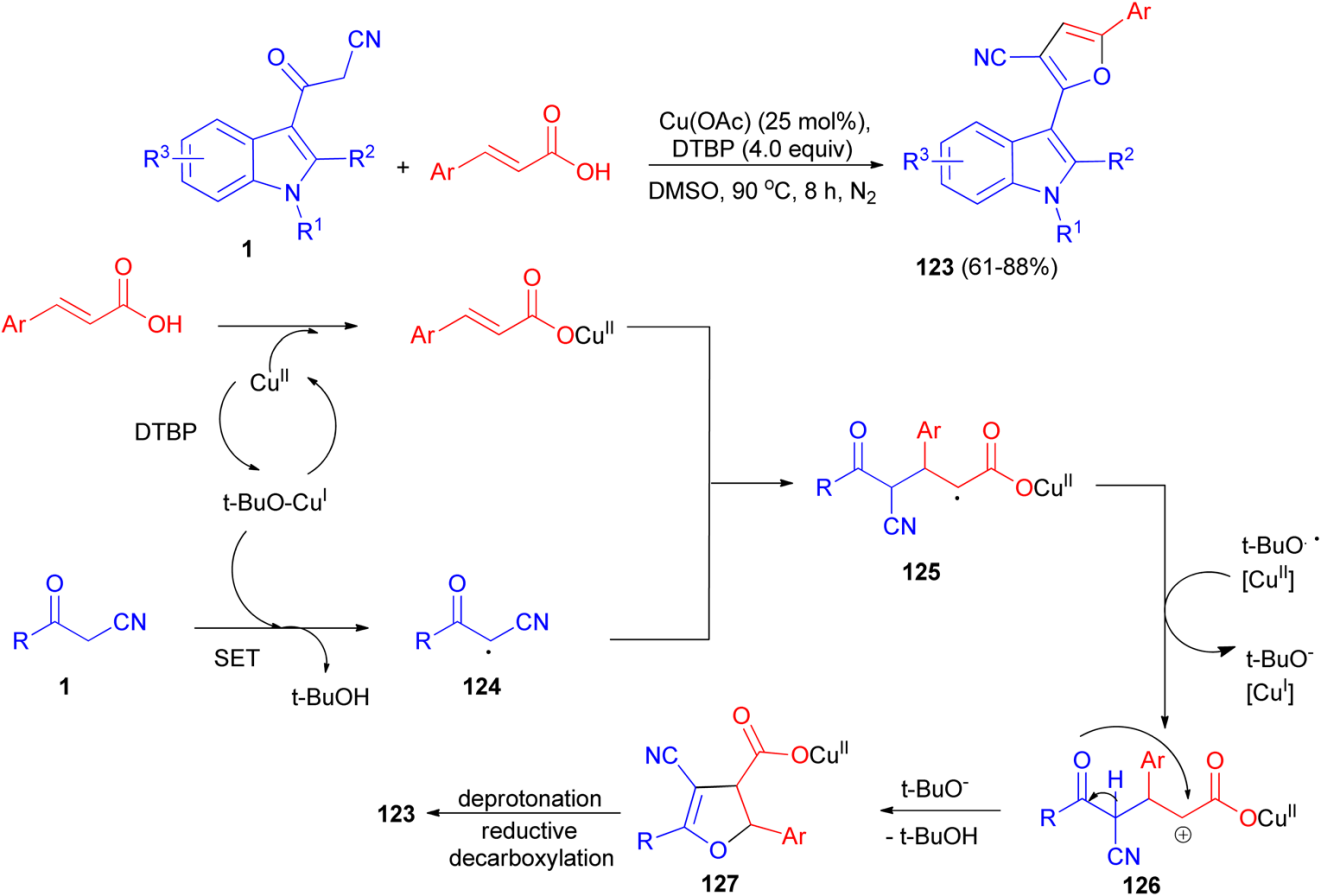
Regioselective synthesis of 3-(2-furanyl) indole derivatives 123.

Sashidhara *et al.* developed the reaction between 3-cyanoacetyl indoles and α,β-unsaturated carboxylic acids in the presence of Cu (OAc)_2_ as catalyst and di-*tert*-butyl peroxide as an external oxidant in DMSO at 90 °C. This reaction undergoes radical addition, decarboxylative processes, and provides a facile regioselective 3-(2-furanyl) indole derivatives 123 in 61–88% yields after 8 h. A plausible reaction mechanism is depicted in Scheme 48. Initially, cinnamic acid reacts with Cu(OAc)_2_ to form cupric cinnamate. Then, 3-cyanoacetyl indole reacts with *t*-BuO radical to generate the carbon center radical 124. Subsequently, the addition of radical 124 to the α-position of the double bond in cupric cinnamate would give the intermediate 125. This intermediate 125 undergoes single electron oxidation, and gets converted into intermediate 126, which on further intramolecular cyclization resulted in the formation of intermediate 127. Finally, intermediate 127 undergoes deprotonation and reductive decarboxylation to generate the product 123.^82^

**Scheme 49:**
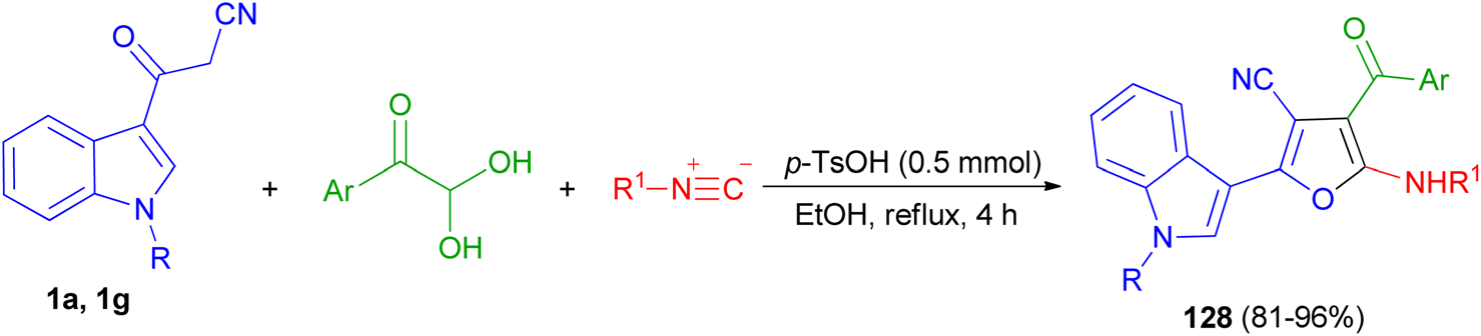
Synthesis of furan based densely substituted biheteroaryls 128.

In 2019, Jeong *et al.* developed a facile and efficient acid-catalyzed cascade reaction for the synthesis of biheteroaryl structural motifs containing densely functionalized furans in refluxing EtOH for 4 h. The reaction sequence involves a Knoevenagel condensation of arylglyoxals with 3-(1*H*-indol-3-yl)-3-oxopropanenitriles 1 and subsequently an isocyanide insertion *via* formal [4 + 1] cycloaddition followed by rapid [1,3]-H shift to afford uniquely decorated biheterocycles 128 in 81–96% yields after 4 h (Scheme 49).^83^

**Scheme 50:**
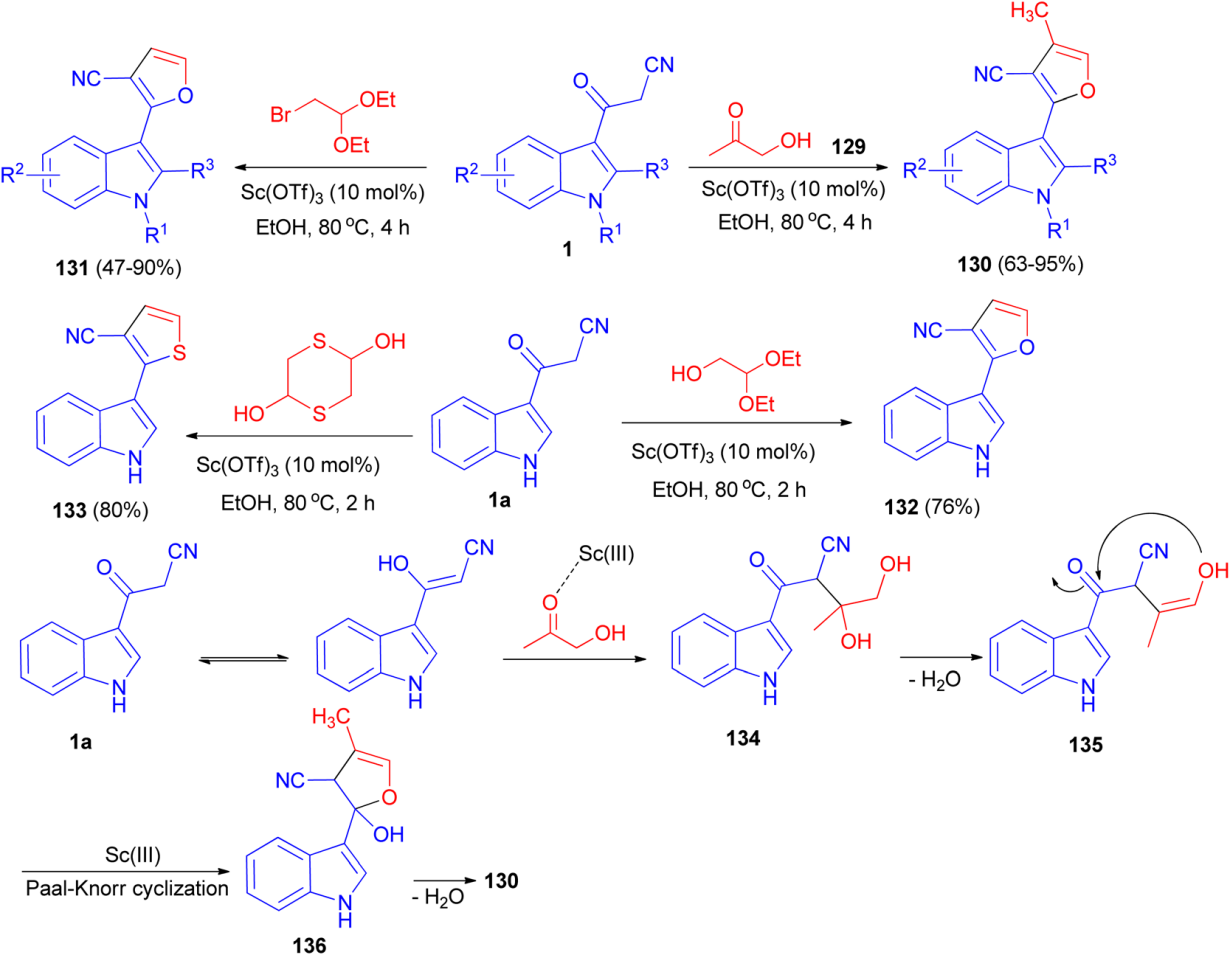
Sc(OTf)_3_-catalyzed synthesis of polysubstituted furans 130–133.

A [3 + 2] cyclization reaction of arylacetonitriles 1 and hydroxyacetone 129 was developed by using Sc(OTf)_3_ as a catalyst to synthesize some furan derivatives 130. Bifunctional C2-based acetals, such as α-bromoacetaldehyde acetal, 1,4-dithiane-2,5-diol and glycolaldehyde diethyl acetal, also reacted readily as alternative counterpart reagents to arylacetonitriles. The reactions were performed in refluxing ethanol for 2–4 h and afforded the desired products 131–133. The prepared furan compound 131a (R^1^=R^2^=R^3^=H) exhibited a highly potential fungicidal agent against *Botrytis cinerea*, *Verticillium dahliae*, *Fusarium culmorum* and *Septoria nodorum* Berk. A plausible mechanism is depicted in Scheme 50. Incipiently, the activated acetol with Sc(OTf)_3_ was triggered form the indolyl diol intermediate 134 by the enol form of 1a to, followed by dehydration to generate intermediate 135. Subsequently, the intramolecular nucleophilic addition of the hydroxy onto the keto-carbonyl would then occur, enabling the formation of an intermediate 136 with a five-membered ring system. Finally, the furan product was formed through the dehydration of intermediate 136.^84^

**Scheme 51:**
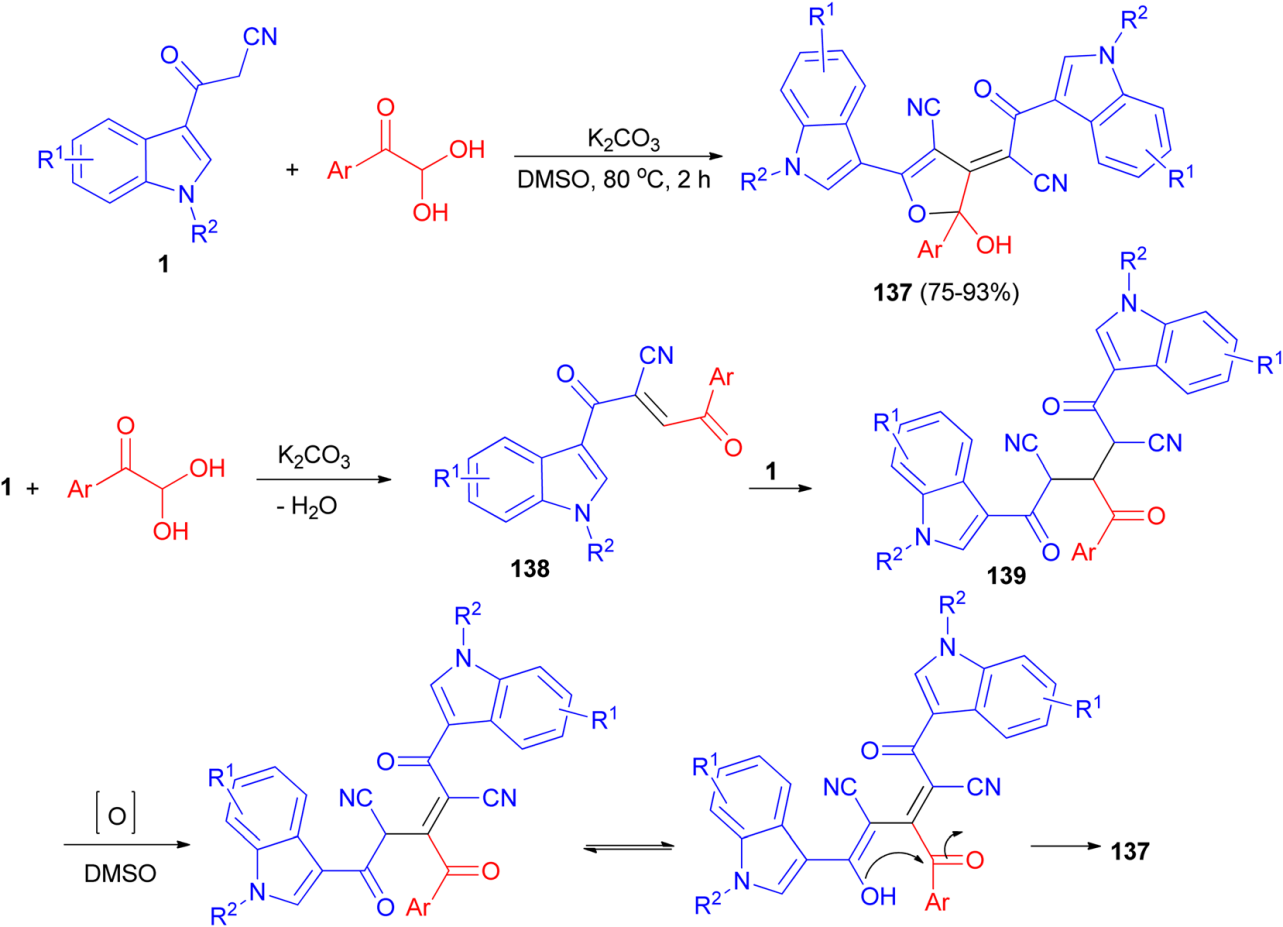
Synthesis of polyfunctional 2-hydroxy-2,3-dihydrofurans 137.

Wu *et al.* established an efficient base-promoted tandem cyclization for the synthesis of polyfunctional 2-hydroxy-2,3-dihydrofurans 137 in 75–93% yields from arylglyoxal monohydrates and 3-(1*H*-indol-3-yl)-3-oxopropanenitriles 1 in DMSO at 80 °C for 2 h. A tandem process is illustrated for the formation of 137 in Scheme 51. Initially, intermediate 138 was formed by means of a Knoevenagel condensation between phenylglyoxal monohydrate and 1. Subsequently, another amount of 1 reacted with 138 to form intermediate 139*via* a Michael addition. Finally, the intermediate 139 underwent oxidation, tautomerization, and intramolecular cyclization to form the final product 137.^85^

**Scheme 52:**
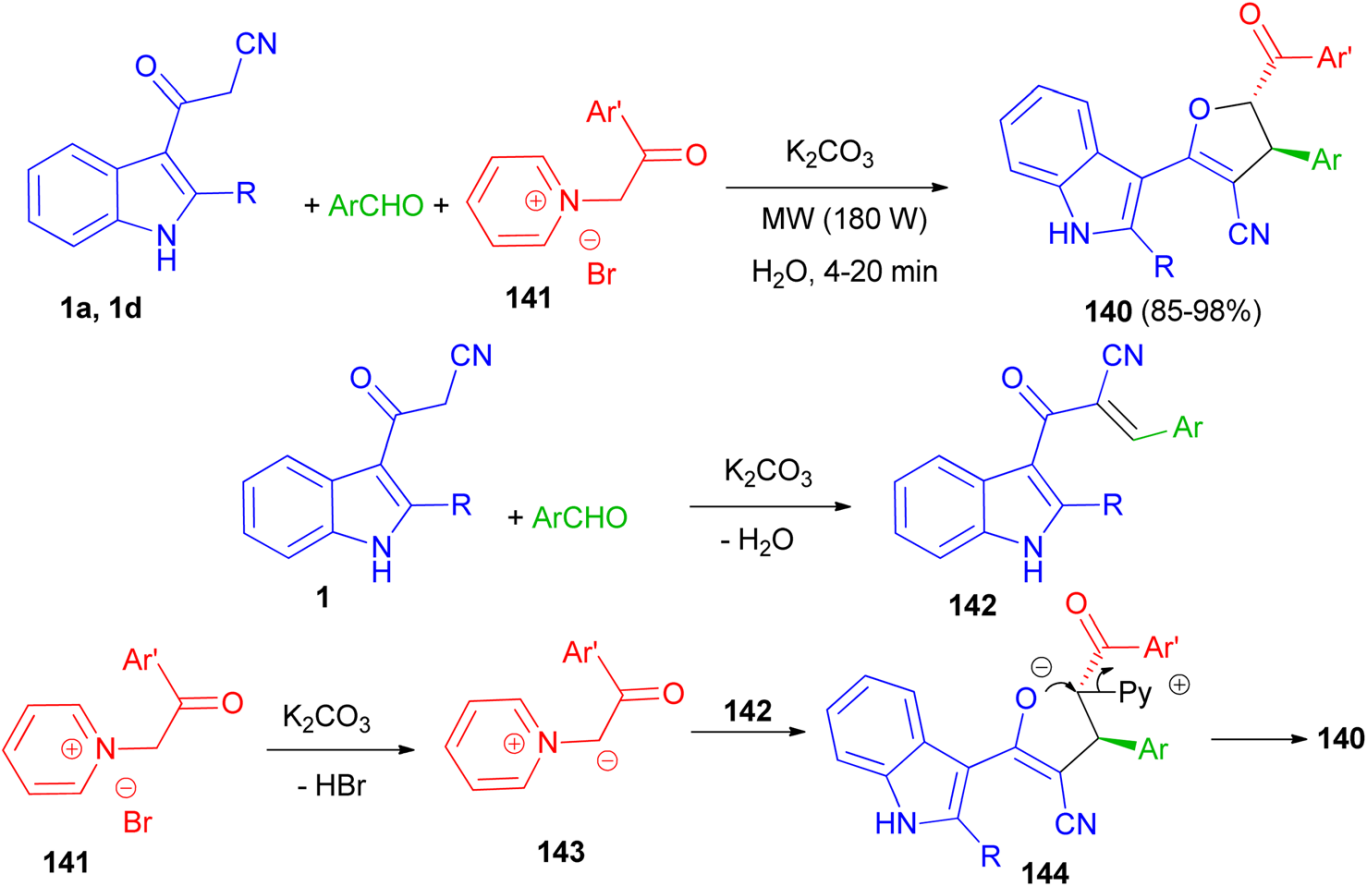
Synthesis of indole-based 4,5-dihydrofurans 140.

Baharfar *et al.* developed an ecofriendly approach for the diastereoselective synthesis of indole-based 4,5-dihydrofurans 140 in 85–98% yields through a three-component reaction of 3-cyanoacetyl indoles 1 with various aldehydes and *N*-phenacylpyridinium bromides 141 in the presence of potassium carbonate as an inexpensive and non-toxic base in water under low power microwave irradiation (180 W) for 4–20 min. The proposed mechanism is outlined in Scheme 52. The Knoevenagel condensation between 1 and aldehyde in the presence of K_2_CO_3_ generates benzylidene 3-cyanoacetyl indole 142. Deprotonation of pyridinium salt 141 by K_2_CO_3_ forms pyridinium ylide 143, which undergoes Michael addition to intermediate 142 to afford dipolar adduct 144. Lastly, the attack of enolate moiety 144 on the electrophilic carbon bearing the leaving pyridyl group gives the final product 140.^86^

**Scheme 53:**
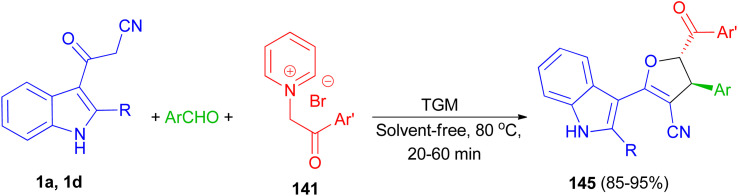
Synthesis of highly functionalized indole based 4,5-dihydrofurans 145.

Next, 1,1,3,3 *N*,*N*,*N*′,*N*′-tetramethylguanidine as catalyst was used for the diastereoselective synthesis of *trans*-indolyldihydrofurans 145 in 85–95% yields by three-component reaction of 3-cyanoacetyl indoles 1 with various aromatic aldehydes and *N*-phenacylpyridinium bromides 141 under solvent-free conditions at 80 °C for 20–60 min. The synthesized compounds exhibited good antioxidant activity, which can be attributed to the acidic hydrogens of their N–H and methine groups. Additionally, presence of either electron-withdrawing or electron-donating substituents on the aromatic ring effectively increased the antioxidant capacity (Scheme 53).^87^

**Scheme 54:**
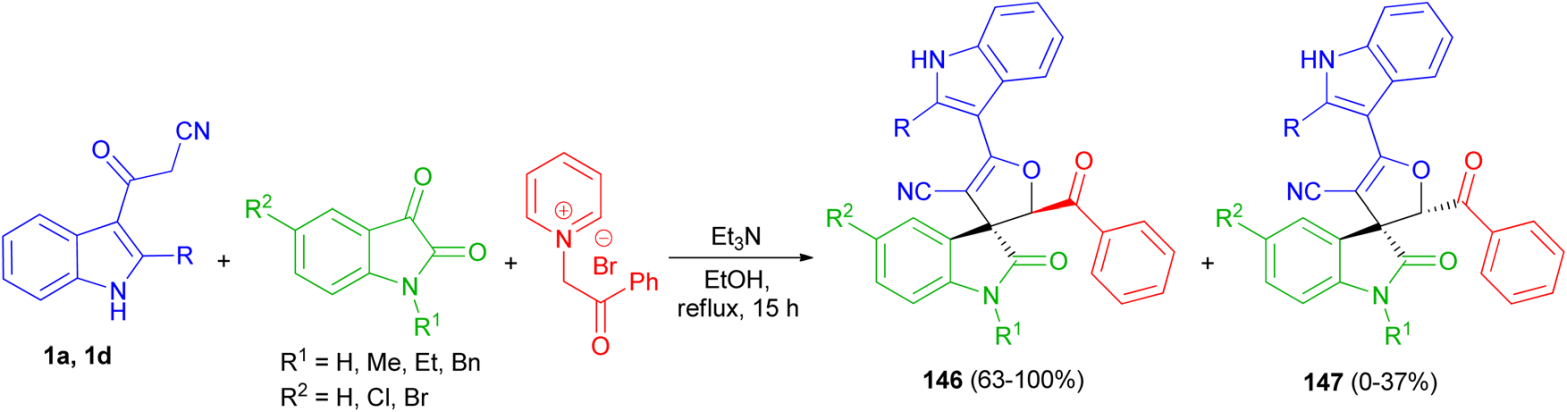
Synthesis of dihydrofuranyl spirooxindoles 146 and 147.

A facile and efficient protocol developed for the diastereoselective synthesis of spirooxindole-dihydrofurans 146 and 147 by the three-component condensation of isatin derivatives, 3-cyanoacetyl indoles 1, and *N*-phenacylpyridinium bromide in the presence of triethylamine base in ethanol under reflux conditions for 15 h. In this transformation, a mixture of diastereomers was detected in most cases (Scheme 54).^88^

### 3.10. Other derivatives

**Scheme 55:**
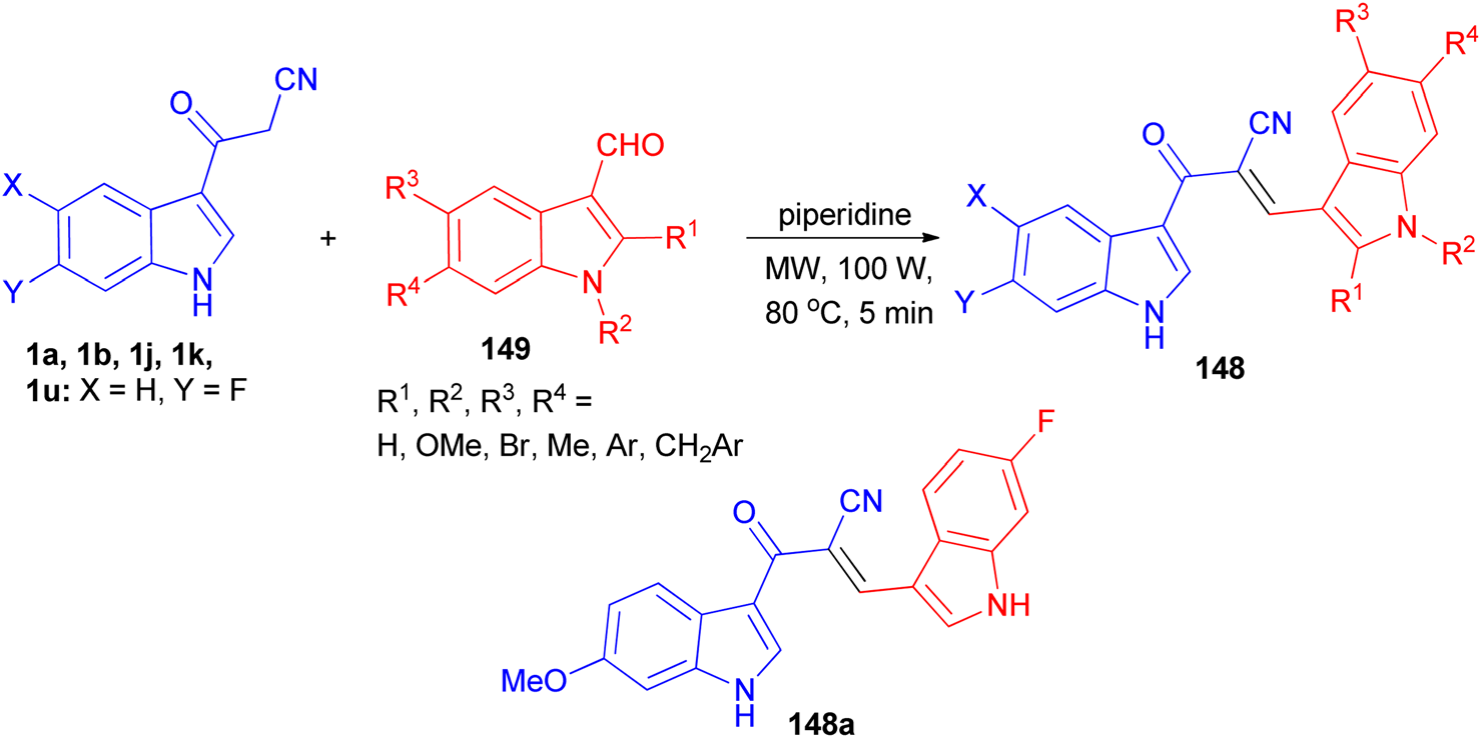
Synthesis of α-cyano bis(indolyl)chalcones 148 as anticancer agents.

Ito *et al.* presented the synthesis of α-cyano bis(indolyl)chalcones 148 in good yields by the reaction of 3-cyanoacetylindole 1 and indole-3-carboxaldehyde 149 in ethylene glycol and piperidine under microwave irradiation with *P* = 100 w/100 psi at 80 °C for 5 min (Scheme 55). Among the synthesized chalcones, compound 148a was found to be the most potent and selective against A549 lung cancer cell line (IC_50_ = 0.8 μM). In a preliminary mechanism of action studies some α-cyano bis(indolyl)chalcones were found to enhance tubulin polymerization suggesting these compounds could act as microtubule stabilizing agents.^89^

**Scheme 56:**
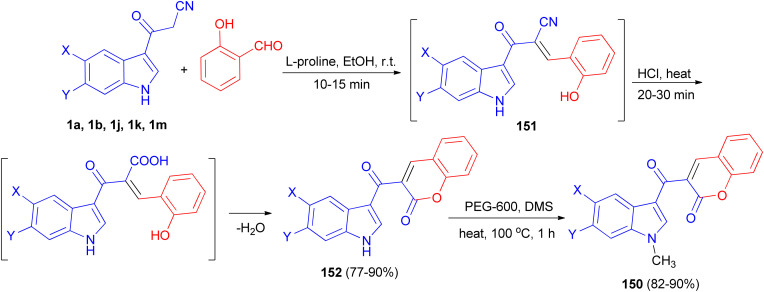
Synthesis of 3-acetylcoumarinoindoles 150.

A tandem synthesis of 3-acetylcoumarinoindoles 150 in the presence of catalytic amount of l-proline in ethanol medium is reported. l-Proline has been utilized as an efficient and eco-friendly catalyst for the Knoevenagel condensation of 3-cyanoacetylindoles 1 with 2-hydroxybenzaldehyde to afford the corresponding substituted 3-(1*H*-indol-3-yl)2-(2-hydroxybenzylidene)-3-oxopropanenitriles 151, which without isolation were treated with hot conc. HCl to afford 3-acetylcoumarinoindoles 152 in 77–90% yields. Subsequently, these were reacted with dimethyl sulfate in the presence of PEG-600 as an efficient and green solvent at 100 °C for 1 h to afford the corresponding *N*-methyl-3-acetylcoumarinoindoles 150 in 82–90% yields (Scheme 56).^90^

**Scheme 57:**
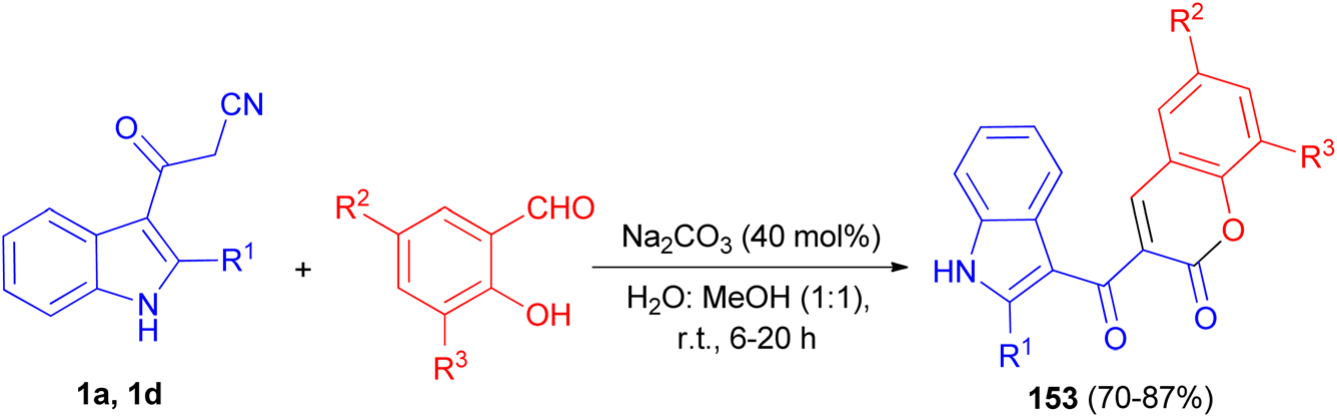
Preparation of 3-(1*H*-indole-3-carbonyl)-2*H*-chromen-2-one derivatives 153.

A simple and convenient method described for the one-pot synthesis of 3-(1*H*-indole-3-carbonyl)-2*H*-chromen-2-one derivatives 153 in 70–87% yields from the reaction of 3-cyanoacetyl indoles (1) and salicylaldehyde derivatives in the presence of Na_2_CO_3_ in water: methanol (1 : 1) at room temperature for 6–20 h (Scheme 57). The synthesized compounds exhibited good radical scavenging ability against DPPH free radical, antibacterial activity against Gram-positive bacteria (MRSA), *Bacillus* sp. and Gram negative bacterial strains (*Escherichia coli*, *Klebsiella pneumoniae*) and antifungal activity against *Candida albicans*.^91^

**Scheme 58:**
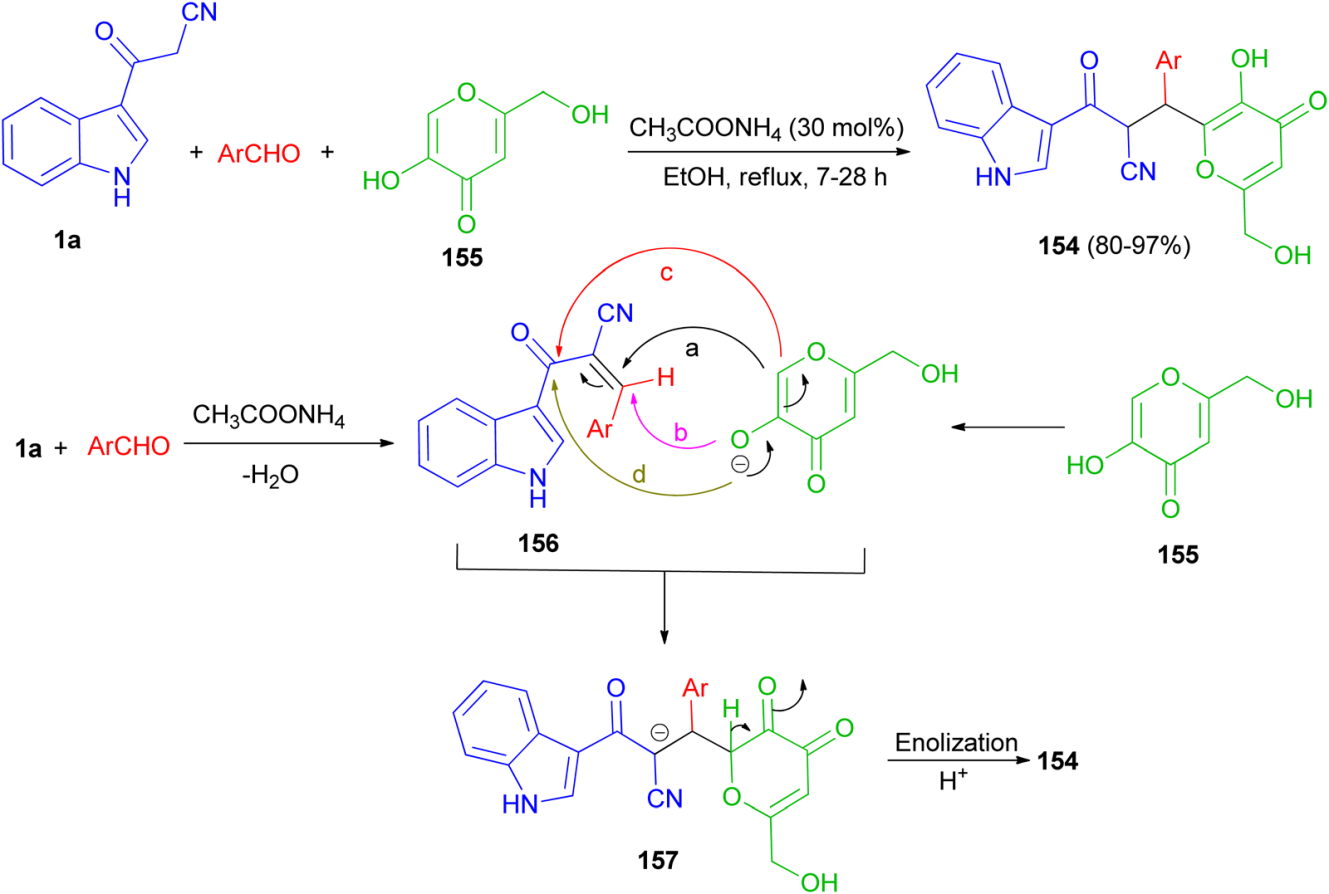
Regioselective synthesis of 3-(cyanoacetyl)indole derivatives 154.

A protocol for the regioselective synthesis of diastereomeric 3-substituted indole derivatives 154 in 80–97% yields was described by the three-component condensation of 3-(cyanoacetyl)indole (1a), aromatic aldehydes, and kojic acid (155) in the presence of ammonium acetate as catalyst in refluxing EtOH for 7–28 h. Some of the synthesized compounds demonstrated excellent activity against *S. aureus*, *B. subtilis*, antibacterial activities against *P. aeruginosa* and good activity against *E. coli*. A plausible mechanism is given in Scheme 58. The formation of these products can be rationalized by initial formation of intermediate 156 by Knoevenagel condensation of the aldehyde and 1a. The anion of kojic acid can attack to intermediate 156 in four routes: Michael-type addition (route a), direct addition (route b), *C*-alkylation (route c), or *O*-alkylation (route d). Among these four routes, the reaction proceeded regioselectively *via* route a, and led to intermediate 157. Then, enolization and protonation of 157 in the reaction conditions result in final product 154.^92^

**Scheme 59:**
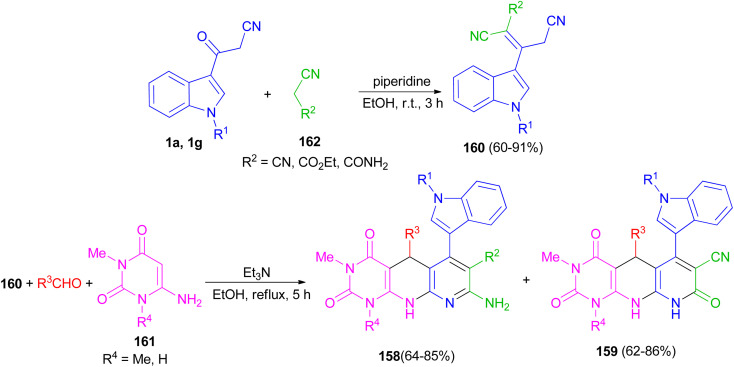
Synthesis of hexahydropyrimido[4,5-*b*]-1,8-naphthyridines 158 and 159.

Naidu and co-workers provided a procedure for the synthesis of hexahydropyrimido[4,5-*b*]-1,8-naphthyridine derivatives 158 and 159 in 62–86% yields by a one-pot three-component reaction of a 2-cyano-3-(1*H*-indol-3-yl)-pent-2-enedinitrile or ethyl-2,4-dicyano-3-(1*H*-indol-3-yl)but-2-enoate derivative 160 with an aryl aldehyde and a 6-aminouracil derivative 161 in the presence of Et_3_N in refluxing EtOH for 5 h. Indoles 160 were synthesized by treating the corresponding 1 with the appropriate acetonitrile derivative 162 in the presence of piperidine as a catalyst in EtOH at room temperature for 3 h (Scheme 59).^93^

**Scheme 60:**
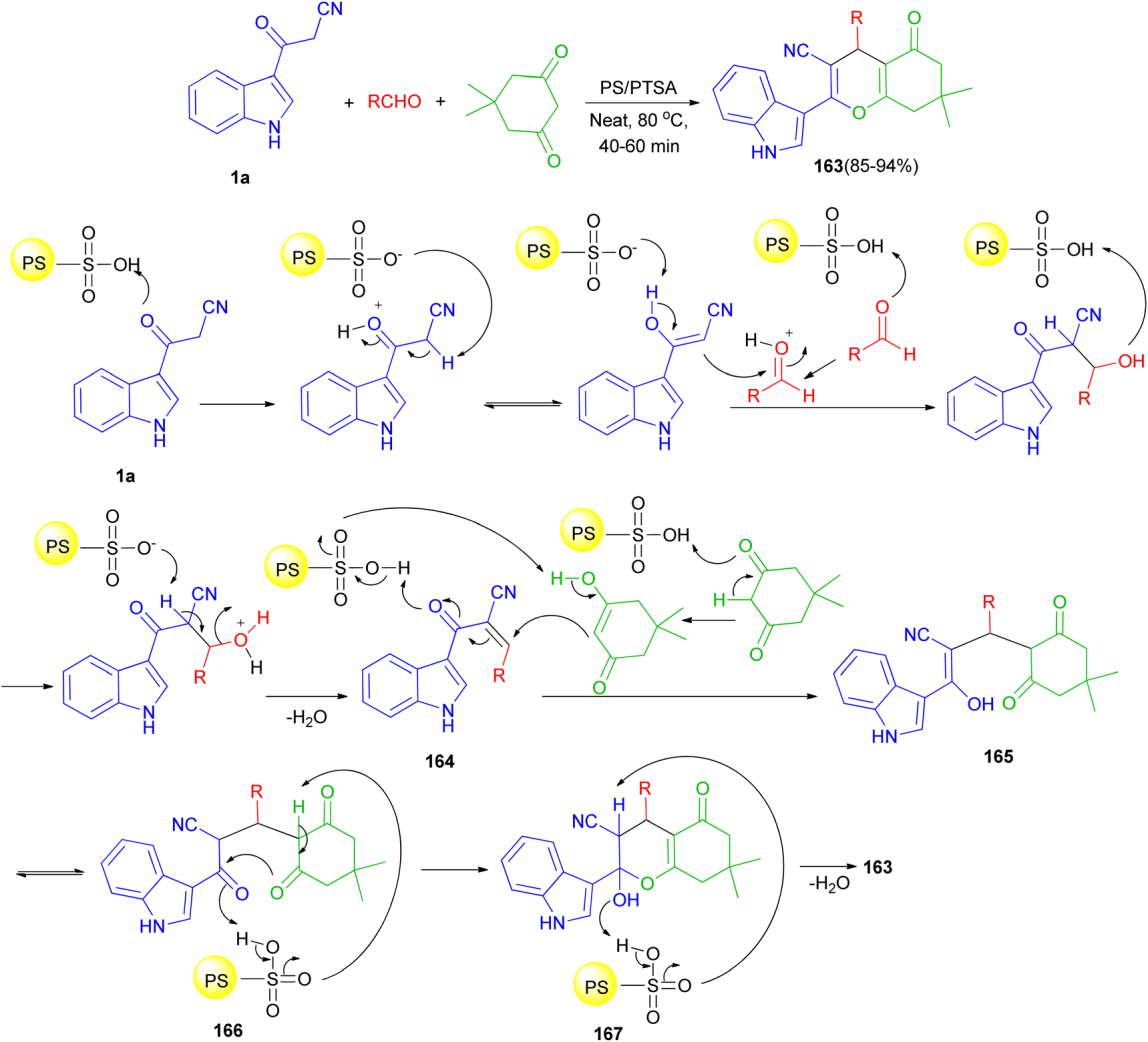
Schematic illustration of reaction mechanism for the product 163.

In 2016, Reddy *et al.* described an efficient and environmentally benign protocol for the synthesis of diversely functionalized 1*H*-indol-3-yl-4*H*-chromene-3-carbonitriles 163 in 85–94% yields through one-pot three-component condensation reaction of 1a, 5,5-dimethylcyclohexane-1,3-dione and various aromatic, aliphatic and heterocyclic aldehydes in the presence of polystyrene-supported *p*-toluenesulfonic acid (PS/*P*TSA) under solvent-free conditions at 80 °C for 40–60 min. A plausible mechanism is shown in Scheme 60. Initially in the presence of PS/*P*TSA catalyst 1a is reacts with aldehydes to give an α,β-unsaturated ketone 164. On subsequent Michael addition of enolic form of 5,5-dimethylcyclohexane-1,3-dione to this conjugated intermediate, 164, forms enolic intermediate 165 which on keto–enol tautomerisation (K.E.T.) gives triketone, 166, intermediate. In the presence of catalyst, this intermediate undergoes intramolecular cyclisation and gives unstable, 167, which on dehydration affords the desired product 163.^94^

**Scheme 61:**
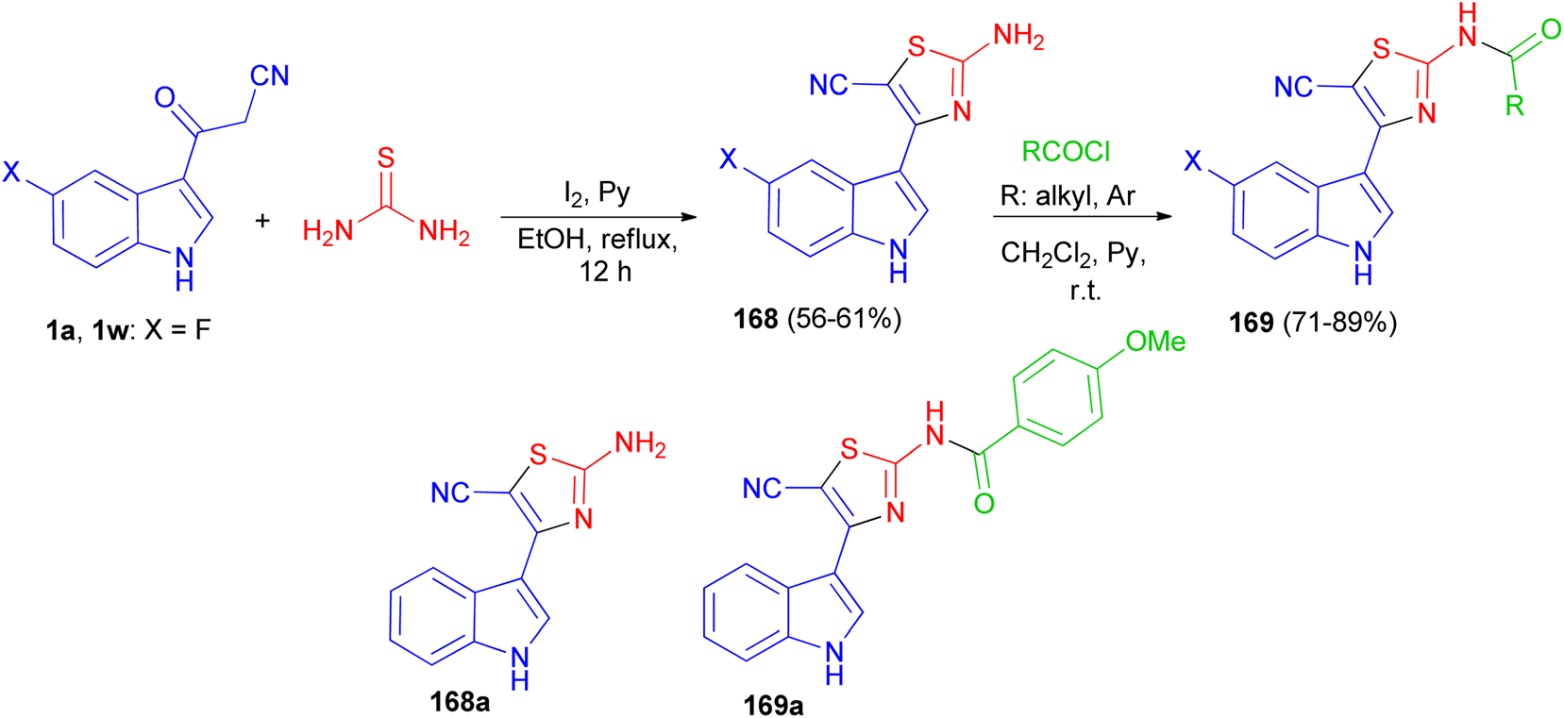
Synthesis of thiazole derivatives containing indole moiety 168 and 169.

After that, Gururaja and co-workers reported synthesis of 2-amino-4-(1*H*-indole-3-yl) thiazole-5-carbonitrile derivatives 168 in 56–61% yields by the reaction of 3-(1*H*-indole-3-yl)-3-oxopropanenitrile derivatives 1 and thiourea in the presence of pyridine and iodine in refluxing EtOH for 12 h. Then, the reaction of 168 with carboxylic acid chloride in CH_2_Cl_2_ in the presence of pyridine at room temperature afforded *N*-(5-cyano-4-(1*H*-indole-3-yl)thiazol-2-yl) substituted amides 169 in 71–89% yields. The synthesized indole-thiazole derivatives were evaluated for cytotoxicity effect on breast cancer cells and found that, compounds 168a and 169a are moderately toxic and the others are less toxic to the breast cancer cells (Scheme 61).^95^

**Scheme 62:**
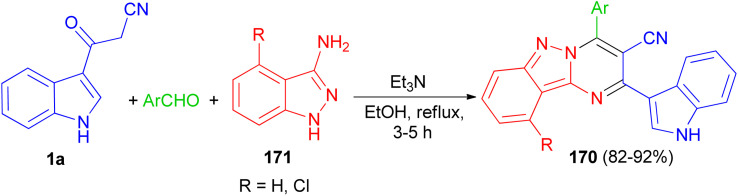
Synthesis of 4-arylpyrimido[1,2-*b*]indazole-3-carbonitrile derivatives 170.

In 2017, Rong *et al.* utilized an efficient metal-free cascade reaction to synthesize pyrimido[1,2-*b*]indazole-3-carbonitrile derivatives 170. The reaction of aromatic aldehydes, 1*H*-indazol-3-amine (4-chloro-1*H*-indazol-3-amine) 171 and 1a was carried out in refluxing EtOH in the presence of triethylamine to afford 170 in 82–92% yields after 3–5 h (Scheme 62).^96^

**Scheme 63:**
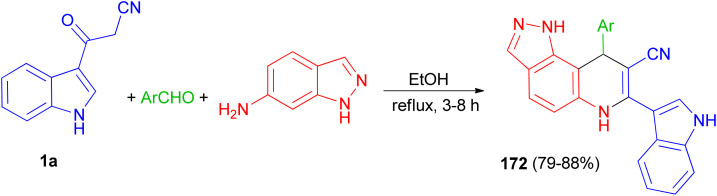
Synthesis of pyrazolo[3,4-*f*]quinoline-8-carbonitriles 172.

Liu and co-workers presented the synthesis of pyrazolo[3,4-*f*]quinoline-8-carbonitriles 172 in 79–88% yields by the reaction of an aromatic aldehyde, 1*H*-indazol-6-amine and 1a in ethanol under reflux conditions for 3–8 h (Scheme 63).^97^

**Scheme 64:**
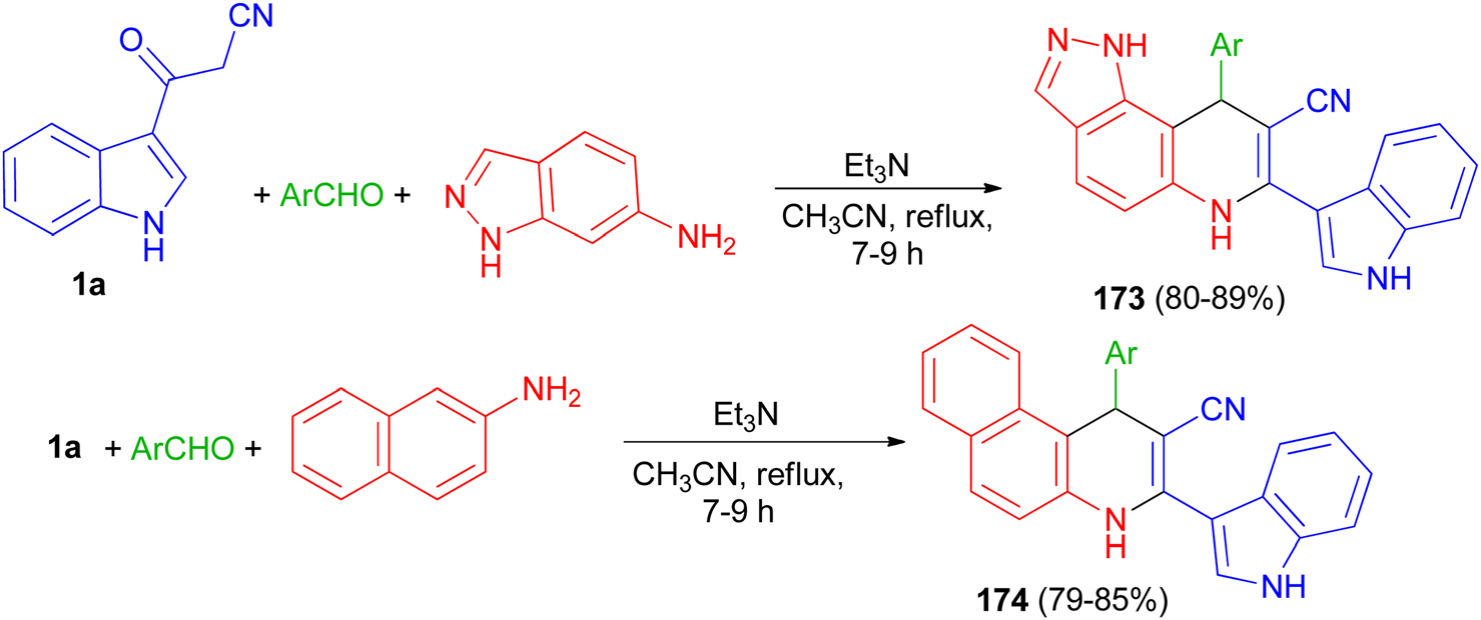
Synthesis of 9-aryl-6,9-dihydro-1*H*-pyrazolo[3,4-f]quinoline-8-carbonitriles 173 and 1-aryl-1,4-dihydrobenzo[f]quinoline-2-carbonitriles 174.

Rong *et al.* reported synthesis of 9-aryl-6,9-dihydro-1*H*-pyrazolo[3,4-f]quinoline-8-carbonitrile derivatives 173 in 80–89% yields by the one-pot three-component reaction of aromatic aldehydes, 1*H*-indazol-6-amine and 3-cyanoacetyl indole (1a) and 1-aryl-1,4-dihydrobenzo[*f*]quinoline-2-carbonitrile derivatives 174 in 79–85% yields *via* the reaction of various aromatic aldehydes, 2-naphthylamine and 1a using Et_3_N in CH_3_CN at 80 °C for 7–9 h (Scheme 64).^98^

**Scheme 65:**
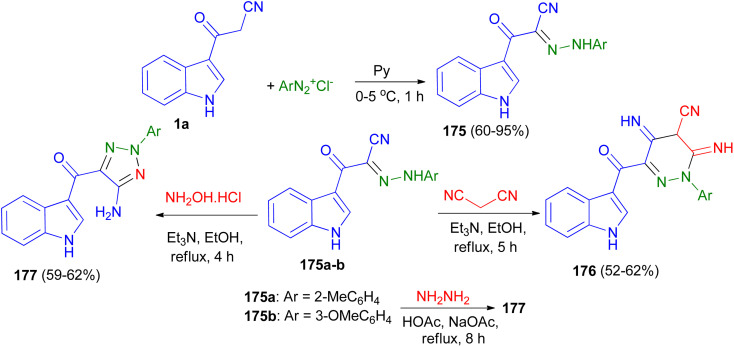
Synthesis of hydrazo, dihydropyridazinyl and triazolyl derivatives containing indole nucleus 175–177.

Fadda *et al.* designed the synthesis of *N*-aryl-2-(1*H*-indol-3-yl)-2-oxoaceto hydrazonoyl cyanide derivatives 175 in 60–95% yields utilizing the reaction of 1a and aryl diazonium salts in pyridine at 0–5 °C for 1 h. Also, synthesis of 2-aryl-3,5-diimino-6-(1*H*-indole-3-carbonyl)-2,3,4,5-tetrahydropyridazine-4-carbonitrile derivatives 176 in 52–62% yields accomplished by the reaction of 175a–b with malonitrile in ethanol in the presence of triethylamine as catalyst under reflux conditions for 5 h. The same research group also reported synthesis of (5-amino-2-aryl-2*H*-1,2,3-triazol-4-yl)(1*H*-indol-3-yl)methanone derivatives 177 in 59–62% yields *via* the reaction of 175a–b with hydroxylamine hydrochloride in refluxing ethanol in the presence of catalytic amount of triethylamine for 4 h or with hydrazine hydrate in acetic acid in the presence of sodium acetate under reflux conditions for 8 h (Scheme 65). The obtained results clearly revealed that arylazo derivatives with electron withdrawing group exhibited better antimicrobial activity than compounds not containing this groups.^99^

**Scheme 66:**
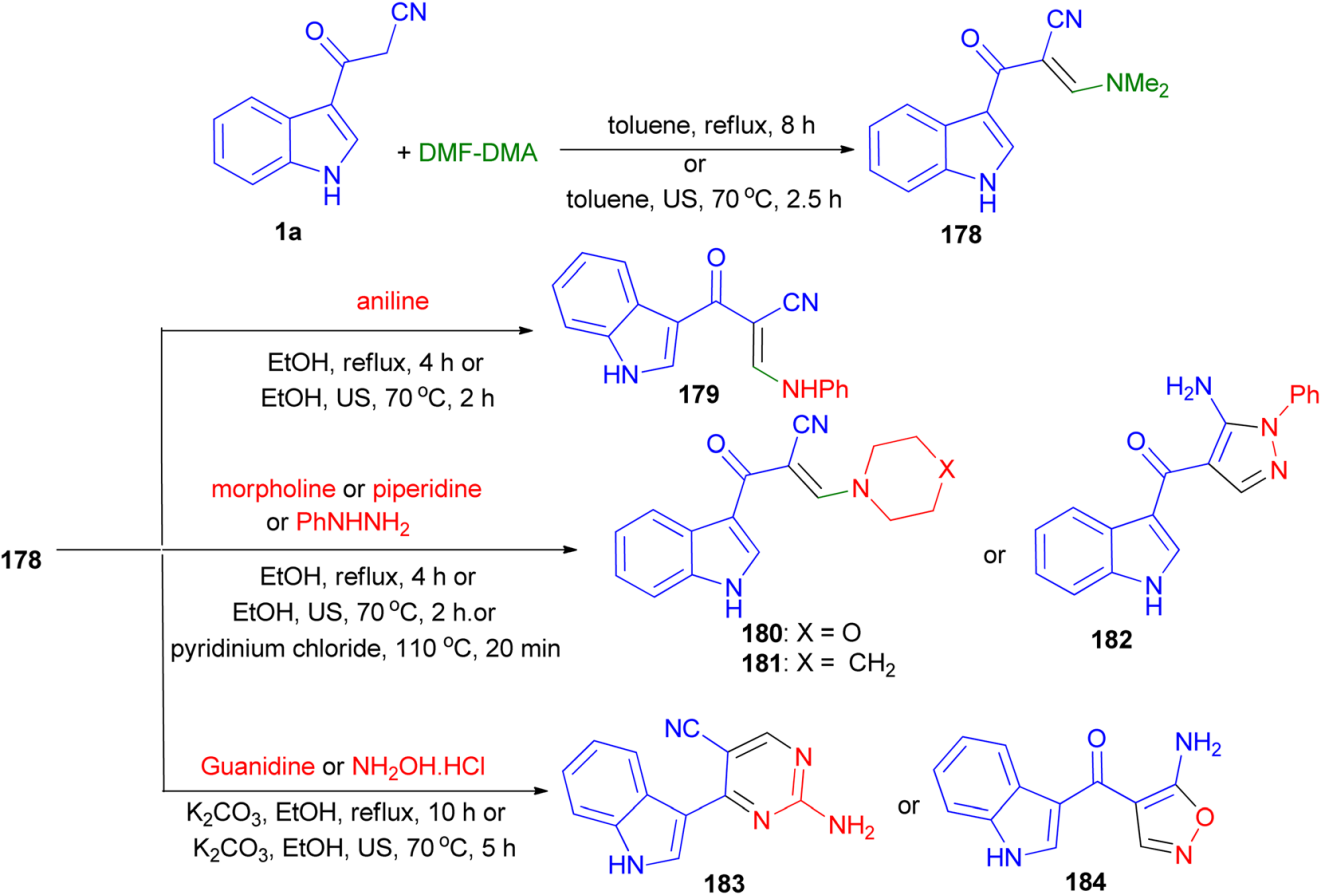
Synthesis of enaminones 178–181 and heterocyclic compounds 182–184.

The condensation reaction of 1a with dimethylformamide-dimethylacetal (DMF-DMA) gave the corresponding enaminone 178. Nucleophilic substitution of 178 with different amines (aniline, piperidine and morpholine) resulted enaminones 179–181. Treatment of compounds 178 with phenylhydrazine afforded the pyrazole derivatives 182. On the other hand, reacting 178 with guanidine gave the pyrimidine 183. Treatment of compound 178 with hydroxylamine hydrochloride afforded the aminoisoxazoles 184. The foregoing reactions were carried out with conventional heating and under green conditions [ultrasound (US) irradiations or ionic liquids (ILs)] (Scheme 66).^100^

**Scheme 67:**
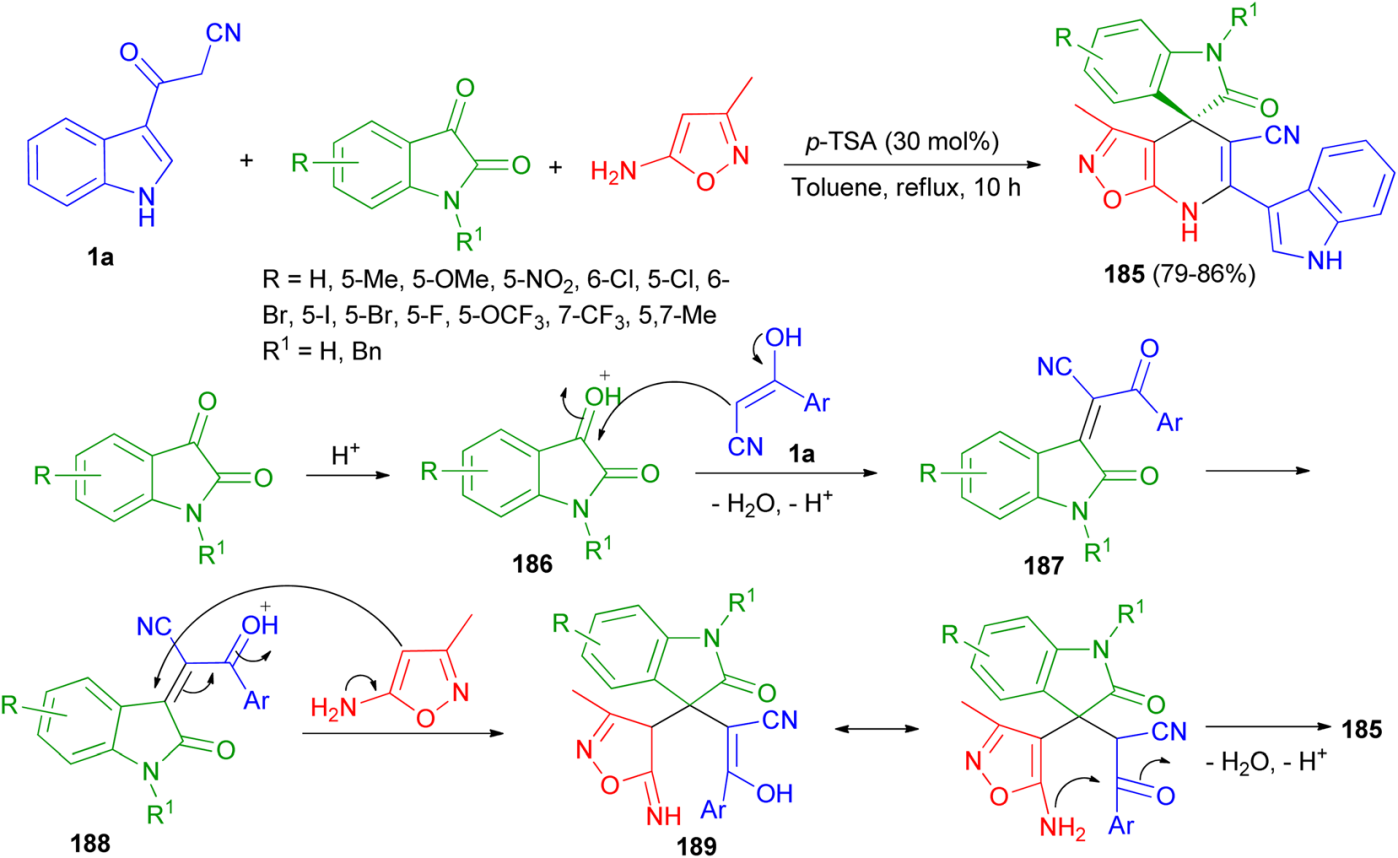
Chemo-selective synthesis of indoline-3,4′-isoxazolo[5,4-*b*]pyridine fused spirooxindole derivatives 185.

A highly convergent and efficient protocol reported for the facile chemoselective synthesis of a library of indoline-3,4′-isoxazolo[5,4-*b*]pyridine fused spirooxindole derivatives 185 in 79–86% yields by three-component reaction of 1a, isatin derivatives and 3-methylisoxazol-5-amine using *p*-TSA (30 mol%) in refluxing toluene for 10 h. A plausible mechanism is outlined in Scheme 67. First, the nucleophilic addition of aryl acetonitrile into protonated isatin 186 followed by elimination of water gives intermediate 187, which upon further protonation at ketone to yield intermediate 188. The intermediate 188 on Michael addition with isooxazole-5-amine to generate intermediate 189, which undergoes intra-molecular cyclisation and dehydration to afford desired product 185.^101^

**Scheme 68:**
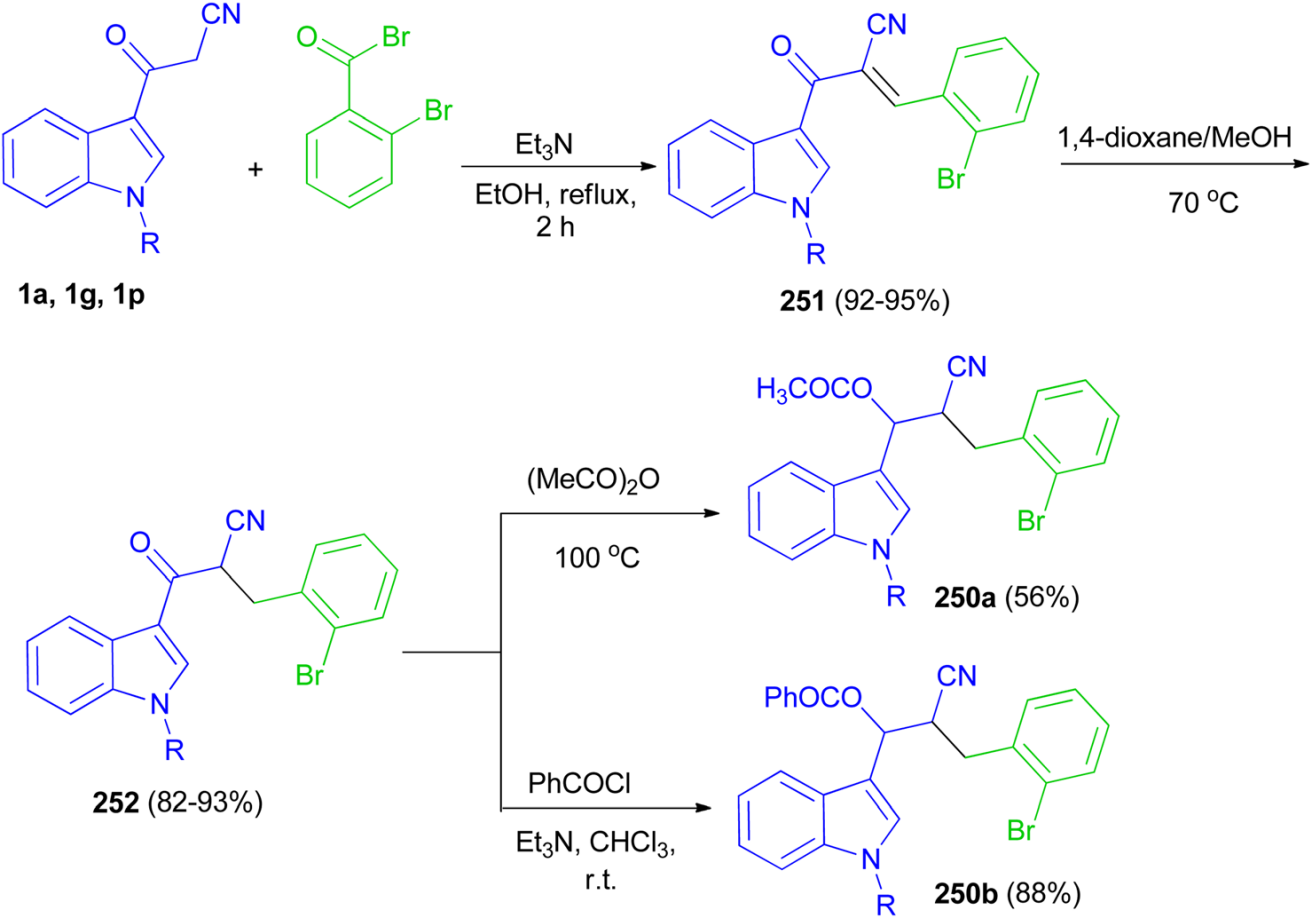
Synthesis of indole-based cyano derivatives 250–252.

Glidewell and co-workers developed three-step sequence for the preparation of the esters 190 starting by the reaction of 3-(indol-3-yl)-3-oxopropanenitriles 1 with 2-bromobenzaldehyde in the presence of Et_3_N in refluxing EtOH for 2 h to form the corresponding chalcones 191 in 92–95% yields; these are readily reduced to dihydrochalcones 192 in 82–93% yields in the presence of NaBH_4_ in 1,4-dioxane and methanol at 70 °C, which are in turn acylated to form the enolate esters 190 in 56–88% yields (Scheme 68).^102^

**Scheme 69:**
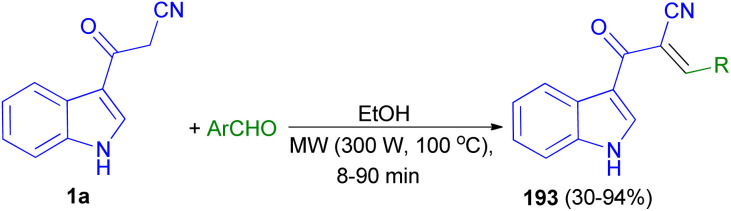
Synthesis of (*E*)-2-(1*H*-indole-3-ylcarbonyl)-3-heteroaryl-acrylonitriles 193.

Stereoselective synthesis of (*E*)-2-(1*H*-indole-3-ylcarbonyl)-3-heteroaryl-acrylonitriles 193 obtained in 30–94% yields from the reaction of 1a with heteroaryl-aldehydes in EtOH under microwave assisted Knoevenagel reaction at 300 W of potency and 100 °C for 8–90 min (Scheme 69).^103^

**Scheme 70:**
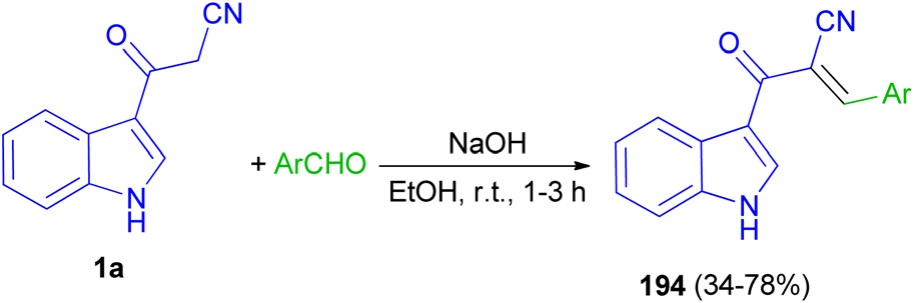
Synthesis of indole acrylonitriles 194.

Mohammed Khan and co-workers reported synthesis of indole acrylonitriles 194 in 34–78% yields by the reaction of 1a and aryl aldehydes in the presence of NaOH in absolute ethanol at room temperature for 1–3 h (Scheme 70). The synthetic molecules have shown promising α-glucosidase enzyme inhibitory activity in the range of (IC_50_ = 0.53 ± 0.01–1.36 ± 0.04 μM) as compared to the standard acarbose (IC_50_ = 2.91 ± 0.02 μM).^104^

**Scheme 71:**
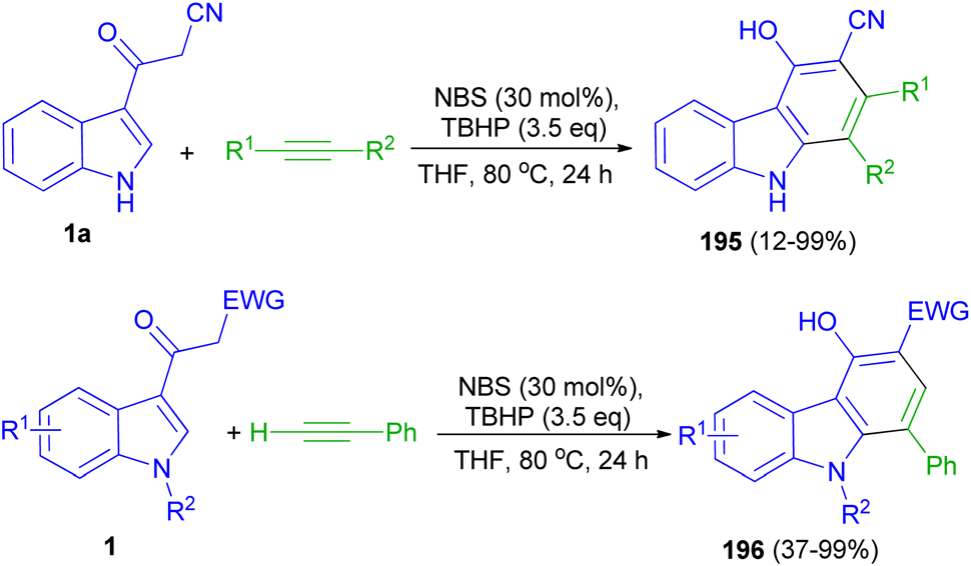
Synthesis of carbazoles 195 and 196.

Wang and co-workers applied *N*-bromosuccinimide for the intermolecular annulation of acetyl indoles 1 with alkynes in the presence of TBHP in THF at 80 °C for 24 h, allowing for regioselective formation of valuable carbazoles 195–196 (up to 99% yield) through direct C–H bond functionalization (Scheme 71). Mechanistic investigations indicate that the bromination of acetyl indole takes place to generate a bromide intermediate, followed by coupling with an alkyne and intramolecular cycloaromatization to furnish carbazole products.^105^

**Scheme 72:**
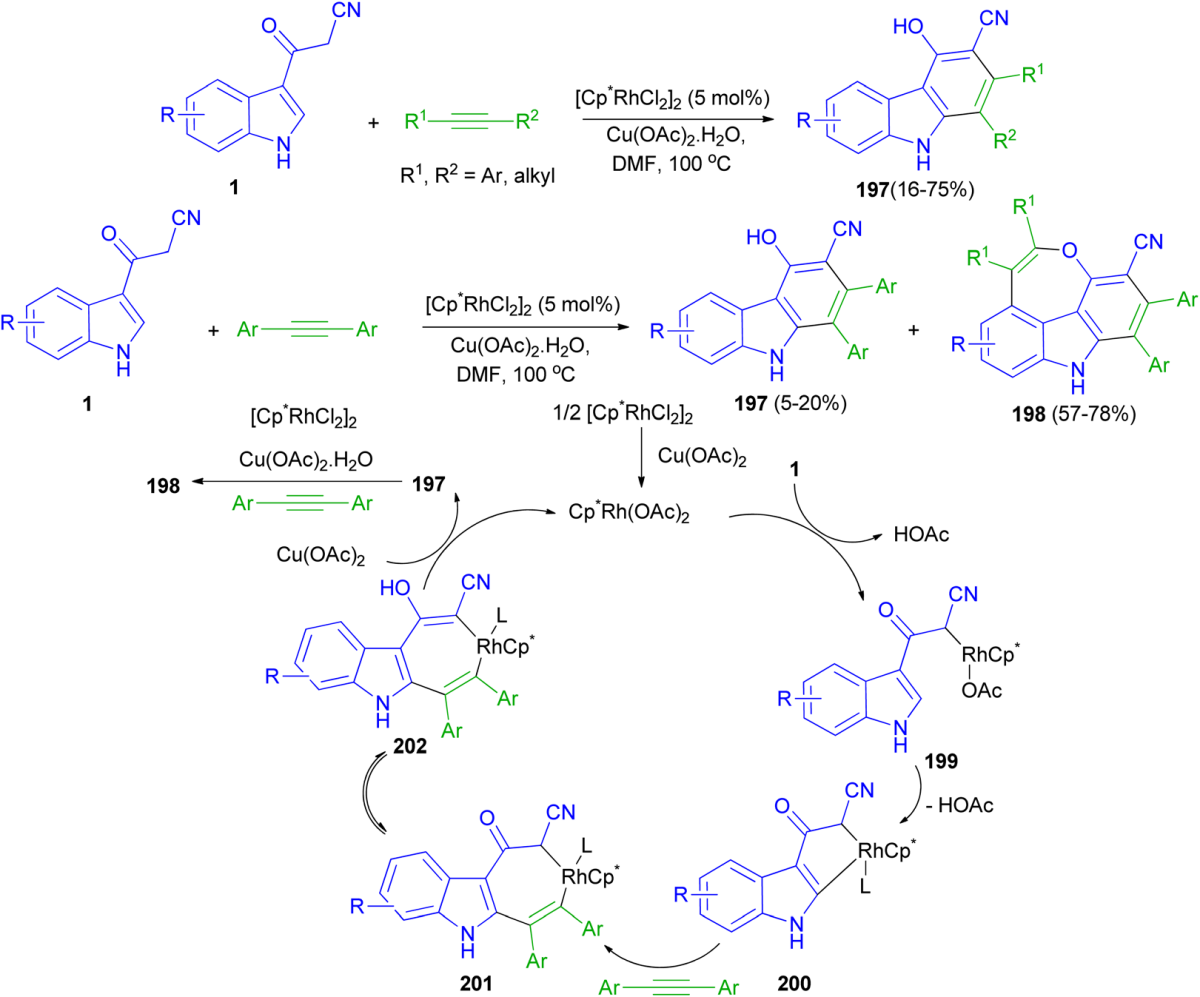
Rh(iii)-catalyzed synthesis of carbazoles 197 and 4*H*-oxepino[2,3,4,5-def]carbazoles 198.

Rh(iii)-catalyzed [4 + 2] cycloaddition reactions of 3-(1*H*-indol-3-yl)-3-oxopropanenitriles 1 with alkyne in the presence of Rh(iii) as catalyst in DMF at 100 °C afforded substituted carbazoles 197 in 16–75% yields. Also, the reaction of 1 with two molecules of alkynes resulted 4*H*-oxepino[2,3,4,5-def]carbazoles 198 (57–78% yields) and 197 (5–20 mol% yields) *via* tandem [4 + 2] and [5 + 2] cycloaddition under the same reaction conditions. A possible mechanism is proposed as shown in Scheme 72. The first step is likely to be the acidic C(sp^3^)–H bond activation process affording intermediate 199, then C(sp^2^)–H bond activation through the CMD mechanism gives a five membered rhodacycle 200. The coordination and insertion of an alkyne into 200 leads to the seven-membered rhodacycle intermediate 201. After ketone enolization intermediate 202 is formed and undergoes reductive elimination to afford product 197 and Cp*Rh(i). Cp*Rh(i) is oxidized by Cu(OAc)_2_·H_2_O to Cp*Rh(OAc)_2_ for the next catalytic cycle. The mechanism for the second-step annulation of 197 with an alkyne to form 198 is similar to the [5 + 2] cycloaddition reaction of *o*-vinylphenol with an alkyne.^106^

**Scheme 73:**
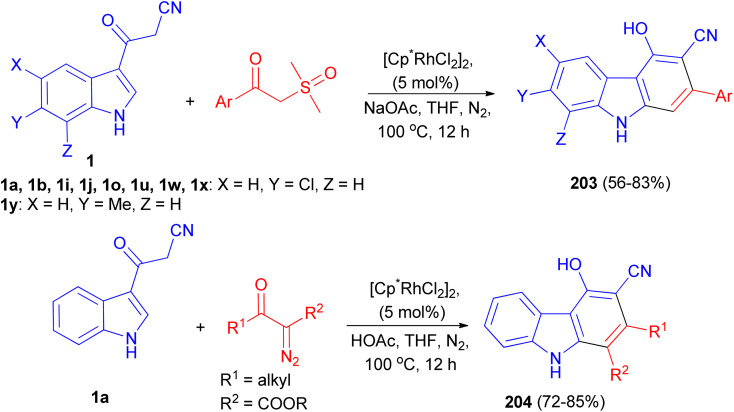
Synthesis of polysubstituted carbazoles 203 and 204.

A rhodium-catalyzed annulation of 3-(1*H*-indol-3-yl)-3-oxopropanenitriles with sulfoxonium ylides or diazo compounds has been developed, leading to a series of polysubstituted carbazoles 203 and 204 in moderate to good yields. This procedure proceeded with formal Rh(iii)-catalyzed [4 + 2] cycloaddition, with the functionalization of 2-C–H bonds of indole in a step-economical procedure. Additionally, this reaction could also be conducted under acidic conditions in THF at 100 °C for 12 h when diazo compounds were employed as the reaction partners, which was a complement to the annulation of sulfoxonium ylides under weak basic conditions in the presence of NaOAc in THF at 100 °C for 12 h (Scheme 73).^107^

**Scheme 74:**
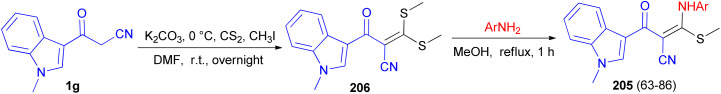
Synthesis of *N*-methyl indole derivatives 205.

Patel *et al.* described synthesis of 3-((substitutedphenyl)amino)-2-(1-methyl-1*H*-indole-3-carbonyl)-3-(methylthio)acrylonitrile derivatives 205 in 63–86% yields. At first, the reaction of 3-cyanoacetyl *N*-methyl indole 1g with carbon disulphide and methyl iodide in basic condition affords 2-(1-methyl-1*H*-indole-3-carbonyl)-3,3-bis(methylthio)acrylonitrile 206 as a scaffold. Subsequent, the scaffold when reacts with substituted various amine derivatives *via* desulfitative displacement forms the desired product 205 (Scheme 74). The antibacterial activity of all compounds showed promising activity in comparison to standard drug streptomycin and ciprofloxacin, while the antifungal activity of all compounds showed higher to moderate activity against standard drug nystatin.^108^

**Scheme 75:**
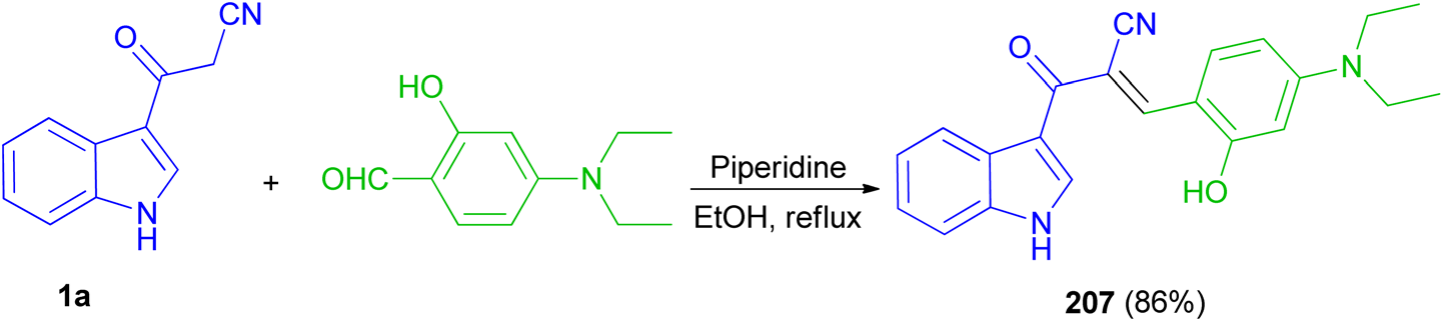
Synthesis of fluorescent probe based on cyanoacetyl indole derivative 207.

Recently, a fluorescent probe based on cyanoacetyl indole derivative 207 was designed and synthesized by the reaction of 3-cyanoacetylindole (1a) with 4-diethylaminosalicylic aldehyde using anhydrous ethanol as solvent and piperidine as a catalyst under reflux temperature. Probe displayed good stability, highly selectivity and sensitivity for detection of HPO_4_^−2^ through fluorescence quenching, and other thirteen anions including H_2_PO_4_^−^, NO_3_^−^, NO_2_^−^, HSO_3_^−^, SO_3_^−2^, SO_4_^−2^, S_2_O_3_^−2^, AcO^−^, F^−^, Cl^−^, Br^−^, I^−^, SCN^−^ was basically undisturbed and only occurred weak fluorescence changes. The protonation of nitrile may afford nitrilium ion (Scheme 75).^109^

## 4. Conclusion

This review represents a comprehensive documentation to the recent development for the synthesis of 3-cyanoacetyl indoles (CAIs) and applications for the construction of variety of novel molecules containing indole moieties such as pyranes, pyridines, dihydropyridines, pyrimidines, tetrahydropyrimidines, pyrazoles, pyrazolopyridines, pyrazolopyrimidines, pyridopyrimidines, tetrazolopyrimidines, triazolopyrimidines, furans, dihydrofurans, coumarins, pyrimidonaphthyridines, chromenes, thiazoles, pyrimidoindazoles, pyrazoloquinolines, isoxazolopyridines and carbazoles *via* the different classical methods with green approach, homogeneous, heterogeneous and metal-catalyzed reactions, ultrasound-mediated and microwave irradiation reactions during the period of 2013–2022. A large portion of these molecules exhibits promising biological and pharmaceutical activities such as antioxidant, anticancer, antiproliferative, antimicrobial, α-glucosidase enzyme inhibitor, anti-inflammatory, antibacterial activity against both Gram-negative and Gram-positive bacteria, anti-biofilm, cytotoxic potential against breast carcinoma (MCF-7) and as potential inhibitors of Nicotinamide phosphoribosyltransferase (NAMPT). It is hoped that more interesting results in this review will help not only to the synthetic chemists but also to the medicinal and pharmaceutical chemists to update information on recent developments in this field as well as to study the potential biological activities of such compounds reported in this review.

## Conflicts of interest

There are no conflicts to declare.

## Supplementary Material

## References

[cit1] Samala S., Arigela R. K., Kant R., Kundu B. (2014). J. Org. Chem..

[cit2] Sharma V., Pradeep K., Devender P. (2010). J. Heterocycl. Chem..

[cit3] Kaushik N. K., Kaushik N., Attri P., Kumar N., Kim C. H., Verma A. K., Choi E. H. (2013). Molecules.

[cit4] Wang S.-Y., Shi X.-C., Laborda P. (2020). Eur. J. Med. Chem..

[cit5] Xie W., Pakdel E., Liang Y., Kim Y. J., Liu D., Sun L., Wang X. (2019). Biomacromolecules.

[cit6] Lee H. M., Chiu P. L., Hu C.-H., Lai C.-L., Chou Y.-C. (2005). J. Organomet. Chem..

[cit7] Fadda A. A., El-Mekabaty A., Mousa I. A., Elattar K. M. (2014). Synth. Commun..

[cit8] Lakhdar S., Westermaier M., Terrier F., Goumont R., Boubaker T., Ofial A. R., Mayr H. (2006). J. Org. Chem..

[cit9] Wan X., Sun M., Wang J.-Y., Yu L., Wu Q., Zhang Y.-C., Shi F. (2021). Org. Chem. Front..

[cit10] Wang W., Han H., Wang L., Wang Q., Bu Z. (2021). Chin. J. Org. Chem..

[cit11] Zhang Z., Liu X., Zong X., Yuan Y., Liu S., Zhang T., Wu Z., Yang J., Jia Z. (2021). Chin. J. Org. Chem..

[cit12] Zhang J., Wu H., Zhang W., Wang L., Jin Y. (2021). Chin. J. Org. Chem..

[cit13] Guo J.-M., Wang W.-B., Guo J., Zhu Y.-S., Bai X.-G., Jin S.-J., Wang Q.-L., Bu Z.-W. (2018). RSC Adv..

[cit14] Wang W., Bai X., Jin S., Guo J., Zhao Y., Miao H., Zhu Y., Wang Q., Bu Z. (2018). Org. Lett..

[cit15] Wang W., Zhang M., Yang W., Yang X. (2022). Chin. J. Org. Chem..

[cit16] Du Y., Xiao Y., Tian F., Han L., Gu Y., Zhu N. (2021). Chin. J. Org. Chem..

[cit17] Huang Y., Li J., Li S., Ma J. (2021). Chin. J. Org. Chem..

[cit18] Wang H.-Q., Wu S.-F., Yang J.-R., Zhang Y.-C., Shi F. (2023). J. Org. Chem..

[cit19] Zhu Y.-S., Yuan B.-B., Guo J.-M., Jin S.-J., Dong H.-H., Wang Q.-L., Bu Z.-W. (2017). J. Org. Chem..

[cit20] Shiri M., Zolfigol M. A., Gerhardus Kruger H., Tanbakouchian Z. (2010). Chem. Rev..

[cit21] Naim M. J., Alam O., Jahangir Alam Md., Bano F., Alam P., Shrivastava N. (2016). Int. J. Pharm. Sci. Res..

[cit22] Mohammadi Ziarani G., Moradi R., Ahmadi T., Lashgari N. (2018). RSC Adv..

[cit23] Thanikachalam P. V., Maurya R. K., Garg V., Monga V. (2019). Eur. J. Med. Chem..

[cit24] Kumar P., Nagtilak P. J., Kapur M. (2021). New J. Chem..

[cit25] Ghiron C. , Nencini A., Micco I., Zanaletti R., Maccari L., Bothmann H., Haydar S., Varrone M., Pratelli C. and Harrison B., WO2008/87529, 2008

[cit26] Behbehani H., Ibrahim H. M., Makhseed S., Mahmoud H. (2011). Eur. J. Med. Chem..

[cit27] Gomha S. M., Abdel-Aziz H. A. (2012). Bull. Korean Chem. Soc..

[cit28] Radwan M. A. A., Ragab E. A., Sabry N. M., El-Shenawy S. M. (2007). Bioorg. Med. Chem..

[cit29] Batchelor M. J. , Moffat D. F. C., Davis J. M. and Hutchings M. C., *US Pat.* US2002/147339, 2002

[cit30] Huynh T.-K.-C., Ngo K.-K.-H., Nguyen H.-P., Dang H.-K., Phung V.-T., Thai K.-M., Hoang T.-K.-D. (2021). New J. Chem..

[cit31] Yin Z.-G., Liu X.-W., Wang H. J., Zhang M., Liu X.-L., Zhou Y. (2022). New J. Chem..

[cit32] Ratcliffe A. J. , Alam M., Beevers R. E., Davenport R. J., Davies N., Haughan A. F., Jones M. W., Low C., Perry B. G., Phillips D. J., Pitt W. R. and Sharpe A., WO2006/38001, 2006

[cit33] Govender T., Maguire G. E. M., Krugera H. G., Shiri M. (2013). Curr. Org. Synth..

[cit34] Fadda A. A., EI-Mekabaty A., Mousa I. A., Elattar K. M. (2014). Synth. Commun..

[cit35] Kobayashi G., Furukawa S., Matsuda Y., Washida Y. (1967). Chem. Pharm. Bull..

[cit36] Gorbunova V. P., Suvorov N. N. (1978). Chem. Heterocycl. Compd..

[cit37] Kreher R., Wagner P. H. (1980). Chem. Berichte.

[cit38] Slatt J., Romero I., Bergman J. (2004). Synthesis.

[cit39] Abdel-Motaleb R. M., Makhloof A.-M. A.-S., Ibrahim H. M., Elnagdi M. H. (2007). J. Heterocyclic Chem..

[cit40] Venkatanarayana M., Dubey P. K. (2013). Indian J. Chem..

[cit41] Sivakumar S., Kanchithalaivan S., Kumar R. R. (2013). RSC Adv..

[cit42] Chen T., Xu X.-P., Ji S.-J. (2013). J. Heterocyclic Chem..

[cit43] Song P., Zhao L., Ji S. (2014). Chin. J. Chem..

[cit44] Borah P., Naidu P. S., Majumder S., Bhuyan P. J. (2014). Mol. Divers..

[cit45] Kamalraja J., Perumal P. T. (2014). Tetrahedron Lett..

[cit46] Kalalawe V., Kagne R., Munde D. (2016). Int. J. Innovative Res. Sci. Eng. Technol..

[cit47] Wang J., Liu H., Wen R., Zhu Z., Li J., Zhu S. (2017). Res. Chem. Intermed..

[cit48] Mohammadi Rasooll M., Zarei M., Zolfigol M. A., Sepehrmansourie H., Omidi A., Hasani M., Gu Y. (2021). RSC Adv..

[cit49] Zhou W., Huang Q., Shen L., Han J., Chen J., He W., Deng H., Shao M., Zhang H., Cao W. (2021). Synlett.

[cit50] Geng L.-J., Feng G.-L., Zhang H.-L., Zhang Y.-M. (2013). J. Chem. Res..

[cit51] Zeng L.-Y., Cai C. (2013). Synth. Commun..

[cit52] Nataraj P., Muralidharan D., Perumal P. T. (2013). Tetrahedron Lett..

[cit53] Bharkavi C., Gunasekaran P., Kumar S. V., Sakthi M., Perumal S. (2014). Tetrahedron Lett..

[cit54] Fatma S., Singh D., Ankit P., Mishra P., Singh M., Singh J. (2014). Tetrahedron Lett..

[cit55] Muthu M., Priya R. V., Almansour A. I., Kumar R. S., Kumar R. R. (2018). Beilstein J. Org. Chem..

[cit56] Venkateshan M., Priya R. V., Muthu M., Suresh J., Kumar R. R. (2019). Chem. Data Collect..

[cit57] Krishnammagari S. K., Balwe S. G., Kim J. S., Lim K. T., Jeong Y. T. (2019). Monatsh. Chem..

[cit58] Abo-Salem H. M., El Salam H. A. A., Abdel-Aziem A., Abdel-Aziz M. S., El-Sawy E. R. (2021). Molecules.

[cit59] Torabi M., Zolfigol M. A., Yarie M., Gu Y. (2021). Mol. Catal..

[cit60] Haldar Animeshchandra G. M., Chhajed Santosh S. (2016). J. Chem. Chem. Sci..

[cit61] Bhale P. S., Chavan H. V., Shringare S. N., Khedkar V. M., Tigote R. M., Mali N. N., Jadhav T. D., Kamble N. B., Kolat S. P., Bandgar B. P., Patil H. S. (2022). Synth. Commun..

[cit62] Fatma S., Singh D., Mishra P., Singh P. K., Ankit P., Singh M., Singh J. (2013). RSC Adv..

[cit63] Fatma S., Singh D., Mishra P., Singh P. K., Ankit P., Sing J. (2013). J. Chem. Res..

[cit64] Ahmad I., Mishra N. K., Ghosh T. (2013). Macrocycl. Chem..

[cit65] Bhale P. S., Bandgar B. P., Dongare S. B., Shringare S. N., Sirsat D. M., Chavan H. V. (2019). Phosphorus Sulfur Silicon Relat. Elem..

[cit66] Mamaghani M., Tabatabaeian K., Bayat M., Hossein Nia R., Rassa M. (2013). J. Chem. Res..

[cit67] Sepehrmansourie H., Zarei M., Taghavi R., Zolfigol M. A. (2019). ACS Omega.

[cit68] Afsar J., Zolfigol M. A., Khazaei A., Zarei M., Gu Y., Alonso D. A., Khoshnood A. (2020). Mol. Catal..

[cit69] Kalhor S., Zarei M., Sepehrmansourie H., Zolfigol M. A., Shi H., Wang J., Arjomandi J., Hasani M., Schirhagl R. (2021). Mol. Catal..

[cit70] Sepehrmansourie H., Zarei M., Zolfigol M. A., Babaee S., Azizian S., Rostamnia S. (2022). Sci. Rep..

[cit71] El-Mekabaty A., Hasel A. M. (2015). Chem. Heterocyclic Compd..

[cit72] Krishnammagari S. K., Cho B. G., Kim J. T., Jeong Y. T. (2018). Synth. Commun..

[cit73] El-Mekabaty A., Etman H. A., Mosbah A. (2016). J. Heterocyclic Chem..

[cit74] Kheirkhah L., Mamaghani Ma., Mahmoodi N. O., Yahyazadeh A., Fallah Shojaei A., Rostamli Y. (2016). J. Chin. Chem. Soc..

[cit75] Rangel J., Díaz-Uribe C., Rodriguez-Serrano A., Zarate X., Serge Y., Vallejo W., Nogueras M., Trilleras J., Quiroga J., Tatchen J., Cobo J. (2017). J. Mol. Struct..

[cit76] Mamaghani M., Sheykhan M., Sadeghpour M., Tavakoli F. (2018). Monatsh. Chem..

[cit77] Wang J., Zhu S., Liu Y., Zhu X., Shi K., Li X., Zhu S. (2022). Synth. Commun..

[cit78] Radwan M. A. A., Alminderej F. M., Awad H. M. (2020). Molecules.

[cit79] Radwan M. A. A., Alminderej F. M., Tolan H. E. M., Awad H. M. (2020). J. Appl. Pharm. Sci..

[cit80] Abdollahi-Basir M. H., Mirhosseini-Eshkevari B., Zamani F., Ghasemzadeh M. A. (2021). Sci. Rep..

[cit81] Dashteh M., Baghery S., Zolfigol M. A., Khazaei A. (2022). Mol. Diversity.

[cit82] Kumar A., Katiyar S., Kant R., Sashidhara K. V. (2020). Tetrahedron.

[cit83] Balwe S. G., Kim J. S., Kim Y.-I., Jeong Y. T. (2019). Tetrahedron.

[cit84] Huang W., Liu F., Wang K., Sidorenko A., Bei M., Zhang Z., Fang W., Li M., Gu Y., Ke S. (2022). Green Synth. Catal..

[cit85] Cai Q., Sheng H.-Y., Li D.-K., Liu Y., Wu A.-X. (2018). Synlett.

[cit86] Baharfar R., Azimi R., Asdollahpour Z. (2018). Environ. Chem. Lett..

[cit87] Baharfar R., Azimi R., Asdollahpour Z., Bagheri H. (2018). Res. Chem. Intermed..

[cit88] Azimi R., Baharfar R., Bagheri H. (2022). Polycycl. Aromat. Compd..

[cit89] Kuamr D., Kumar N. M., Tantak M. P., Ogura M., Kusaka E., Ito T. (2014). Bioorg. Med. Chem. Lett..

[cit90] Venkatanarayana M., Dubey P. K. (2013). J. Heterocyclic Chem..

[cit91] Basha K. N. U., Gnanamani S., Shanmugam P., Venugopal S., Murthy S., Ramasamy B. (2021). J. Heterocyclic Chem..

[cit92] Baharfar R., Asghari S., Kiani M. (2015). Monatsh. Chem..

[cit93] Naidu P. S., Kolita S., Majumder S., Bhuyan P. J. (2015). Synthesis.

[cit94] Reddy M. V., Reddy G. C. S., Jeong Y. T. (2016). Tetrahedron Lett..

[cit95] Kumar M. A., Sridhara A. M., Gururaja R., Yogisha S. (2016). Pharma Chem..

[cit96] Li L., Xu H., Dai L., Xi J., Gao L., Rong L. (2017). Tetrahedron.

[cit97] Li H.-L., Chen J., Chen D.-S., Shi P., Liu J.-Y. (2018). Heterocycl. Commun..

[cit98] Zhang W.-T., Niu F.-X., Yue R.-X., Zhang Y., Ma C., Sun J., Rong L. (2022). J. Heterocyclic Chem..

[cit99] Fadda A., Kandeel E. E., El-Gendy E. (2017). Pigm. Resin Technol..

[cit100] Al-Zaydi K. M., Mekheimer R. A., Mousally S. M., Borik R. M., Elnagdi M. H. (2017). Arab. J. Chem..

[cit101] Boruah D. J., Maurya R. A., Yuvaraj P. (2020). Results Chem..

[cit102] Garcia A. C., Abonia R., Jaramillo-Gomez L. M., Cobo J., Glidewell C. (2017). Acta Cryst..

[cit103] Treuer A. V., De-La-Torre P., Gutierrez M. I. (2017). J. Chem..

[cit104] Solangi M., Mohammed Khan K. K., Saleem F., Hameed S., Iqbal J., Shafique Z., Qureshi U., Ul-Haq Z., Taha M., Perveen S. (2020). Bioorg. Med. Chem..

[cit105] Wang H., Wang Z., Wang Y.-L., Zhou R.-R., Wu G.-C., Yin S.-Y., Yan X., Wang B. (2017). Org. Lett..

[cit106] Zhou T., Li B., Wang B. (2017). Chem. Commun..

[cit107] Xiao Y., Xiong H., Sun S., Yu J., Cheng J. (2018). Org. Biomol. Chem..

[cit108] Chodvadiya V. D., Pambhar K. D., Parmar N. D., Dhamsaniya A. P., Safi S. K. A., Chhatbar P. V., Ram H. N., Khunt R. C., Patel P. K. (2019). World Sci. News.

[cit109] Liu J., Chen Y., Yao B., Cai S., Li X., Leng Y., Cai X. (2022). Tetrahedron Lett..

